# In Situ Modulation of NiFeOOH Coordination Environment for Enhanced Electrocatalytic‐Conversion of Glucose and Energy‐Efficient Hydrogen Production

**DOI:** 10.1002/advs.202412872

**Published:** 2024-12-11

**Authors:** Ning Wei, Sufeng Zhang, Xue Yao, Qinglu Li, Nan Li, Jinrui Li, Dingjie Pan, Qiming Liu, Shaowei Chen, Scott Renneckar

**Affiliations:** ^1^ National Demonstration Center for Experimental Light Chemistry Engineering Education Shaanxi Provincial Key Laboratory of Papermaking Technology and Specialty Paper Development College of Bioresources Chemical and Materials Engineering Shaanxi University of Science and Technology Xi'an Shaanxi 710021 China; ^2^ Advanced Renewable Materials Lab Faculty of Forestry The University of British Columbia Vancouver BC V6T 1Z4 Canada; ^3^ Department of Chemistry and Biochemistry University of California 1156 High Street Santa Cruz CA 96064 USA; ^4^ Department of Chemistry Rice University Houston TX 77005 USA

**Keywords:** doping, electrocatalytic‐conversion of glucose, electrochemical activation, electrolyzer, hydrogen evolution reaction, NiFeOOH

## Abstract

Glucose electrocatalytic‐conversion reaction (GCR) is a promising anode reaction to replace the slow oxygen evolution reaction (OER), thus promoting the development of hydrogen production by electrochemical water splitting. Herein, NiFe‐based metal‐organic framework (MOF) is used as a precursor to prepare W‐doped nickel‐iron phosphide (W‐NiFeP) nanosheet arrays by ion exchange and phosphorylation, which exhibit a high electrocatalytic activity toward the hydrogen evolution reaction (HER), featuring an overpotential of only −179 mV to achieve the current density of 100 mA cm^−2^ in alkaline media. Notably, electrochemical activation of W‐NiFeP facilitates the in situ formation of phosphate groups producing W,P‐NiFeOOH, which, in conjunction with the W co‐doped amorphous layers, leads to a high electrocatalytic performance toward GCR, due to enhanced proton transfer and adsorption of reaction intermediates, as confirmed in experimental and theoretical studies. Thus, the two‐electrode electrolyzer of the W‐NiFeP/NF||W,P‐NiFeOOH/NF for HER||GCR needs only a low cell voltage of 1.56 V to deliver 100 mA cm^−2^ at a remarkable hydrogen production efficiency of 1.86 mmol h^−1^, with a high glucose conversion (98.0%) and formic acid yields (85.2%). Results from this work highlight the significance of the development of effective electrocatalysts for biomass electrocatalytic‐conversion in the construction of high‐efficiency electrolyzers for green hydrogen production.

## Graphical abstract

In situ electrochemical activation promotes the formation and electronic structure modulation of the amorphous NiFeOOH with W and P codoping, improves the electrochemical performance of GCR, and further accelerates the development of hydrogen production by electrolysis of water. The results demonstrated that the co‐doping of W and P effectively regulates the covalence of metal‐O and proton‐electron transfer.

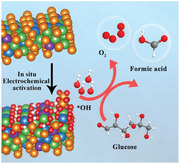

## 1 Introduction

Development of effective technologies for green hydrogen production represents a critical step toward a sustainable hydrogen economy. One option is electrochemical water splitting, which entails hydrogen evolution reaction (HER) at the cathode and oxygen evolution reaction (OER) at the anode.^[^ ^1^ ^]^ Yet, the performance is largely limited by the sluggish electron‐transfer kinetics and complex reaction pathways of OER.^[^ ^2^, ^3^ ^]^ In fact, OER consumes more than 90% of the total energy, with an overpotential (η_10,OER_) at least +200 mV to produce the current density of 10 mA cm^−2^ (i.e., an electrode potential over +1.45 V versus reversible hydrogen electrode (RHE)); additionally, the produced low‐value O_2_ is prone to serious safety hazards when mixed with H_2_.^[^ ^4^ ^]^ Therefore, it is of fundamental and technological significance to substitute OER with a facile counterpart so as to greatly enhance the electrolyzer performance in clean and safe hydrogen production.^[^ ^5^, ^6^, ^7^ ^]^ One viable option is the electrocatalytic‐conversion of biomass molecules, such as urea,^[^ ^8^ ^]^ hydrazine,^[^ ^9^ ^]^ ammonia,^[^ ^10^ ^]^ alcohols,^[^ ^11^, ^12^ ^]^ and furfural,^[^ ^13^ ^]^ that typically occurs at a much lower electrode potential with appropriate catalysts. For instance, Wang et al. prepared a NiF_3_‐Ni_2_P heterostructure grown in situ on carbon cloth (NiF_3_/Ni_2_P@CC) by hydrothermal and sequential fluoride phosphorization, which exhibited good electrocatalytic activity toward urea electrocatalytic‐conversion, requiring a potential of 1.36 V to deliver a current density of 10 mA cm^−2^.^[^ ^14^ ^]^ In contrast to urea that only produces low value‐added products upon electrocatalytic‐conversion,^[^ ^15^ ^]^ glucose is a biomass monosaccharide widely found in nature, has a low redox potential and can be selectively converted to formate, which has found diverse applications in agriculture, pharmaceutical, rubber, leather, textile, and food industries.^[^ ^16^, ^17^ ^]^ Therefore, electrolyzers based on the combination of glucose electrocatalytic‐conversion (GCR) and HER have been attracting extensive attention for efficient hydrogen production due to a low energy consumption and concurrent upgrading conversion of biomass to high value‐added chemicals. Within this context, rational design and engineering of high‐performance catalysts for GCR and HER represents a critical first step. Currently, noble metals have remained the electrocatalysts of choice; yet, the high costs and low reserves limit their industrial applications.^[^ ^11^, ^18^ ^]^ Therefore, development of low‐cost and efficient multifunctional electrocatalysts is crucial for the development of the electrolyzer system. In recent years, transition metal‐based nanomaterials have emerged as promising bifunctional electrocatalysts for GCR and HER due to their abundant reserves, tunable electronic structure, and high intrinsic activity.^[^ ^12^, ^19^, ^20^ ^]^ Among these, nickel‐based phosphides have attracted much attention, in particular, for HER with an activity comparable to that of commercial Pt/C.^[^ ^21^, ^22^, ^23^ ^]^ For instance, Lv et al. reported a facile cation exchange strategy for atomic‐level surface engineering of self‐supported nickel phosphide (NiP) nano‐arrays to produce Ni*_x_* Co*_1‐x_* P that exhibited an increase of the active sites and intrinsic electrocatalytic activity toward HER.^[^ ^24^ ^]^

Nickel‐based phosphides have also been found to be electrocatalytically active toward GCR. Nevertheless, glucose is a polyhydroxy aldehyde, and GCR entails the breaking of C‐H and O‐H bonds and complex proton‐electron reactions.^[^ ^25^, ^26^ ^]^ For nickel‐based phosphides, the actual active sites for GCR are typically the reversibly transformed M^n^/M^n+1^ couples of the metal centers (n ≥ 2) involved in a reversible deprotonation cycle.^[^ ^27^, ^28^ ^]^ Recent studies have shown that electrochemical activation can induce surface remodeling and electronic rearrangement in the active sites of transition metal catalysts, subtly tailoring the structure of the metal d band and oxygen p band, leading to significant enhancement of the GCR activity.^[^ ^29^ ^]^ For example, Wu et al. reported that after 500 potential cycles within the potential range of 1.0 to 1.5 V versus RHE, the surface of FeCoNiCuCuCr‐LDH could be electrochemically activated, where electronic structural rearrangement led to the formation of defect‐rich surfaces and hence an excellent GCR performance.^[^ ^30^ ^]^ Xia et al. observed controllable surface reconstruction of NiCrO composites by cation doping and oxygen vacancy generation, resulting in the modulation of the metal and lattice oxygen coordination and hence the adsorption energetics of key reaction intermediates.^[^ ^11^ ^]^

Herein, W‐doped nickel‐iron phosphide nanosheet arrays (W‐NiFeP/NF) were synthesized on nickel foam surfaces with NiFeMOF, a nickel‐based metal‐organic framework (MOF, PDF#35‐1677), as the precursor by solution heating, ion exchange, and low‐temperature phosphatization. Under moderate electronic structure modulation, W‐NiFeP/NF exhibited an HER performance comparable to noble metal electrocatalysts at high current densities. Furthermore, electrochemical activation at high electrode potentials led to the oxidation of elemental phosphorus to phosphoric acid groups and formation of high‐valence W^6+^, and hence cooping of W and P into the resulting amorphous NiFeOOH. The obtained W,P‐NiFeOOH/NF exhibited a three‐dimensional hierarchical structure and a large specific surface area, a structural feature conducive to accessibility of the catalytic active sites and mass transfer of reaction intermediates and electrolytes. Importantly, the codoping of W and P led to the redistribution of electrons around the active sites, which facilitated the proton‐coupled electron transfer during GCR and optimized the adsorption and activation of the reaction intermediates. Electrochemical measurements showed that W‐NiFeP/NF and W,P‐NiFeOOH/NF exhibited an excellent activity toward HER and GCR, needing an electrode potential of ‐0.179 and +1.362 V to produce a large current density of 100 mV cm^−2^, respectively. In the two‐electrode electrolyzer for hydrogen production, only a low cell voltage of 1.56 V was required to reach 100 mA cm^−2^, in comparison to 1.789 V for OER‐based water splitting. Notably, the high hydrogen production rate (1.86 mmol h^−1^) and Faraday efficiency, as well as the high formic acid yield could be retained after a 50‐cycle test. These results confirm the significance of electrochemical activation and heterogeneous doping in the design and engineering of efficient catalysts for biomass‐based electrolyzers in green hydrogen production and biomass upgrading electrocatalytic‐conversion.

## 2 Results and Discussion

### 2.1 DFT Calculations

**Figure 1:**
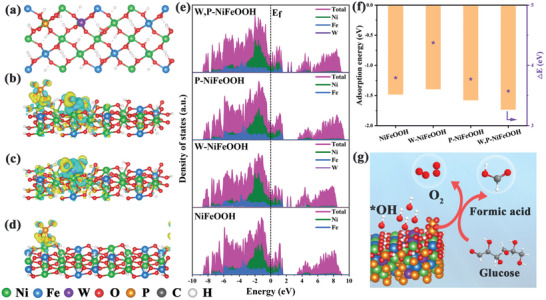
DFT calculations. a) Top view of the optimized structure of W,P‐NiFeOOH. Bader charge diagrams of the optimized structure of b) W,P‐NiFeOOH, c) W‐NiFeOOH, and d) P‐NiFeOOH, where the cyan areas indicate electron loss and yellow areas indicate electron gain. The isovalue of the charge density is 0.008 e Å^−3^. e) Density of states (DOS) and projected density of states (PDOS) of NiFeOOH, W‐NiFeOOH, P‐NiFeOOH, and W,P‐NiFeOOH. f) Glucose and the energy differences (ΔE) between *ε_d_* and the *p* band center (*ε_p_*) on the NiFeOOH, W‐NiFeOOH, P‐NiFeOOH, and W,P‐NiFeOOH. g) Schematic diagram of the enhancement effect of W,P‐NiFeOOH on OER and GCR.

DFT calculations were first performed to examine the impacts of W and P doping at the Ni and Fe sites on the electrocatalytic activity of W,P‐NiFeOOH. Optimal structural models of NiFeOOH, W‐NiFeOOH, P‐NiFeOOH, and W,P‐NiFeOOH were first constructed and depicted in **Figure** **1**a and Figure S1 (Supporting Information), with the corresponding Bader charge analysis shown in Figure 1b–d. The charge density difference of W‐NiFeOOH, P‐NiFeOOH, and W,P‐NiFeOOH clearly showed single W and P doping and their synergistic modulation of electronic rearrangement induced by the Ni and Fe sites in the NiFeOOH structure. In particular, electron dissipation was induced in the Ni and Fe sites by W and P co‐doping, resulting in a decrease of the localized electron cloud density around them, which implies that Ni and Fe exhibit elevated valence states. This result indicates that the electronic structure and chemical environment of the W,P‐NiFeOOH electrocatalysts are optimized, resulting in improved OER and GCR activities. Figure 1e depicts the partial density of state (PDOS) of the optimized NiFeOOH, W‐NiFeOOH, P‐NiFeOOH, and W,P‐NiFeOOH, which all exhibit a zero bandgap structure, suggesting good metallic characteristics that are conducive to electron transfer and hence electrocatalytic reactions. In addition, distinct unoccupied states are found above the Fermi energy level, which provide possible pathways for electron leaps and are susceptible to electron transfer.^[^ ^31^ ^]^ This electronic state increases the redox properties of Ni and Fe sites and contributes to the proton‐coupled electron transfer of the GCR process.^[^ ^32^ ^]^

Furthermore, the *d* band centers (*ε_d_*) can be tailored by W and P doping of the Ni and Fe sites, which varies at ‐1.811, ‐1.774, ‐1.853, and ‐1.818 eV for NiFeOOH, W‐NiFeOOH, P‐NiFeOOH, and W,P‐NiFeOOH, respectively. The fact that W,P‐NiFeOOH exhibits a moderate *ε_d_* indicates a balanced adsorption coverage and efficiency, which may be optimal for the electrocatalytic activity.^[^ ^33^, ^34^ ^]^ In addition, the energy differences (ΔE) between *ε_d_* and the *p* band center (*ε_p_*) can be found to vary from NiFeOOH (3.794 eV) to W‐NiFeOOH (4.376 eV), P‐NiFeOOH (3.772 eV), and W,P‐NiFeOOH (3.572 eV), which suggests that W,P‐NiFeOOH possesses the strongest transition‐metal 3d‐O 2p orbital hybridization and hence M‐O covalency (Figure 1f; Table S1, Supporting Information). A high M‐O covalency has been known to promote electron transfer between adsorption sites and oxygen‐containing reactants, thus accelerating the electrocatalytic process.^[^ ^35^, ^36^ ^]^ In GCR, the reaction involves both a direct and indirect pathway of glucose electrocatalytic‐conversion both of which are associated with glucose adsorption on the active site. Therefore, a high M‐O covalency can modulate the binding energy of the active site to glucose, leading to enhanced GCR activity. In fact, the adsorption energy of glucose (Figures S2 and S3, Supporting Information) on W,P‐NiFeOOH (‐1.734 eV) is significantly higher than those of NiFeOOH (‐1.484 eV), W‐NiFeOOH (‐1.396 eV), and P‐NiFeOOH (‐1.579 eV), indicating energetically favored adsorption and activation of glucose on W,P‐NiFeOOH (Figure 1f). In addition, the adsorption energy of glucose on W,P‐NiFeOOH was much larger than that of OH* (Figure S4, Supporting Information), implying the superiority of the glucose electrocatalytic conversion reaction.

Taken together, the results suggest that W and P codoping of W,P‐NiFeOOH results in the formation of an optimized electronic structure, with a moderate d‐band center, that facilitates the adsorption and activation of glucose and rapid electron transfer, leading to enhanced GCR performance.

### 2.2 Structural Characterization

**Figure 2:**
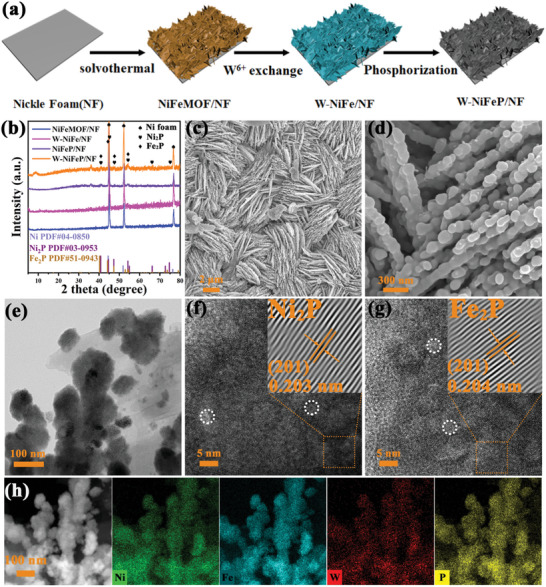
a) Schematic of the synthesis of W‐NiFeP/NF. b) XRD patterns of NiFeMOF/NF, W‐NiFe/NF, NiFeP/NF, and W‐NiFeP/NF. c,d) SEM and e–g) TEM images of W‐NiFeP/NF. Insets to (f) and (g) are the corresponding IFFT patterns of the orange boxes. h) Element maps of Ni, Fe, W, and P in W‐NiFeP/NF.

The synthesis process of W‐NiFeP/NF is described schematically in **Figure** **2**a which consists of three major steps, solvothermal treatment, cation exchange, and low‐temperature chemical vapor phosphating. Briefly, NiFeMOF nanosheet arrays were in situ grown on nickel foam (NF) surface via a hydrothermal method. The NiFeMOF precursors were then converted into W‐doped NiFe/NF (W‐NiFe/NF) nanosheet arrays by cation‐exchange with sodium tungstate. Finally, W‐doped NiFeP (W‐NiFeP/NF) nanosheet arrays were obtained by low‐temperature chemical vapor‐phase phosphorylation of W‐NiFe/NF using PH_3_ generated from thermal decomposition of sodium hypophosphite. Such a structural evolution was also manifested in a color change of the sample surface (Figure S5, Supporting Information).

The materials structures were first examined by X‐ray diffraction (XRD) measurements by scratching the samples off the NF substrate. The NiFeMOF precursor (Figure S6, Supporting Information) and W‐NiFe (Figure 2b), can be seen to exhibit a set of diffraction patterns that are consistent with those of nickel‐based MOF (PDF#35‐1677), indicating minimal impacts of W doping on the crystal structure of NiFeMOF. Yet for W‐NiFeP and NiFeP, additional diffraction peaks appeared at 2θ = 40.79°, 44.60°, 47.31° and 54.23° that can be attributed to the (111), (201), (210) and (300) crystal planes of Ni_2_P and Fe_2_P,^[^ ^37^ ^]^ suggestive of successful formation of metal phosphides in the samples.

Further structural insights were obtained from scanning electron microscopy (SEM) and transmission electron microscopy (TEM) measurements. NiFeMOF/NF can be seen to consist of nanosheet arrays, featuring a nanoflake morphology (Figure S7, Supporting Information). Yet the flaky arrays became denser on the sample surface after cation exchange with W^6+^ (W‐NiFe/NF, Figure S8, Supporting Information). After phosphorylation, the nanosheets were transformed into arrays of NiFeP and W‐NiFeP nanorods (Figure 2c–e; Figure S9, Supporting Information). Nitrogen sorption measurements (Figures S9–S11, Supporting Information) show that the samples were enriched with micropores, and the specific surface area increased from 3.06 m^2^ g^−1^ for NiFeMOF/NF to 4.91 m^2^ g^−1^ for W‐NiFe/NF, 6.21 m^2^ g^−1^ for NiFeP/NF and 12.27 m^2^ g^−1^ for W‐NiFeP/NF. This can be ascribed to the production of highly corrosive PH_3_ during the phosphating process that etched the NiFe‐MOF/NF and W‐NiFe/NF samples. Furthermore, well‐defined lattice fringes can be resolved in high‐resolution TEM measurements (Figure 2f,g and inset), where the inverse Fast Fourier transform (IFFT) patterns demonstrated an interplanar spacing of ca. 0.203 and 0.204 nm that can be assigned to the (201) crystallographic plane of Ni_2_P and Fe_2_P, respectively.^[^ ^38^ ^]^ In EDS‐based elemental mapping analysis, the Ni (15.12%), Fe (3.51%), W (0.37%), and P (13.42%) elements can be found to be evenly distributed across the samples (Figure 2h).

The elemental composition and valency of W‐NiFeP/NF were further investigated by X‐ray photoelectron spectroscopy (XPS) measurements. From the full spectra in Figure S12 (Supporting Information), the elements of Ni 2p, Fe 2p and O 1s electrons can be readily resolved at ca. 870, 720, and 530 eV for NiFeMOF/NF, respectively, whereas the additional W 4f peak can be found at 37 eV for W‐NiFe/NF, P 2p at 135 eV for NiFeP/NF, and both are visible for W‐NiFeP/NF, confirming successful doping of W and/or P into the samples. The high‐resolution scans of the Ni 2p electrons are shown in Figure S13a (Supporting Information). NiFeMOF/NF can found to possess two doublets, a major one at 854.97/872.47 eV and a minor one at 857.07/875.27 eV that can be ascribed to the 2p_3/2_/2p_1/2_ electrons of Ni^2+^ and Ni^3+^ (along with a satellite doublet at 861.57/880.07 eV), respectively.^[^ ^39^ ^]^ After W^6+^ ion exchange and phosphating, these binding energies increased to 855.97/873.47 eV and 858.07/875.97 eV for W‐NiFe/NF, and 856.07/873.77 eV and 858.77/876.57 eV for NiFeP/NF, suggesting electron depletion of Ni upon W and P doping. In addition to these Ni species, W‐NiFeP/NF and NiFeP/NF both possessed an additional doublet at 852.57 eV that can be attributed to the Ni 2p_3/2_ electrons of Ni‐P.^[^ ^37^ ^]^ The corresponding Fe 2p spectra are shown in Figure S13b (Supporting Information). NiFeMOF/NF can be found to possess two doublets at 706.97 eV/720.27 eV that can be assigned to the 2p_3/2_/2p_1/2_ electrons of Fe^3+^ (along with a satellite doublet at 715.37 and 727.87 eV), respectively; and these binding energies increased to 707.87/721.47 eV for W‐NiFe/NF, 708.27/722.07 eV for NiFeP/NF and 708.47/722.27 eV for W‐NiFeP/NF,^[^ ^40^ ^]^ again, signifying electron depletion of Fe upon W and P doping. Figure S13c (Supporting Information) shows the high‐resolution scans of the W 4f electrons, where the W 4f_5/2_/4f_7/2_) doublet can be resolved at 37.97/35.87 eV for W‐NiFeP and W‐NiFe/NF, confirming the successful incorporation of W^6+^ in materials structure by ion exchange.^[^ ^28^ ^]^ The P 2p spectra for NiFeP/NF and W‐NiFeP/NF are shown in Figure S13d (Supporting Information), where deconvolution yields a major species at 134.37 eV due to P‐O and a minor doublet at 129.97 and 130.97 eV for metal‐P.^[^ ^41^ ^]^ This confirms the formation of metal phosphides as shown in the above TEM measurements.

Results from these analyses confirmed that W and P were indeed doped into the NiFe oxide scaffold, which led to effective electron depletion of the Ni and Fe sites. In addition, metal phosphides were produced in the samples.

### 2.3 HER Performance of W‐NiFeP/NF

**Figure 3:**
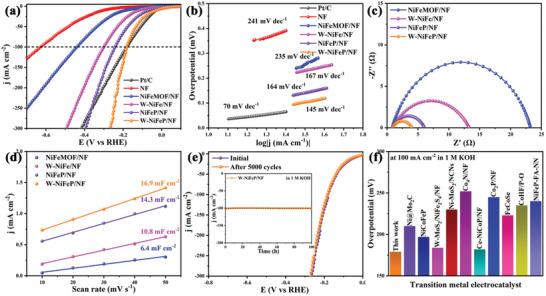
a) HER polarization curves and b) Tafel plots of Pt/C, NF, NiFeMOF/NF, W‐NiFe/NF, NiFeP/NF, and W‐NiFeP/NF in 1 M KOH. c) Nyquist plots and d) the double‐layer capacitances (C_dl_) measurements of NiFeMOF/NF, W‐NiFe/NF, NiFeP/NF, and W‐NiFeP/NF. e) HER LSVs of W‐NiFeP/NF before and after 5000 CV cycles. Inset is the chronoamperometry curves of W‐NiFeP/NF at ‐0.18 V versus RHE to deliver a current density of 100 mA cm^−2^ for 100 h. f) Comparison of overpotentials at 100 mA cm^−2^ for W‐NiFeP/NF with leading results reported in the literature.

The HER performance of the obtained samples was then tested and compared in 1 M KOH at room temperature. **Figure** **3**a shows the HER polarization curves of Pt/C, NF, NiFeMOF/NF, W‐NiFe/NF, NiFeP/NF, and W‐NiFeP/NF. It can be seen that W‐NiFeP/NF required an overpotential (η_HER_) of ‐133, ‐179, and ‐227 mV to reach the current density of 50, 100, and 200 mA cm^−2^, respectively, which were much lower than those of NF (over ‐600 mV), NiFe‐MOF/NF (−336, −441, and −636 mV), W‐NiFe/NF (−230, −300, and −405 mV), and NiFeP/NF (‐176, ‐242, and ‐328 mV), but slightly subpar as compared to those of commercial Pt/C (−109 mV at 50 mA cm^−2^) (Figure S14, Supporting Information). This suggests effective HER activity of the NiFe nanosheet arrays and the activity was markedly enhanced by W and P doping, with W‐NiFeP/NF being the best catalyst among the series.

The corresponding Tafel plots are shown in Figure 3b, where the Tafel slope can be estimated to be 145 mV dec^−1^ for W‐NiFeP/NF, which is significantly lower than those of NF (241 mV dec^−1^), NiFe‐MOF/NF (235 mV dec^−1^), W‐NiFe/NF (167 mV dec^−1^), and NiFeP/NF (164 mV dec^−1^), implying facile electron‐reaction kinetics within the sample series. In addition, the Tafel slope of 145 mV dec^−1^ suggests that the Heyrovsky reaction was likely the rate‐determining step (RDS), and HER followed the Volmer‐Heyrovsky pathway on W‐NiFeP/NF.^[^ ^42^ ^]^

Consistent results were obtained from electrochemical impedance measurements. From the Nyquist plots in Figure 3c, W‐NiFeP/NF can be seen to exhibit the lowest charge transfer resistance (*R_ct_*) of 3.0 Ω, as compared to 4.9 Ω for NiFe‐MOF/NF, 12.3 Ω for W‐NiFe/NF, and 22.2 Ω for NiFeP/NF. In addition, W‐NiFeP/NF possessed a significantly larger double layer capacitance (C_dl_) of 16.9 mF cm^−2^ than NiFe‐MOF/NF (6.4 mF cm^−2^), W‐NiFe/NF (10.8 mF cm^−2^), and NiFeP/NF (14.3 mF cm^−2^), suggesting enhanced electrochemically active surface area (ECSA) and accessibility of the electrocatalytic active sites (Figure 3d; Figure S15, Supporting Information). This is in good agreement with the formation of a porous structure in W‐NiFeP/NF, as manifested in microscopic and nitrogen sorption measurements (Figures S10 and S11, Supporting Information).

In terms of ECSA(electrochemically active surface area)‐normalized current densities (Figure S16a, Supporting Information) and turnover frequency (TOF, Figure S16b, Supporting Information), W‐NiFeP/NF also outperformed others in the sample series, suggesting high intrinsic HER activity. The cyclic stability and long‐term durability of the catalyst were then evaluated by cyclic voltammetry and chronopotentiometry measurements. From Figure 3e, the polarization curve of W‐NiFeP/NF remained almost unchanged after 5000 consecutive CV cycles from 0.2 to −0.7 V versus RHE; and in chronopotentiometric measurements (inset to Figure 3e), the overpotential of W‐NiFeP/NF stayed virtually invariant at the current density of 100 mA cm^−2^ for 100 h, implying its excellent stability. In fact, no marked variation of the sample morphology and chemical structure was observed in SEM and XPS measurements (Figures S17 and S18, Supporting Information). Significantly, the MOF‐derived W‐NiFeP/NF exhibited an electrocatalytic performance that was superior to those of relevant catalysts reported in the literature (Figure 3f).

The HER properties of W‐NiFeP/NF exhibited strong dependence on the W and phosphorus contents and phosphatization temperature (Figures S19–S30, Supporting Information), and the optimal samples were those under the above discussion.

### 2.4 W,P‐NiFeOOH/NF by In Situ Electrochemical Activation of W‐NiFeP/NF

**Figure 4:**
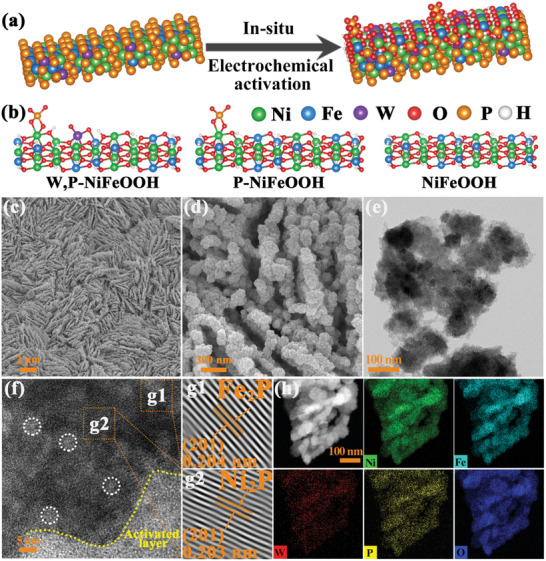
a) Schematic representation of in situ electrochemical activation of W‐NiFeP/NF in 1 M KOH. b) Structural models of W,P‐NiFeOOH, P‐NiFeOOH and NiFeOOH. c,d) SEM images and e,f) TEM and HRTEM images of W,P‐NiFeOOH/NF, The white circles represent lattice defects. g1,g2) IFFT patterns of the orange boxes in (f). h) Elemental mapping of Ni, Fe, W, P, and O in W,P‐NiFeOOH/NF.

Remarkably, the W‐NiFeP/NF sample also exhibited an apparent activity toward GCR after in situ electrochemical activation (**Figure** **4**a), which was carried out in 1 M KOH for 50 CV cycles between 0.82 and 1.82 V versus RHE (Figure S31, Supporting Information). When the applied potential was over +1.4 V, the phosphorus species on the catalyst surface was oxidized to terminal phosphate anionic species (P), producing amorphous W,P‐codoped NiFeOOH (W,P‐NiFeOOH). For comparison, NiFeOOH was formed by in situ electrochemical activation of NiFeMOF, W‐NiFeOOH from W‐NiFe, and P‐NiFeOOH from NiFeP (Figure 4b; Figure S32, Supporting Information). Note that W,P‐NiFeOOH/NF retained the original nanosheet array structure of W‐NiFeP/NF, while the nanosheets became roughened and denser on the sample surface, as evidenced in SEM (Figure 4c,d) and TEM measurements (Figure 4e). Moreover, electrochemical activation led to the formation an amorphous layer of NiFeOOH (Figure 4f), whereas lattice fringes can be readily resolved in the interior of the material, as shown in the IFFT patterns of Figure 3g1,g2 and FFT images of Figure S33 (Supporting Information). EDS‐based elemental mapping analysis showed a uniform distribution of the Ni, Fe, W, P, and O elements in the W,P‐NiFeOOH/NF sample (Figure 4h).

**Figure 5:**
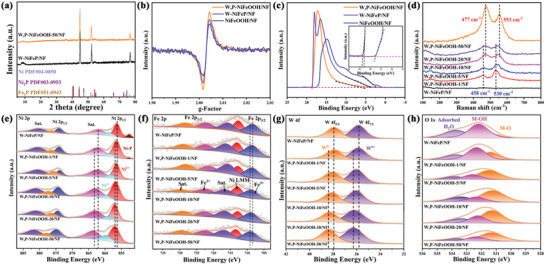
a) XRD patterns of W‐NiFeP/NF after 50 CV cycles electrochemical activation in 1 M KOH. b) EPR spectra and c) UPS spectra of valance band for NiFeOOH, W‐NiFeP/NF and W,P‐NiFeOOH. d) Raman spectra of W‐NiFeP/NF before and after electrochemical activation. High‐resolution XPS of e) Ni 2p, f) Fe 2p, g) W 4f, and h) O 1s spectra of W‐NiFeP/NF after various CV cycles electrochemical activation 1 M KOH.

From **Figure** **5**a, One can see that after electrochemical activation, the XRD patterns of W,P‐NiFeOOH were essentially the same as those of W‐NiFeP/NF (virtually no change for up to 50 CV cycles), suggesting that the electrochemical activation occurred only on the sample surface and the crystalline structure of W‐NiFeP/NF was retained. Furthermore, in electron spin resonance (ESR) measurements (Figure 5b), NiFeOOH/NF, W‐NiFeP/NF, and W,P‐NiFeOOH/NF all displayed a strong signal at g = 2.003, with the peak‐to‐peak intensity increasing in the order of NiFeOOH/NF < W‐NiFeP/NF < W,P‐NiFeOOH/NF, suggesting the formation of increasingly abundant oxygen vacancies^[^ ^43^ ^]^ — note that oxygen vacancies can enhance the adsorption and migration of oxygen‐containing species on the catalyst surface, thereby improving the OER and GCR activities.^[^ ^44^ ^]^

Figure 5c illustrates the ultraviolet photoelectron spectroscopy (UPS) analysis of W,P‐NiFeOOH/NF, W‐NiFeP/NF and NiFeOOH/NF. Notably, the cutoff energy (E_cutoff_) was found to decrease from 18.68 eV for W,P‐NiFeOOH/NF to 18.53 eV for W‐NiFeP/NF, and to 16.93 eV for NiFeOOH/NF. As work function (WF) = hν – E_cutoff_ + E_Fermi_ (where hν = 21.2 eV and E_Fermi_ = 0 eV),^[^ ^45^, ^46^ ^]^ the work function of W,P‐NiFeOOH/NF can be estimated to be 2.52 eV, significantly lower than those of W‐NiFeP/NF (2.67 eV) and NiFeOOH/NF (4.27 eV), suggesting weaker electron‐binding ability that facilitates electron donation from the catalyst surface to reaction intermediates. This also suggests enhanced internal conductivity of W,P‐NiFeOOH/NF. In addition, the valence band maximum (VBM) of W,P‐NiFeOOH/NF can be found at 5.6 eV, as compared to 3.7 eV for W‐NiFeP/NF and 2.3 eV for NiFeOOH/NF. Due to the major contributions of the 3d orbitals to the Fermi level energy, the shift of the valence bands suggests that the d‐band of W,P‐NiFeOOH/NF also shifted in comparison to those of W‐NiFeP and NiFeOOH/NF.^[^ ^47^ ^]^

To further determine the formation of NiFeOOH, quasi‐in situ Raman spectroscopy was performed with W‐NiFeP/NF before and after electrochemical activation after different CV cycles. As shown in Figure 5, no obvious characteristic peaks were observed for the initial W‐NiFeP/NF. After one and five CV cycles, the bending and stretching peaks at 458 and 530 cm^−1^ can be clearly observed due to M‐OH, as W‐NiFeP/NF underwent rapid remodeling in electrochemical activation.^[^ ^47^ ^]^ With a further increase of the CV cycles (up to 50), these peaks were gradually replaced by the ones at 477 and 553 cm^−1^ due to the bending and stretching of MOOH, confirming the reconstruction of W‐NiFeP/NF into W,P‐NiFeOOH/NF after prolonged electrochemical activation.^[^ ^48^ ^]^ In fact, one can see that after electrochemical activation for 50 CV cycles, the peaks located at 477 and 553 cm^−1^ for the reconstructed W,P‐NiFeOOH/NF exhibited larger peak height ratios, implying a high valence state of M for W,P‐NiFeOOH/NF.^[^ ^20^ ^]^

These structural variations reflect changes of the electronic structure of W,P‐NiFeOOH/NF, due to in situ electrochemical activation and doping of W and P. XPS measurements were employed to further investigate the details of chemical composition and valence states of electrocatalysts during electrochemical activation. Figure 5d depicts the high‐resolution scans of the Ni 2p electrons, where the 2p_3/2_ peak characteristic of Ni‐P can be resolved at 852.57 eV which was gradually weakened after 10 CV cycles, most likely due to surface electrochemical activation.^[^ ^17^ ^]^ The peaks at 856.37 (Ni^2+^) and 862.07 eV (Sat.) also shifted to higher binding energies, suggesting the formation of increasingly electron‐deficient Ni species that was conducive to the production of NiOOH.^[^ ^49^ ^]^ Concurrently, the Ni^3+^ 2p_3/2_ peak at 858.97 eV became gradually intensified, indicating an increase of the Ni^3+^ content.^[^ ^40^ ^]^ Similarly, the Fe^3+^ 2p_3/2_ peak can be found at 708.47 eV, and after 50 CV cycles, shifted slightly to a higher binding energy with a gradual increase of the content, due to the formation of FeOOH (Figure 5e).^[^ ^50^ ^]^

The W 4f spectrum of W,P‐NiFeOOH/NF (Figure 5f) can be convoluted into two subpeaks corresponding to the 4f_5/2_ and 4f_7/2_ electrons of W^6+^ (35.77 and 37.97 eV), respectively.^[^ ^51^ ^]^ After 50 CV cycles, the W peak also shifted to a higher binding energy, suggesting that electrochemical activation similarly increased the W valence state, thereby favoring the optimization of the electronic structures of the Ni and Fe sites. In the high‐resolution P‐2p scans (Figure S34a, Supporting Information), the peaks at 129.87 and 130.67 eV correspond to the 2p_3/2_ and 2p_1/2_ electrons of P‐M,^[^ ^52^ ^]^ respectively, which diminished during the electrochemical activation, while the relative content of P‐O gradually increases (Table S2, Supporting Information), indicating that the P‐M species were converted into phosphate anions and embedded in the NiFeOOH structure. In addition, the P 2p high‐resolution depth profile XPS scan of W,P‐NiFeOOH‐50/NF showed that the P electrons of W,P‐NiFeOOH‐50/NF were similar before and after etching by 10 nm, suggesting that the phosphate anions were stably present in the amorphous layer of W,P‐NiFeOOH‐50/NF without the presence of P‐M species (Figure S35, Supporting Information). In addition, the O 1s spectra were deconvoluted into three characteristic peaks, M‐O (531.25 eV), M‐OH (532.47), and adsorbed‐H_2_O (533.75 eV), where the peak area of M‐O gradually increased with the deepening of electrochemical activation (Figure S34b, Supporting Information).

The chemical compositions and valence states of NiFeMOF/NF, W‐NiFe/NF, and NiFeP/NF after electrochemical activation were also investigated and compared, as shown in Figures S36–S38 (Supporting Information). In particular, compared to NiFeOOH, W‐NiFeOOH and P‐NiFeOOH, the XPS peaks of both Ni and Fe in W,P‐NiFeOOH were significantly positively shifted, and the binding energies followed the following order: W,P‐NiFeOOH > P‐NiFeOOH > W‐NiFeOOH > NiFeOOH. The results indicated that during in situ electrochemical activation, W and P doping synergistically optimized the electronic structure of W,P‐NiFeOOH/NF, and all the metal elements were oxidized to higher valence states.

### 2.5 GCR and Overall Water Splitting Performance

**Figure 6:**
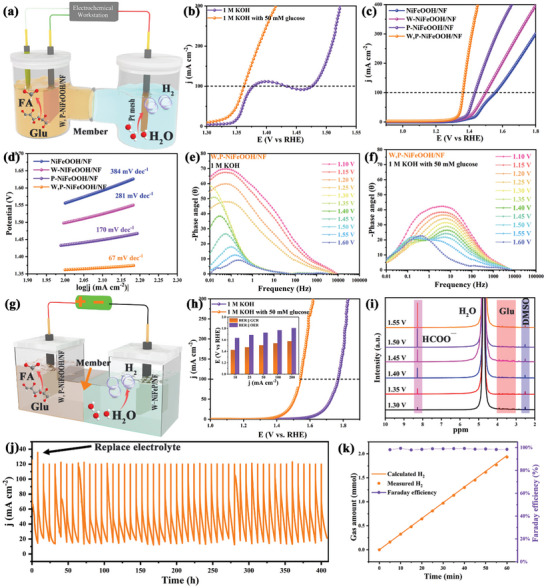
GCR and glucose assisted overall water splitting performance. a) Schematic diagram of three‐electrode system of GCR for W,P‐NiFeOOH/NF in H‐type electrolytic cell. b) Polarization curves of W,P‐NiFeOOH/NF in 1 M KOH with and without 50 mM glucose. c) Polarization curves and d) Tafel slopes for NiFeOOH/NF, W‐NiFeOOH/NF, P‐NiFeOOH/NF, and W,P‐NiFeOOH/NF in 1 M KOH with 50 mM glucose. Bode plots of in situ EIS data at various voltages of W,P‐NiFeOOH/NF for e) OER and f) GCR. g) Schematic diagram of an electrolytic cell of glucose assisted overall water splitting for W‐NiFeP/NF||W,P‐NiFeOOH/NF. h) Polarization curves of W‐NiFeP/NF||W,P‐NiFeOOH/NF in 1 M KOH with and without 50 mM glucose. Insert: The comparison of overpotentials of W‐NiFeP/NF||W,P‐NiFeOOH/NF at various current densities in 1 M KOH with and without 50 mM glucose. i) ^1^H NMR measurements of glucose converted to formate at varied potentials. j) The Chronoamperometric curves of W‐NiFeP/NF||W,P‐NiFeOOH/NF in 1 M KOH with 50 mM glucose under 50 cycles. k) Amount of hydrogen and Faraday efficiency of the electrolytic water system assisted by electrocatalytic‐conversion of glucose.

Replacing OER at the anode with GCR not only reduces the energy input of hydrogen production from overall water splitting and significantly improves the efficiency of hydrogen production, but also enables the upgrading and electrocatalytic‐conversion of biomass to produce high value‐added chemicals. The electrocatalytic performance of W,P‐NiFeOOH for GCR was examined in a standard three‐electrode configuration in 1 M KOH with 50 mM glucose (**Figure** **6**a). Remarkably, the voltage required to achieve the same current density decreased dramatically after the addition of glucose. Specifically, the potential required for W,P‐NIFeOOH/NF to achieve the current densities of 100 and 200 mA cm^−2^ in GCR were +1.362 and +1.386 V, respectively, which were significantly lower than those of +1.476 and +1.508 V for OER (Figure 6b). In fact, GCR exhibited higher current densities than OER within a wide range of potential (Figures S39–S51, Supporting Information) and for an extended period of time (up to 20 h). For GCR, W,P‐NiFeOOH/NF exhibited a lower Tafel slope of 67 mV dec^−1^ than OER (102 mV dec^−1^) (Figure S52, Supporting Information), implying superior electrocatalytic reaction kinetics for GCR, which was attributed to the strong adsorption of glucose and GCR reaction intermediates to the electrocatalyst surface (Section 2.1).^[^ ^11^ ^]^

The electrochemical activation and in situ W and P modulation of the coordination environments of Ni and Fe sites were explored to promote the glucose electrocatalytic‐conversion performance. W,P‐NiFeOOH/NF required a low potential of +1.362 V to deliver a current density of 100 mA cm^−2^, whereas 1.556, 1.499, and 1.434 V for NiFeOOH/NF, W‐NiFeOOH/NF, P‐NiFeOOH/NF, respectively, confirming the excellent GCR electrocatalytic activity of W,P‐NiFeOOH/NF (Figure 6c), which is consistent with the DFT results (Section 2.1). The Tafel slope of W,P‐NiFeOOH/NF was only 67 mV dec^−1^ compared to those of NiFeOOH/NF (384 mV dec^−1^), W‐NiFeOOH/NF (281 mV dec^−1^), and P‐NiFeOOH/NF (170 mV dec^−1^), implying excellent electron‐transfer kinetics. Specially, the Tafel slope of 67 mV dec^−1^ for W,P‐NiFeOOH/NF was lower than 120 mV dec^−1^, suggesting excellent adsorption and activation of glucose on W,P‐NiFeOOH/NF (Figure 6d).^[^ ^11^ ^]^ In particular, as shown in Figure S53 (Supporting Information), W,P‐NiFeOOH/NF exhibited higher TOF than other samples in the series at the same voltage, implying that in situ W and P doping enhanced the intrinsic activity for glucose electrocatalytic‐conversion.

To further investigate the effect of W, P doping on the OER and GCR electrocatalytic interfacial properties of W, P‐NiFeOOH, *operando* EIS measurements were carried out with NiFeOOH/NF, W‐NiFeOOH/NF, P‐NiFeOOH/NF and W,P‐NiFeOOH. From the Nyquist plots (Figures S54 and S55, Supporting Information), the W,P‐NiFeOOH/NF electrode can be seen to exhibit the lowest R_ct_ of OER and GCR, and the fastest decreases of R_ct_ with increasing electrode potentials, which was consistent with the change of Tafel slope, indicating that W, P‐NiFeOOH/NF was more easily polarized.^[^ ^27^ ^]^ Furthermore, the elevated potential provided richer electrons and adsorbed hydroxyl groups, leading to faster charge transfer efficiency. At the low‐frequency convergence of the OER bode plot, the frequencies corresponding to the phase peaks of NiFeOOH/NF, W‐NiFeOOH/NF, and P‐NiFeOOH/NF phase peaks were shifted to higher frequencies at a faster rate, indicating that more hydroxyl groups need to be adsorbed on their surfaces to balance the hydroxyl depletion rate, implicitly suggesting stronger hydroxyl adsorption behavior (Figure S56, Supporting Information).^[^ ^12^, ^53^ ^]^ The excellent hydroxyl adsorption capacity and depletion rate of the W,P‐NiFeOOH/NF during OER suggests that in situ W and P modulation of the Ni and Fe sites optimized the electronic structure of W,P‐NiFeOOH/NF with excellent OER performance (Figure 6e). The absence of characteristic peaks attributed to the oxidation of metal species in the mid‐frequency range of W,P‐NiFeOOH/NF further suggests that W and P modulation altered the electron cloud density of the Fe and Ni active centers of W,P‐NiFeOOH/NF, where the hypervalent oxidation state enhanced the activity.^[^ ^6^ ^]^ For GCR, all electrodes showed completely different characteristic peaks in the mid‐frequency region from those observed during OER (Figure 6f; Figure S57, Supporting Information). As the voltage increased, the corresponding frequencies changed and the peaks shifted to a lower phase, proving that they were related to the adsorption of glucose and OH onto the electrode surface. The Bode plot of W,P‐NiFeOOH/NF slowly shifted from 1.30 to 1.60 V, proving that the W and P doping caused strong glucose adsorption on the Ni and Fe sites of W,P‐NiFeOOH/NF. However, when the voltage was increased to 1.50 V, a characteristic peak associated with OER appeared at low frequencies. This is attributed to the decrease of the concentration of glucose on the surface of W,P‐NiFeOOH/NF caused by the rapid electrocatalytic‐conversion at high potentials. And due to the reversible oxidation of MOH/MOOH (M^n+^/M^n+1+^, n ≥ 2) limited by low proton concentration, W,P‐NiFeOOH/NF was forced to maintain the high‐valence oxidation state, which is the active site of OER.^[^ ^18^, ^53^ ^]^ At the potential of 1.1 V, W,P‐NiFeOOH/NF exhibited a low R_ct_, indicating that glucose and OH* adsorbed on the electrocatalyst surface and that GCR followed a direct electrocatalytic‐conversion mechanism.^[^ ^30^, ^54^ ^]^ The electron release and oxygen uptake processes inside the catalyst form high‐valent metal species when the potential was gradually increased. At continuously increased potential, the active intermediates of high‐valent metal species on the electrocatalyst surface participated in the GCR process through the indirect electrocatalytic‐conversion mechanism.^[^ ^30^, ^54^ ^]^ Compared with NiFeOOH/NF, W‐NiFeOOH/NF, and P‐NiFeOOH/NF, the R_ct_ and phase angle of W,P‐NiFeOOH/NF were lower, indicating that W and P doping promoted the structural evolution of the W,P‐NiFeOOH/NF sample, which led to the generation of additional hydroxyl reactive species, accelerated electron transfer and promoted GCR at lower onset potentials.

As W‐NiFeP/NF exhibited an excellent HER activity and could serve as a GCR precatalyst, the electrocatalytic performance of a two‐electrode water electrolyzer was investigated by coupling GCR with HER using W‐NiFeP/NF as the cathodic electrocatalyst and W,P‐NiFeOOH/NF as the anodic electrocatalyst (Figure 6g). The required potential of GCR‐assisted overall water splitting system was only 1.56 V to deliver a high current density of 100 mA cm^−2^, which was much lower than conventional alkaline overall water splitting (1.789 V) (Figure 6h), and superior to most of the recently reported electrocatalysts for the electrocatalytic‐conversion of organics coupled with hydrogen production (Table S3, Supporting Information).

The anodic products collected from the GCR‐assisted total hydrolysis system were analyzed using proton nuclear magnetic resonance (^1^H NMR and ^13^C NMR) and high‐performance liquid chromatography (HPLC) measurements, and it was determined that the main product of the electrocatalytic‐conversion of glucose was formate (Figures S58–S60, Supporting Information). The ^1^H NMR and HPLC results of glucose electrocatalytic‐conversion at controlled potentials for different reaction times showed that the glucose electrocatalytic‐conversion product contained a large amount of formate and almost no oxalic, ethanolic, or gluconic acids, which suggests that W,P‐NiFeOOH/NF possessed a high selectivity for GCR to formate (Figure 6i; Figures S61 and S62, Supporting Information).

In addition, after electrolysis for 8 h in the GCR‐coupled water electrolyzer, the electrocatalytic‐conversion of glucose, the yield of formic acid and the Faraday efficiency of formic acid production were estimated to be 98%, 85.2% and 94.3% for W,P‐NiFeOOH/NF, respectively, which were significantly higher than those of NiFeOOH/NF (15.2, 8.1, 8.0%), W‐NiFeOOH/NF (37.9, 35.2, 31.8%) and P‐NiFeOOH/NF (65.3, 58.0, 55.4%), suggesting that W and P doping enhanced the GCR activity of W,P‐NiFeOOH/NF, as well as improved the charge utilization and generation rate of formate (Figure S63, Supporting Information).

Note that the W,P‐NiFeOOH based GCR‐coupled electrolyzer retained good glucose electrocatalytic‐conversion and hydrogen production efficiencies over 50 cycles. The calculated hydrogen production rate collected by the drainage method was 1.86 mmol h^−1^, the Faraday efficiency for hydrogen production was close to 100% (Figure 6k), and the high glucose electrocatalytic‐conversion, formic acid yield and Faraday efficiency at the anode were retained over five cycles (Figure S64, Supporting Information), suggesting excellent stability for the catalyst. This strategy could be further extended to the electrocatalytic conversion of other monosaccharides, such as arabinose, fructose, galactose, and xylose (Figure S65, Supporting Information), suggesting strategic advantages in energy‐saving hydrogen production and coupled generation of valuable chemicals. SEM and TEM results confirmed that the hierarchical nanosheet structure of W,P‐NiFeOOH/NF remained essentially unchanged after continuous GCR‐coupled electrolysis (Figure S66, Supporting Information). The interior of the nanosheets showed distortions of the Ni_2_P and Fe_2_P lattice fringes, which was encapsulated by an activated amorphous layer (Figure S66e1,e2, Supporting Information). XPS measurements showed that the chemical states of the metal elements were basically unchanged, indicating that W,P‐NiFeOOH/NF retained the initial high valence states of the metal species, and hence good reversible conversion of the active metal sites facilitated the indirect electrocatalytic‐conversion mechanism (Figure S67, Supporting Information).

### 2.6 Mechanism

**Figure 7:**
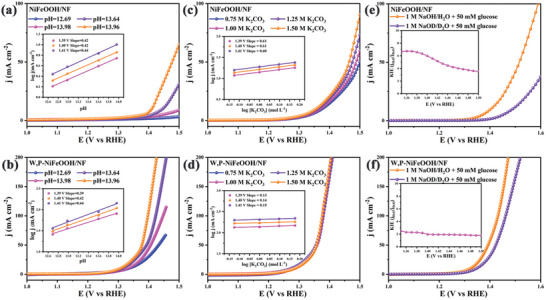
Polarization curves of a) NiFeOOH/NF and b) W,P‐NiFeOOH/NF in KOH and 50 mM glucose as a function of pH, insets: the correlation between GCR activity and pH value. Polarization curves of c) NiFeOOH/NF and d) W,P‐NiFeOOH/NF with varied concentrations of K_2_CO_3_ at pH 13.96, inset: the correlation between GCR activity and concentrations of K_2_CO_3_. Polarization curves of e) NiFeOOH/NF and f) W,P‐NiFeOOH/NF in 1.0 M NaOD/D_2_O and 1.0 M NaOH with 50 mM glucose, insets: KIEs value as a function of voltage.

The electrocatalytic‐conversion of glucose involves a multistep C─H and O─H bond breaking and multiple electron transfer and deprotonation processes. In order to gain a deeper understanding of the mechanism and kinetics of the reaction, the dependence of the catalytic activity of the prepared electrocatalysts was investigated in electrolytes of different pH.^[^ ^55^, ^56^ ^]^ The GCR activities of NiFeOOH/NF, W‐NiFeOOH/NF, P‐NiFeOOH/NF, and W,P‐NiFeOOH/NF were significantly enhanced with a gradual increase of pH from 12.69 to 13.96 (**Figure** **7**a,b; Figure S68, Supporting Information), suggesting that proton transfer during electrocatalytic processes was crucial for the GCR performance. Considering that buffer bases could exist as proton acceptors to facilitate the proton transfer process, thus lowering the reaction energy barriers, altering the reaction pathways, and improving the GCR kinetics. Therefore, the effect of the concentration of buffer base (K_2_CO_3_) in the electrolyte on the GCR activity was analyzed and showed in Figure 7c,d and Figure S69 (Supporting Information). The GCR activities of NiFeOOH/NF, W‐NiFeOOH/NF, P‐NiFeOOH/NF were increased while W,P‐NiFeOOH/NF did not undergo any significant change as the concentration of K_2_CO_3_ was gradually increased from 0.75 to 1.50 m, and W,P‐NiFeOOH/NF showed the smallest slope. The GCR electrocatalytic activity of W,P‐ NiFeOOH/NF was almost unaffected, suggesting that in situ W and P modulation optimized the electronic structure of W,P‐NiFeOOH/NF to exhibit excellent proton conduction, thereby attenuating the deprotonation of buffer bases in the electrolyte.

Deuterium kinetic isotope effects (KIEs) were investigated to further clarify the proton transfer process. Consider that proton migration in deuterated aqueous solutions is much slower than in various protonated aqueous electrolytes.^[^ ^57^, ^58^ ^]^ Thus proton transfer kinetics in D_2_O will be greatly scaled down. The KIEs of NiFeOOH/NF, W‐NiFeOOH/NF, P‐NiFeOOH/NF, and W,P‐NiFeOOH/NF were investigated in 1.0 m NaOD/D_2_O and 1.0 m NaOH with 50 mm glucose. The KIEs for all electrocatalysts were computed in the range of 1.35 to 1.50, since glucose can be converted by fully digesting the current. The GCR activities of NiFeOOH/NF, W‐NiFeOOH/NF, P‐NiFeOOH/NF, and W,P‐NiFeOOH/NF in aqueous 1.0 m NaOH and 50 mm glucose were superior to the activities in 1.0 m NaOD and 50 mm glucose D_2_O solutions (Figure 7e,f; Figure S70, Supporting Information). The KIEs values of electrocatalytic‐conversion of glucose in W,P‐NiFeOOH/NF were less than those of NiFeOOH/NF, W‐NiFeOOH/NF, P‐NiFeOOH/NF, indicating that the doping of W and P optimized the electronic structure of W,P‐NiFeOOH/NF and facilitated the deprotonation process and electron transfer of the GCR, which improved the electrocatalytic‐conversion of glucose.

From the literature, multiple the reaction pathways have been reported so far for the electrocatalytic conversion of polyhydroxy compounds to formate via C−C bond cleavage (Figure S71, Supporting Information), including 1) the aldose pathway^[^ ^59^, ^60^ ^]^ and 2) the most commonly used aldehyde pathway.^[^ ^5^, ^27^, ^61^ ^]^ Pathway 2 can be ruled out for the formation of formate from glucose, and it was reasonable to believe that the reaction started at the C1−C2 bond position of glucose, that is, electrocatalytic conversion of glucose began to produce arabinose and formate at the C1−C2 position, because this bond order was the lowest among the five C−C bonds in the glucose molecule (Figure S72, Supporting Information).

Based on these findings, previous reports and experimental results, it was proposed that the electrocatalytic conversion of glucose to formate was based on aldehydes as key intermediates and the cleavage of C−C bonds as a reaction prerequisite (Figure S73, Supporting Information).^[^ ^19^, ^62^, ^63^ ^]^ This cleavage mainly occurred at the edge of glucose, shortening one carbon atom at each step, gradually entering the route of 5C, 4C, glyceraldehyde and dihydroxyacetone, and removing a formate (i.e., the final product) at each step. However, the relative number of carbon atoms in the structure increases the complexity of the reaction mechanism and the challenges associated with maximizing product selectivity. Glucose could also be isomerized to fructose under alkaline conditions, or enter the glyceraldehyde route to ultimately produce formate esters. In addition, glyceraldehyde was further oxidized to glyceride or lactate, and lactate is then oxidized to tartaric acid ester or acetate and formates. Glyceraldehyde could also be isomerized to dihydroxyacetone, which was oxidized to lactate through functional groups, and finally to formate through two‐step C‐C bond cleavage (removal of formate).

The electrocatalytic conversion of glucose began with the spontaneous adsorption of glucose on the electrocatalyst surface. According to previous calculation results, the prepared electrocatalyst exhibited a strong adsorption capacity for glucose, which was the first step for the reaction to proceed effectively. Subsequently, the entire reaction involved a multi‐step proton‐electron transfer process, especially the reversible redox of the active site of the electrocatalyst and the electron transfer process of the reaction substrate as an electron donor. The catalytic active site for the electrocatalytic conversion of glucose became reduced to MOH (equation 1). Subsequently, under an applied voltage, the reversible redox of MOH/MOOH (M^n+^/M^n+1+^, n≥2) accompanied by the indirect electrocatalytic conversion of glucose played an important role (Equation (2)). The mechanism study showed that the doping of W and P optimized the electronic structure of W,P‐NiFeOOH/NF, promoted the deprotonation process and electron transfer of GCR, which effectively improved the electrocatalytic conversion rate of glucose.

(1) $$CH_{2}\mathrm{OH}-{\left(\mathrm{CHOH}\right)}_{4}-\mathrm{CHO}+\mathrm{MOOH}\to \mathrm{Product}+\mathrm{MOH}$$

(2) $$\mathrm{MOH}+OH^{-}\to H_{2}O+\mathrm{MOOH}+e^{-}$$

## 3 Conclusion

In this study, high‐performance W‐doped NiFeP electrocatalysts were successfully constructed on nickel foam substrates. The results showed that W doping not only modulated the microstructure of the nanosheets array, but also significantly improved the HER performance. Further studies confirmed that W and P was in situ doped into the amorphous NiFeOOH layer formed on the electrocatalyst surface after electrochemical activation, which effectively modulated the catalyst surface morphology, increased the number of exposed active sites, induced the electron rearrangement of Ni and Fe sites, optimized the adsorption behavior of W,P‐NiFeOOH/NF on reactive molecules, and simultaneously accelerated the proton‐electron transfer of the GCR process. DFT calculations demonstrated that W and P co‐doping effectively tuned the d‐band centers of the metal sites, leading to elevated metal‐O covalency and promoting the adsorption and catalytic activity of key reaction intermediates. The required potentials of W,P‐NiFeOOH/NF was only 1.362 and 1.386 V to provide current densities of 100 and 200 mA cm^−2^. Remarkably, the electrolyzer constructed with W,P‐NiFeOOH/NF as the CGR electrocatalyst and W‐NiFeP/NF as the HER catalyst required only 1.56 and 1.62 V to deliver the current densities of 100 and 200 mA cm^−2^, respectively, with high glucose conversion (98%), formic acid yield (85.2%), and hydrogen production rate (1.86 mmol h^−1^). Remarkably, the assembled electrolyzer remained stable after 50 consecutive cycles of electrolysis, which is superior to most of the transition metal‐based electrocatalysts reported so far. Results from this work highlight the prominent role of electrochemical activation in the design of biomass electrocatalytic upgrading‐coupled hydrogen production catalysts that possess a high activity and robust durability. This is critical for large‐scale production of high‐value‐added chemicals from biomass and green hydrogen generation.

## 4 Experimental Section

### Materials

Nickel nitrate hexahydrate (Ni(NO_3_)_2_⋅6H_2_O), iron(III) chloride hexahydrate (FeCl_3_·6H_2_O), sodium tungstate dihydrate (Na_2_WO_4_·2H_2_O), p‐phthalic acid (C_8_H_6_O_4_), sodium hypophosphite (NaH_2_PO_2_⋅H_2_O), potassium hydroxide (KOH), sodium hydroxide (NaOH), sodium deuteroxide (NaOD), potassium carbonate (K_2_CO_3_), glucose (C_6_H_12_O_6_), dimethylacetamide (DMAC), and deuterium oxide (D_2_O) were provided by Shanghai Aladdin Biochemical Technology Co. Commercial platinum carbon (20 wt.%, Pt/C), RuO_2_ electrocatalysts, and 5 wt.% Nafion solution were purchased from Suzhou Sinero Technology Co. Nickel foam (NF) was obtained from Saibo Chemical Material Technology Development Co., Ltd.

### Synthesis of NiFeMOF/NF Nanosheet Arrays

NiFeMOF/NF was synthesized using a facile solvothermal method.^[^ ^64^ ^]^ First, NF was cleaned with acetone and HCl (3 M) to remove surface oxides and impurities, rinsed several times with ultrapure water and anhydrous ethanol, and dried under vacuum at room temperature. Then, 116.3 mg of Ni(NO_3_)_2_⋅6H_2_O, 27 mg of FeCl_3_⋅6H_2_O, and 199 mg of C_8_H_6_O_4_ were dissolved sequentially in a solvent mixture of 28 mL of DMAC, 1 mL of deionized water, and 1 mL of anhydrous ethanol under magnetic stirring for 20 min before being transferred into a 50 mL Teflon‐lined stainless steel reactor. Finally, a piece of NF was immersed into the solution, which was heated at 150 °C for 6 h. After natural cooling to room temperature, the NiFeMOF/NF was removed from the solution, rinsed three times with water and ethanol, and dried under vacuum at 60 °C overnight.

### Synthesis of W‐NiFe/NF

For the preparation of W‐NiFe/NF, a cation exchange method was adopted in a solvothermal reaction. Briefly, 20 mg of Na_2_WO_4_·2H_2_O was dissolved in a solvent mixture of 15 mL of ion‐free water and 15 mL of anhydrous ethanol. Subsequently, the synthesized NiFeMOF/NF was impregnated into the above solution and transferred to a 50 mL Teflon‐lined stainless steel reactor and kept at 150 °C for 4 h.

### Synthesis of W‐NiFeP/NF and NiFeP/NF

W‐NiFeP/NF prepared using low‐temperature phosphide topography. The W‐NiFe/NF obtained above and NaH_2_PO_2_⋅H_2_O were placed downstream and upstream of the tube furnace, respectively. Then, the tube furnace was heated to 350 °C at a heating rate of 3 °C min^−1^ where the heating was kept for 2 h. When the tube furnace was cooled down to room temperature, W‐NiFeP/NF was obtained. NiFeP/NF were synthesized in the same manner but with NiFeMOF/NF as the precursor.

### Characterization

Scanning electron microscopy (SEM) measurements were conducted with a Zeiss scope with an accelerating voltage of 10 kV, and high‐angle annular dark‐field scanning transmission electron microscopy (HAADF‐STEM) images and elemental maps based on energy‐dispersive X‐ray spectroscopy (EDS) were acquired with a FEI Tecnai F20. X‐ray diffraction (XRD) analysis was carried out with a Bruker D8 ADVANCE diffractometer equipped with Cu K_α_ radiation at a scan rate of 6° min^−1^ within the 2θ range of 5° to 80°. X‐ray photoelectron spectroscopy (XPS) measurements were conducted with a Thermo Fisher ESCALAB 250Xi instrument. Electron spin resonance (ESR) analysis was performed on a JES FA200 spectrometer. UV photoelectron spectroscopy (UPS) studies were performed with a Thermo ESCALAB 250Xi instrument where photoelectrons were produced using 21.2 eV He plasma discharge.

### Electrochemistry

Electrochemical tests were conducted at room temperature with a CHI 760E electrochemical workstation using a typical three‐electrode system. The details of electrode preparation and conditions for electrochemical tests were included in the Supporting Information.

## Conflict of Interest

The authors declare no conflict of interest.

## Supporting information



Supporting Information

## Data Availability

Research data are not shared.
