# Supplementary material for: In Situ Modulation of NiFeOOH Coordination Environment for Enhanced Electrocatalytic‐Conversion of Glucose and Energy‐Efficient Hydrogen Production
Source: Adv Sci (Weinh). 2024 Dec 11;12(5):2412872. doi: 10.1002/advs.202412872 (PMC11792028; doi:10.1002/advs.202412872)
Supplement: Supplementary file 1 — Supporting Information [file ADVS-12-2412872-s001.docx]

Supporting Information

**In Situ Modulation of NiFeOOH Coordination Environment for Enhanced Electrocatalytic-Conversion of Glucose and Energy-Efficient Hydrogen Production**

*Ning Wei, Sufeng Zhang*, Xue Yao, Qinglu Li, Nan Li, Jinrui Li, Dingjie Pan, Qiming Liu, Shaowei Chen*, Scott Renneckar*

Ning Wei, Sufeng Zhang, Xue Yao, Qinglu Li, Nan Li, Jinrui Li

Shaanxi University of Science and Technology, Xi'an, National Demonstration Center for Experimental Light Chemistry Engineering Education, Shaanxi Provincial Key Laboratory of Papermaking Technology and Specialty Paper Development, College of Bioresources Chemical and Materials Engineering, 710021, Shaanxi, China

E-mail: zhangsufeng@sust.edu.cn

Dingjie Pan, Shaowei Chen

Department of Chemistry and Biochemistry, University of California, 1156 High Street, Santa Cruz, CA 96064, USA

E-mail: shaowei@ucsc.edu

Ning Wei, Xue Yao, Scott Renneckar

Advanced Renewable Materials Lab, Faculty of Forestry, The University of British Columbia, Vancouver, BC, V6T 1Z4, Canada

Qiming Liu

Department of Chemistry, Rice University, Houston, TX, 77005 USA

**List of Contents**

- Experimental section
- 64 figures
- 3 tables

# Experimental Section

**Electrochemical measurements**

All electrochemical measurements were conducted with a CHI760E electrochemical workstation with a standard three-electrode system in 1 M KOH at room temperature. The prepared electrocatalysts were used as the working electrode, a Pt plate as the counter electrode and a Hg/HgO (saturated KCl) electrode as the reference electrode. The Hg/HgO potential was calibrated against a reversible hydrogen electrode (RHE): E_RHE_ = E_Hg/HgO_ + 0.0591pH + 0.098. The OER, HER, and GCR performance of the fabricated electrocatalysts were measured via linear scanning voltammetry (LSV) at 90% *iR* compensation, where R was determined from the EIS results. The Tafel slopes were calculated according to the equation η = a + b log|j|. Electrochemical impedance tests were performed in the frequency range of 0.01 to 10^5^ Hz with an AC amplitude of 5 mV. Chronoamperometric tests were carried out at 100 mA cm^-2^ for 100 h in 1.0 M KOH.

The electrocatalytic conversion reaction of glucose was carried out in a two-electrode configuration in a solution containing 1.0 M KOH and 30 mM glucose The conversion products were analyzed using high-performance liquid chromatography (HPLC). Specifically, lactic acid and formic acid were determined using an HPLC system (Shimadu) equipped with a UV detector operating at 210 nm and a Bio-Rad Aminex HPX-87H column (300 mm × 7.8 mm × 9 μm). A aqueous solution of H_2_SO_4_ (5 mM) was used as an eluent at a flow rate of 0.6 mL min^-1^. The column was thermostated at 35°C using a column heater. A sampling loop of 20 µL was used. Glucose was measured using an RI detector. The ultrapure water was used as an eluent. The concentration of monosaccharide and product was determined by the standard curves established by external standard method. The glucose conversion yield was calculated by Conversion (%) = Moles of carbon in feedstock consumed/Moles of carbon in feedstock input × 100%; and product yield (%) = Moles of carbon in organic acid/Moles of carbon in feedstock input × 100%. The faradaic efficiency (FE) to electrocatalytic conversion products were calculated by FE (%) = (Mole of product formed × *n* × *F*)/Total charge passed × 100%, where n is the number of electron transfer for each product formed from glucose (n = 2 for formic acid, glycolic acid, and glyceric acid, whereas n = 6 for oxalic acid,) and F is the Faraday constant (96485 C mol^-1^).

**Calculation of electrochemically active surface area (ECSA)**

ECSA was estimated from the double layer capacitance (C_dl_), ECSA = C_dl_/C_S_ × S, where S is the geometric surface area of the electrode and C_s_ is the specific capacitance of the material per unit area under identical electrolyte conditions, which is 0.040 mF cm^-2^ in 1.0 M KOH solution based on previously reported Ni-based (NF) catalysts.

**Calculation of the turnover frequency (TOF)**

TOF is defined as the number of reaction products generated per active site per unit time. TOF of an electrocatalyst is an important kinetic parameter which indicates the intrinsic activity of the catalyst in electrochemical reaction. TOF is calculated from the following equation, TOF = *j*N_A_/mFn, where j is the current density, N_A_ stands for the Avogadro number, m is the number of electron transfer in the generation of a product molecule, F is the Faraday’s constant, and n is the number of atoms or active sites participating in the catalyst material ^[1-2]^.

**DFT calculations**

All first-nature principles are calculated based on the theory of spin polarization density in general gradient (GGA) through PerDew-Burke-ERNZERHOF (PBE). The predicted augmented wave (PAW) potentials are used to describe ion nuclei using a plane-wave basis set, and a plane-wave basis set with 450 eV is applied. The DFT+U correction is considered during the electronic structure calculations for systems with strongly correlated d electrons. The electronic energy is considered self-consistent when the energy change is smaller than 10^−5^ eV. A geometry optimization is considered convergent when the energy change is smaller than 0.02 eV Å^−1^. During the relaxation, the Brillouin zone with a 1 × 1 × 1 Gamma centered grid is used. A 25 Å vacuum layer is normally added to the surface to eliminate the artificial interactions between periodic images. Spin polarized calculations are performed for the calculations.


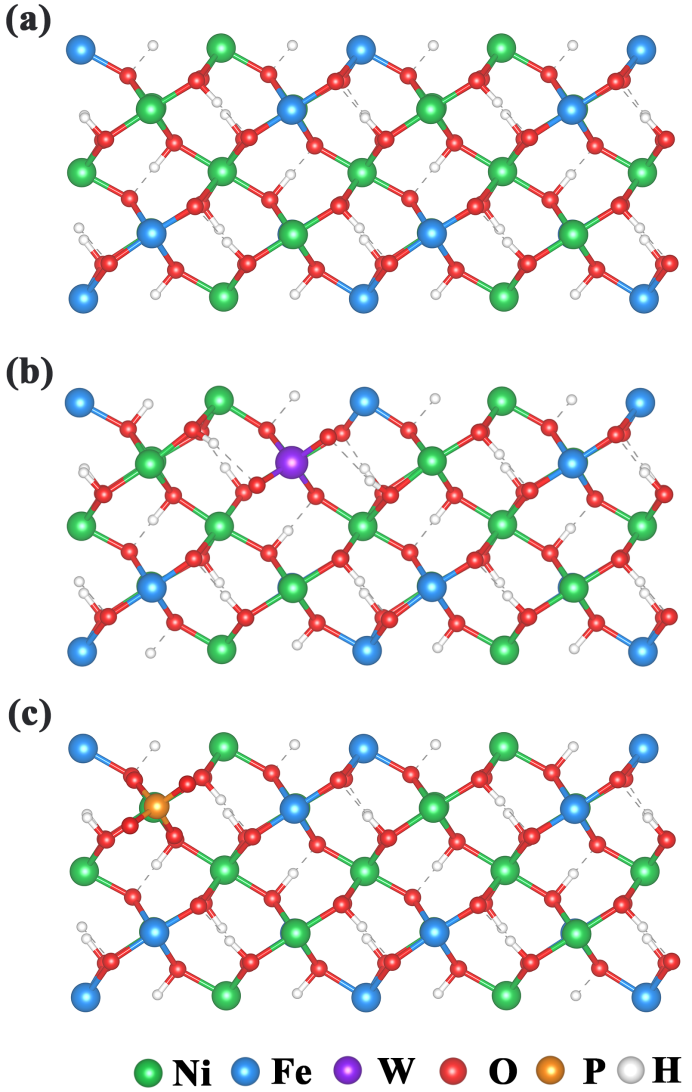


# Figure S1. Top view of the optimized structure models of (a) NiFeOOH, (b) W-NiFeOOH, and (c) P-NiFeOOH.

**
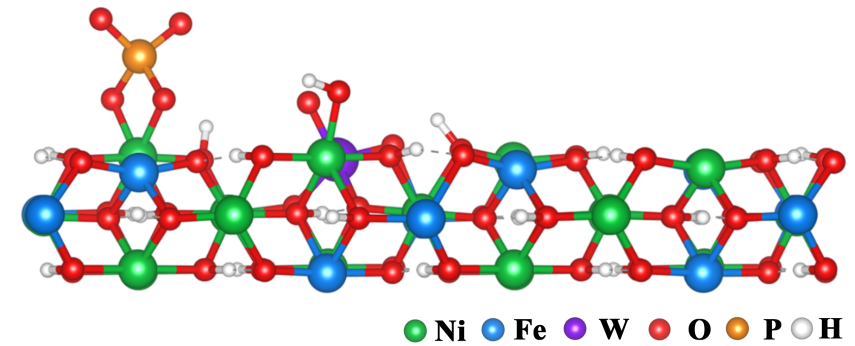
**

# Figure S2. Adsorption configuration of OH* on the surface W,P-NiFeOOH.


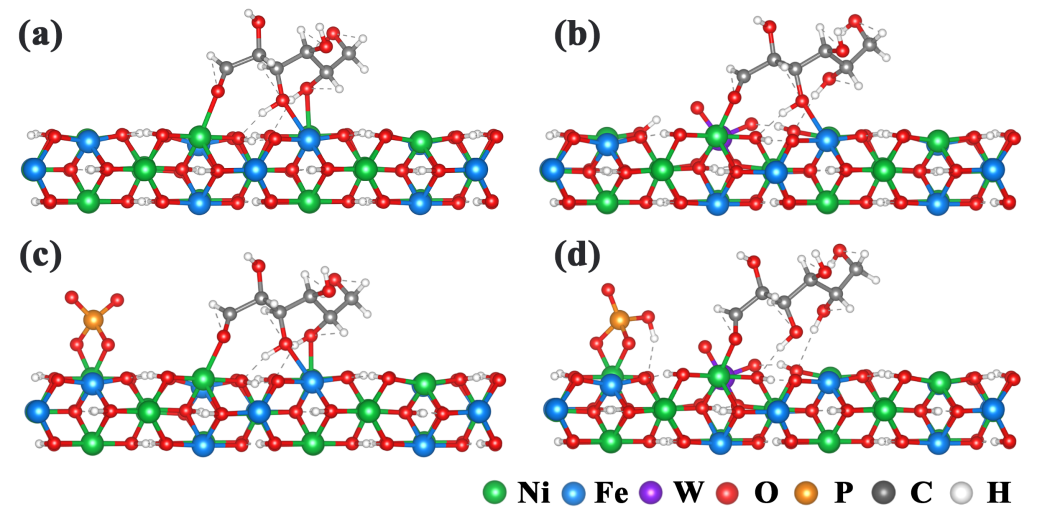


# Figure S3. Adsorption configuration of glucose on the surface of (a) NiFeOOH, (b) W-NiFeOOH, (c) P-NiFeOOH and (d) W,P-NiFeOOH.


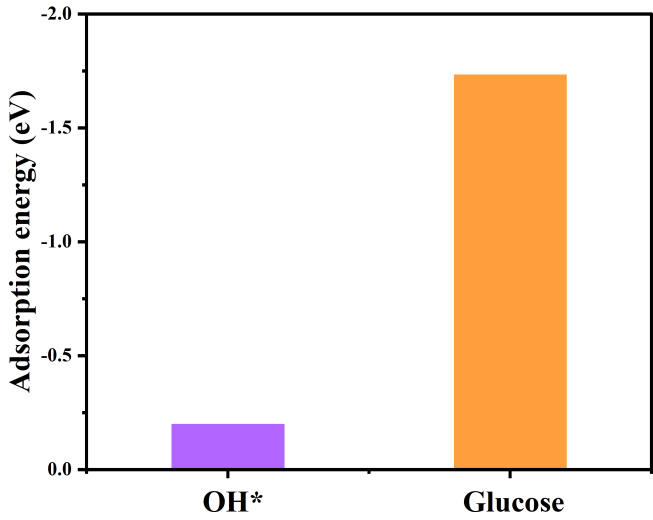


# Figure S4. OH* and Glucose adsorption energy on the W,P-NiFeOOH


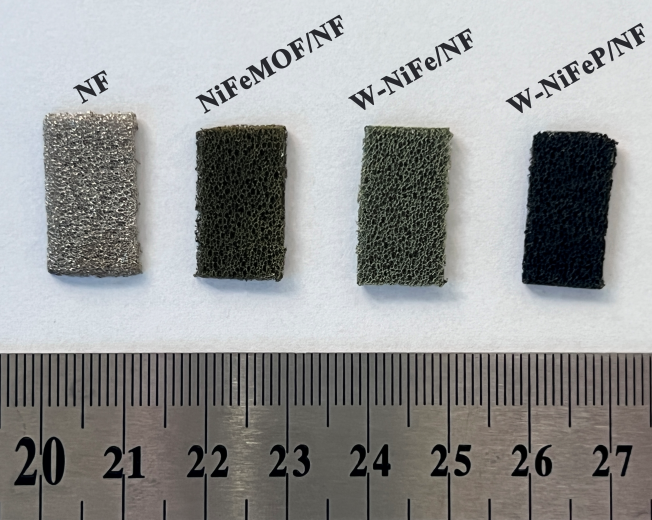


# Figure S5. Photograph of NF, NiFeMOF/NF, W-NiFe/NF, and W-NiFeP/NF.


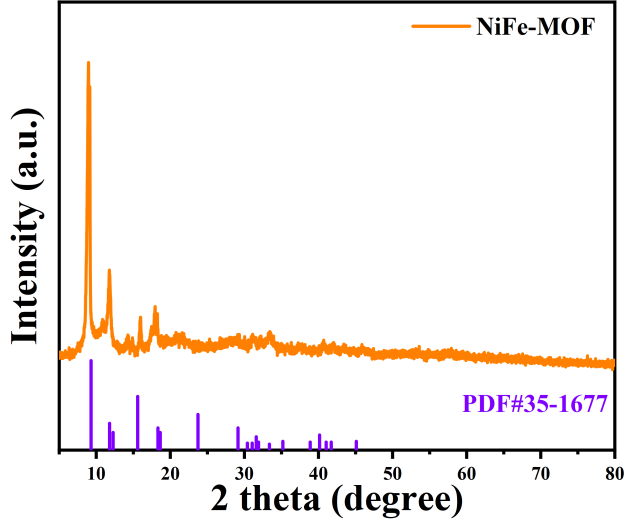


# Figure S6. XRD patterns of the NiFe-MOF powders.


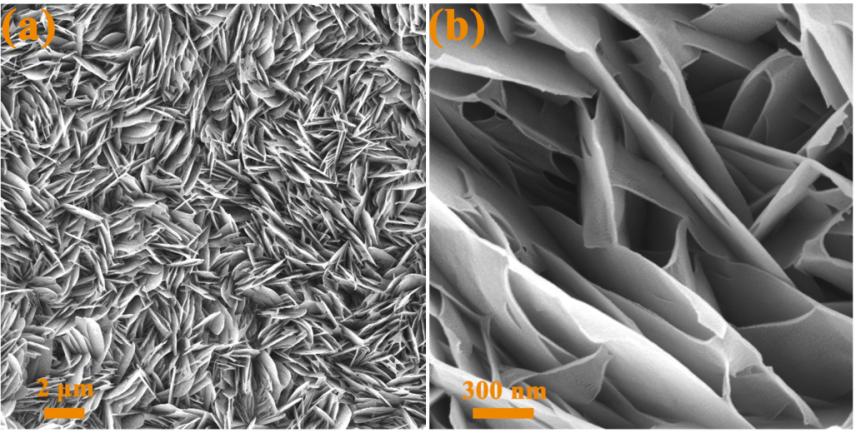


# Figure S7. SEM images of NiFe-MOF/NF at different magnifications. Scale bars are (a) 2 μm and (b) 300 nm.


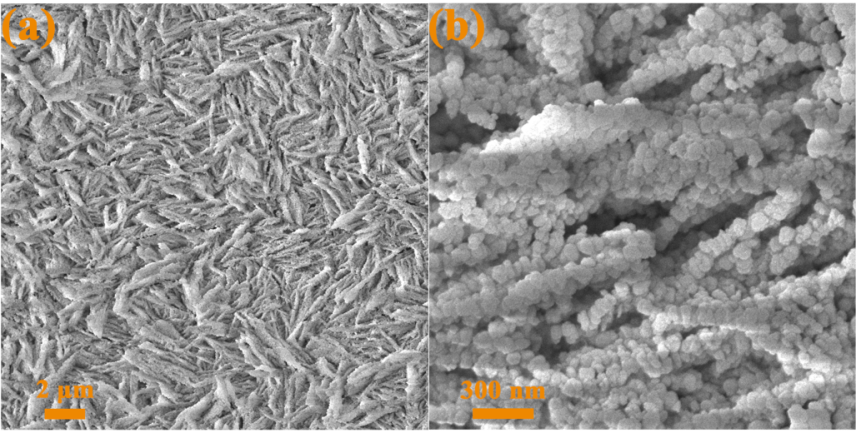


# Figure S8. SEM images of W-NiFe/NF at different magnifications. Scale bars are (a) 2 μm and (b) 300 nm.


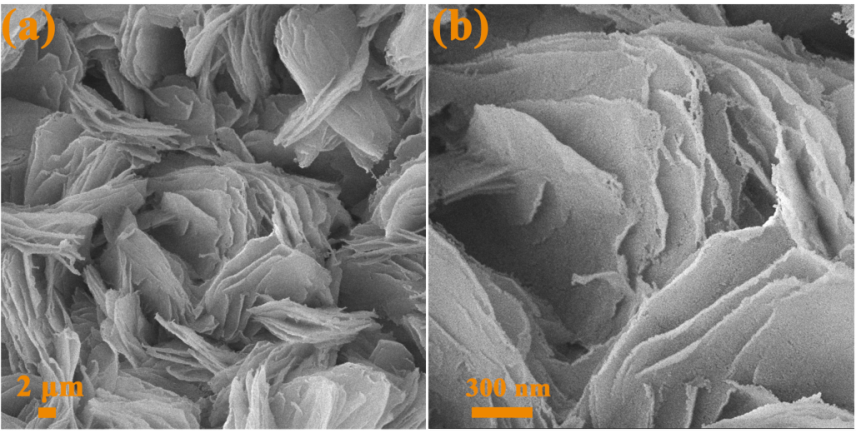


# Figure S9. SEM images of NiFeP/NF at different magnifications. Scale bars are (a) 2 μm and (b) 300 nm.


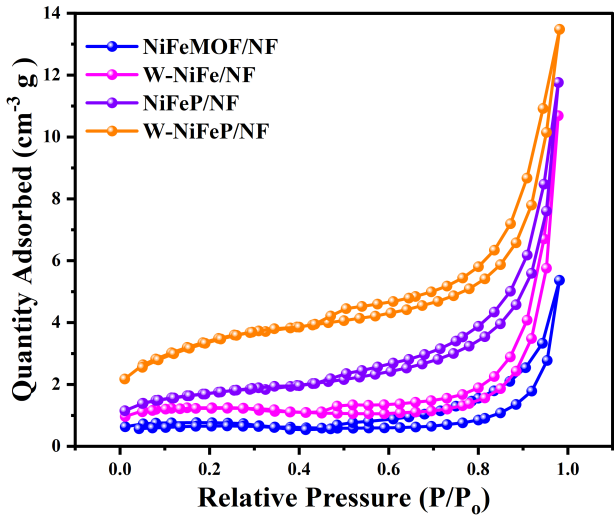


# Figure S10. N_2_ adsorption–desorption isotherms of NiFeMOF/NF, W-NiFe/NF, NiFeP/NF, and W-NiFeP/NF.


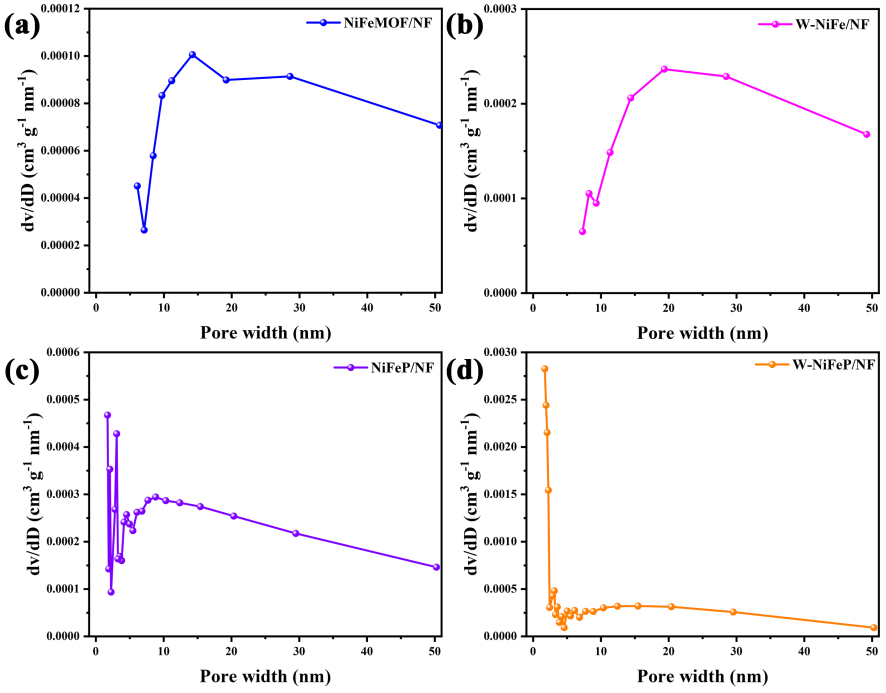


# Figure S11. Pore-size distribution plots of (a) NiFeMOF/NF, (b) W-NiFe/NF, (c) NiFeP/NF, and (d) W-NiFeP/NF.


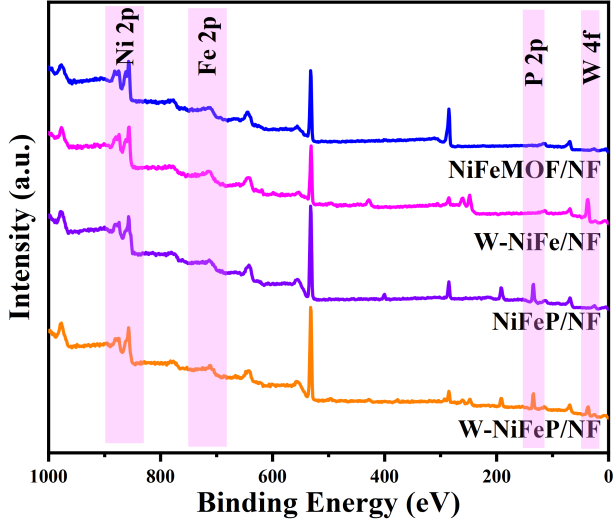


# Figure S12. XPS survey spectra of NiFeMOF/NF, W-NiFe/NF, NiFeP/NF, and W-NiFeP/NF.


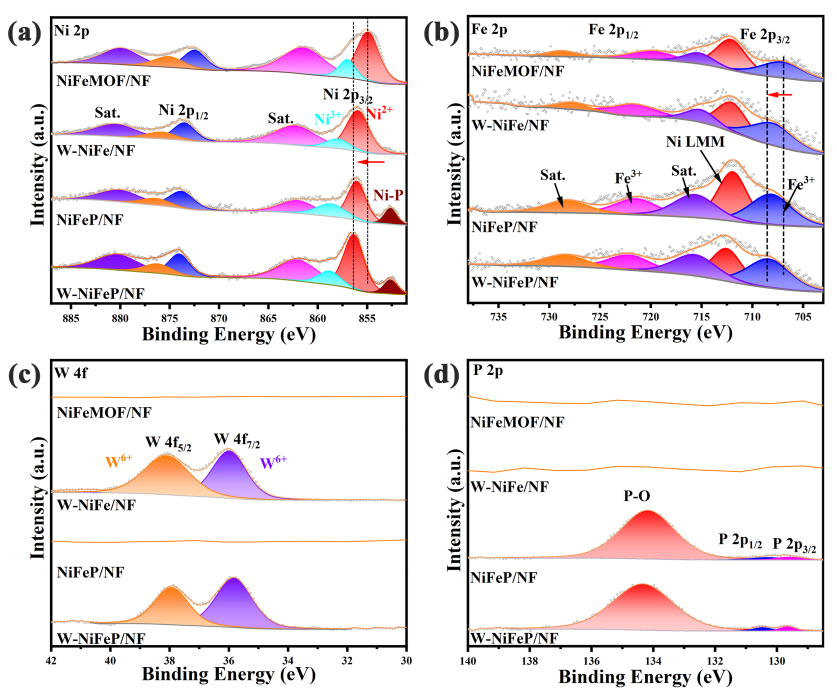


# Figure S13. High-resolution XPS spectra of the (a) Ni 2p, (b) Fe 2p, (c) W 4f and (d) P 2p electrons of NiFeMOF/NF, W-NiFe/NF, NiFeP/NF, and W-NiFeP/NF.


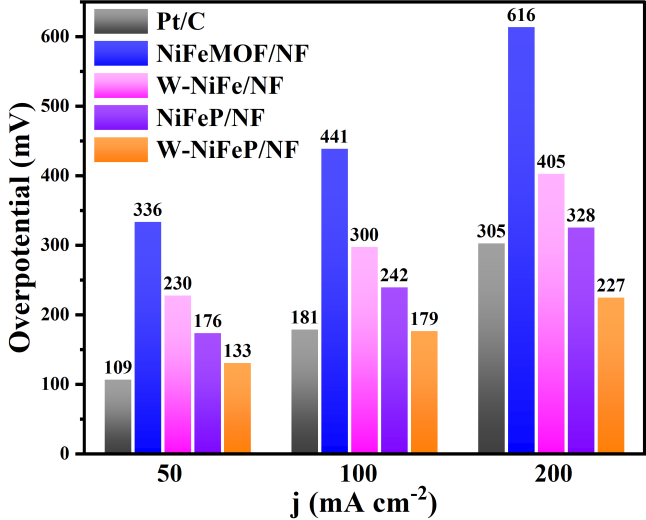


# Figure S14. HER overpotentials of NiFeMOF/NF, W-NiFe/NF, NiFeP/NF, W-NiFeP/NF, and Pt/C at different current densities in 1 M KOH.


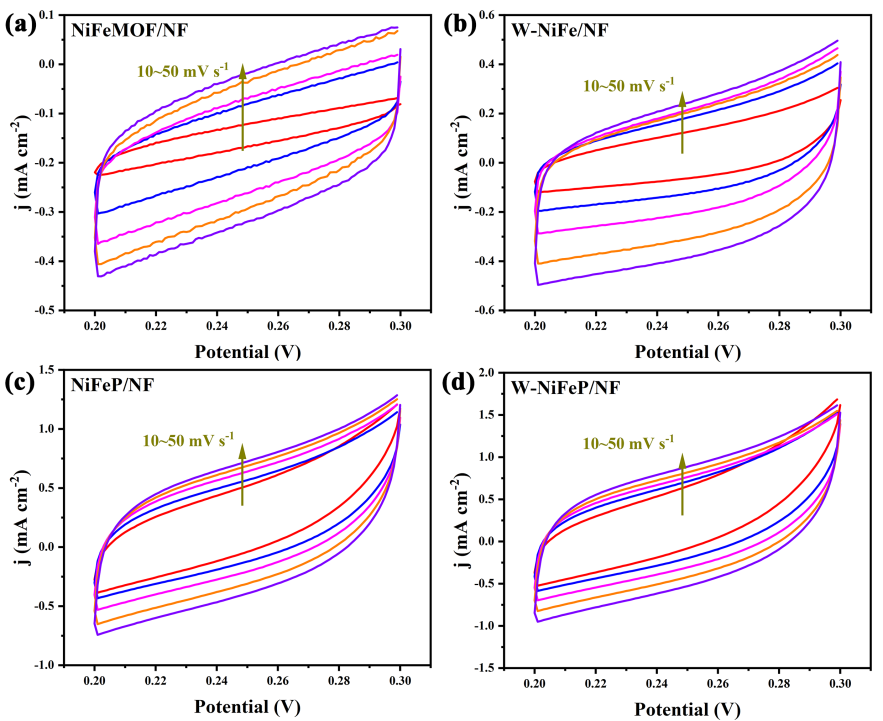


# **Figure S15.** CV curves of (a) NiFeMOF/NF, (b) W-NiFe/NF, (c) NiFeP/NF, and (d) W-NiFeP/NF in the non-faradic potential region (0.2~0.3 V vs RHE) at various scan rates (10~50 mV s^-1^).


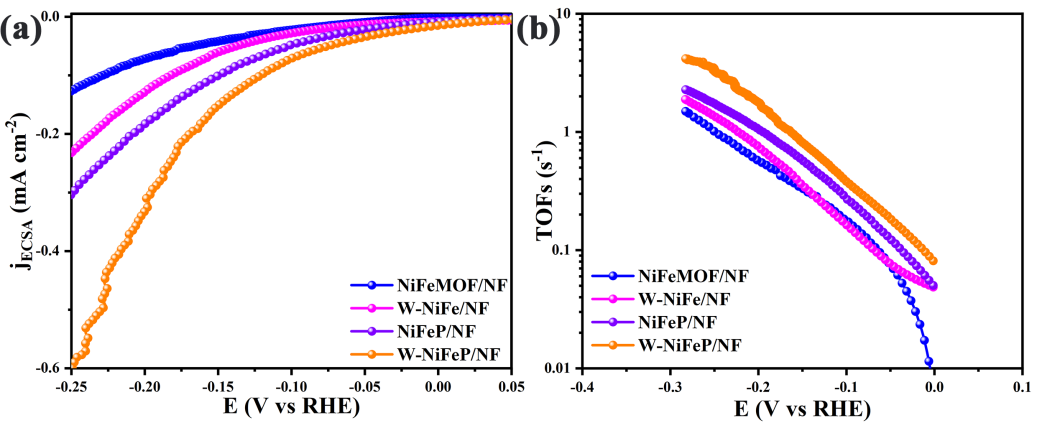


# Figure S16. (a) ECSA-normalized polarization curves and (b) TOFs of NiFeMOF/NF, W-NiFe/NF, NiFeP/NF, and W-NiFeP/NF for HER.


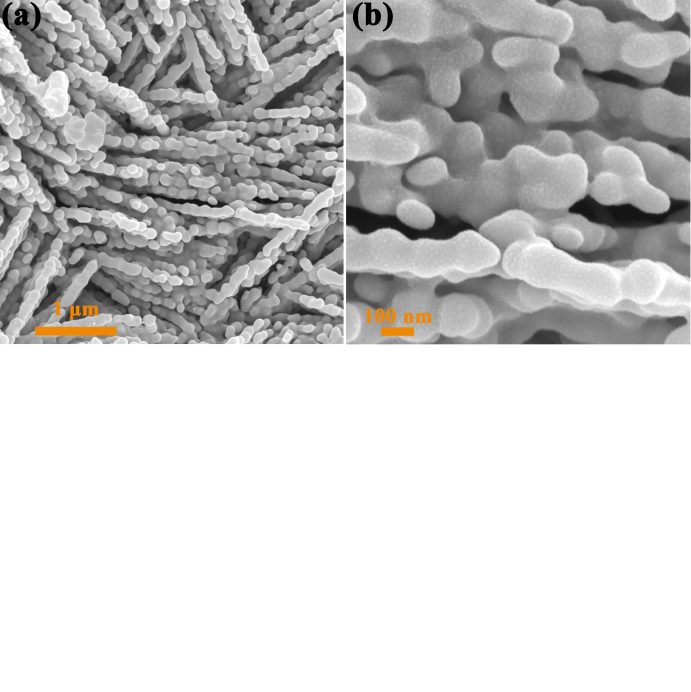


# Figure S17. SEM images of W-NiFeP/NF after HER test at different magnifications. Scale bars are (a) 1 μm and (b) 100 nm.


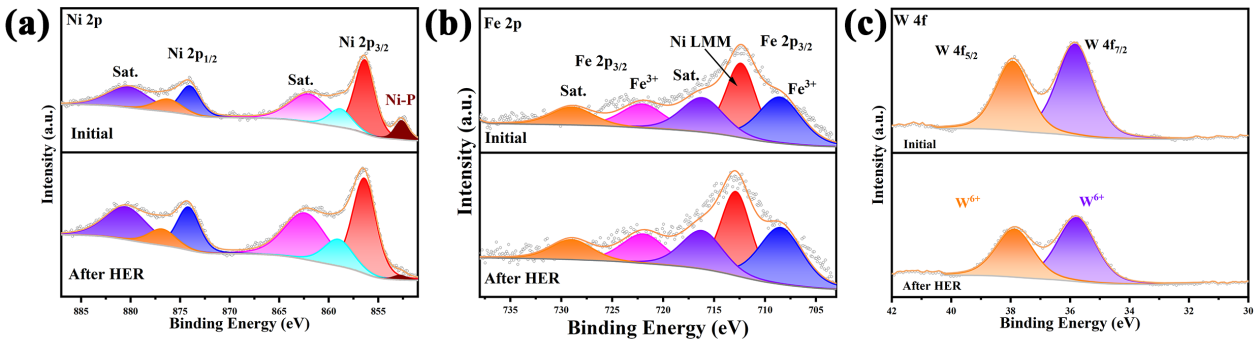


# Figure S18. High-resolution scans of the (a) Ni 2p, (b) Fe 2p and (c) W 4f electrons of W-NiFeP/NF after HER tests.


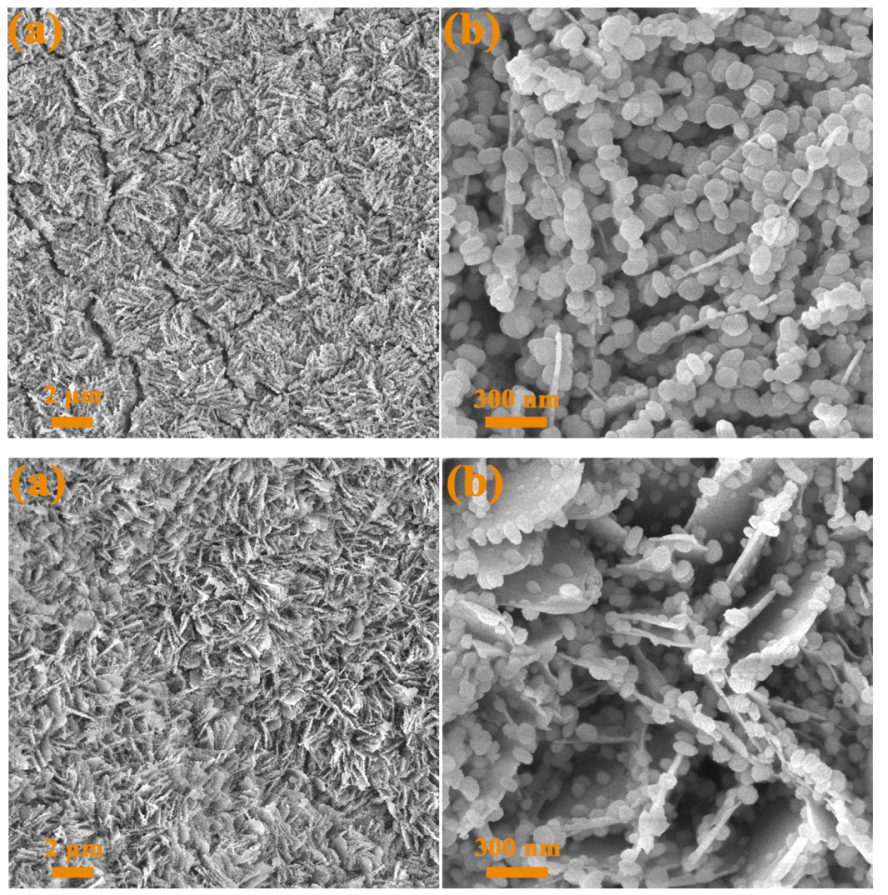


# Figure S19. SEM images of W_10_-NiFe/NF at different magnifications. Scale bars are (a) 2 μm and (b) 300 nm.


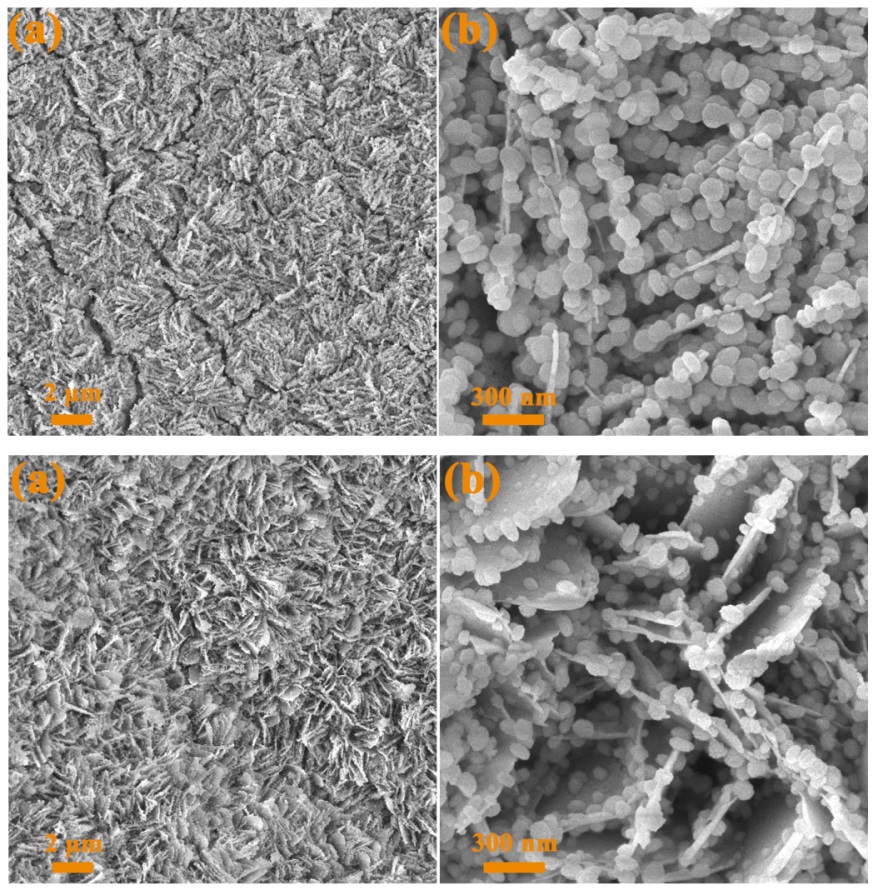


# Figure S20. SEM images of W_15_-NiFe/NF at different magnifications. Scale bars are (a) 2 μm and (b) 300 nm.


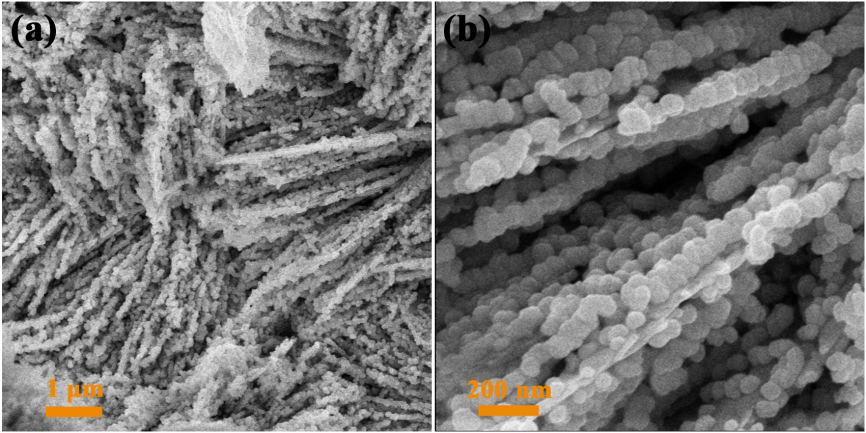


# Figure S21. SEM images of W_10_-NiFeP/NF at different magnifications. Scale bars are (a) 1 μm and (b) 200 nm.


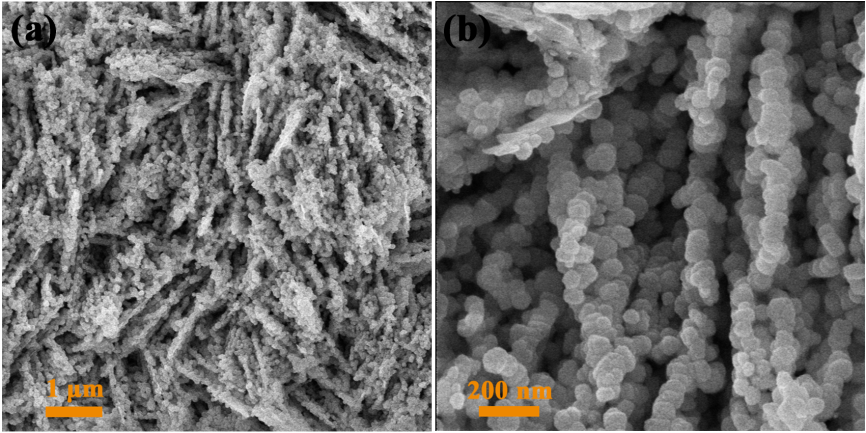


# Figure S22. SEM images of W_15_- NiFeP/NF at different magnifications. Scale bars are (a) 1 μm and (b) 200 nm.


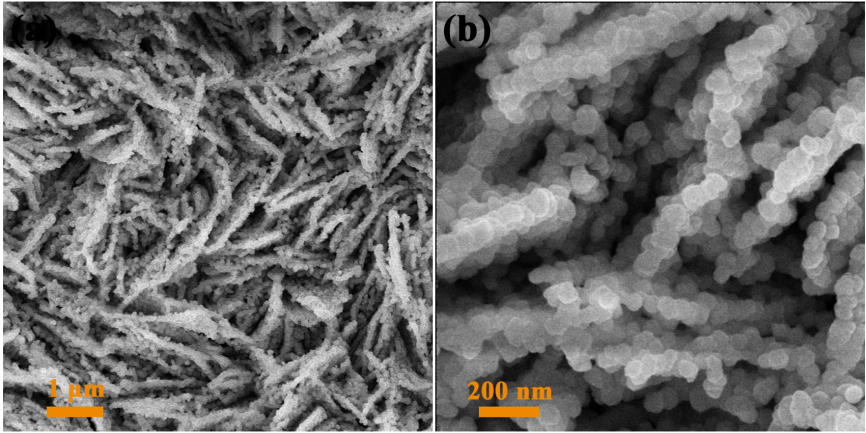


# Figure S23. SEM images of W_25_- NiFeP/NF at different magnifications. Scale bars are (a) 1 μm and (b) 200 nm.


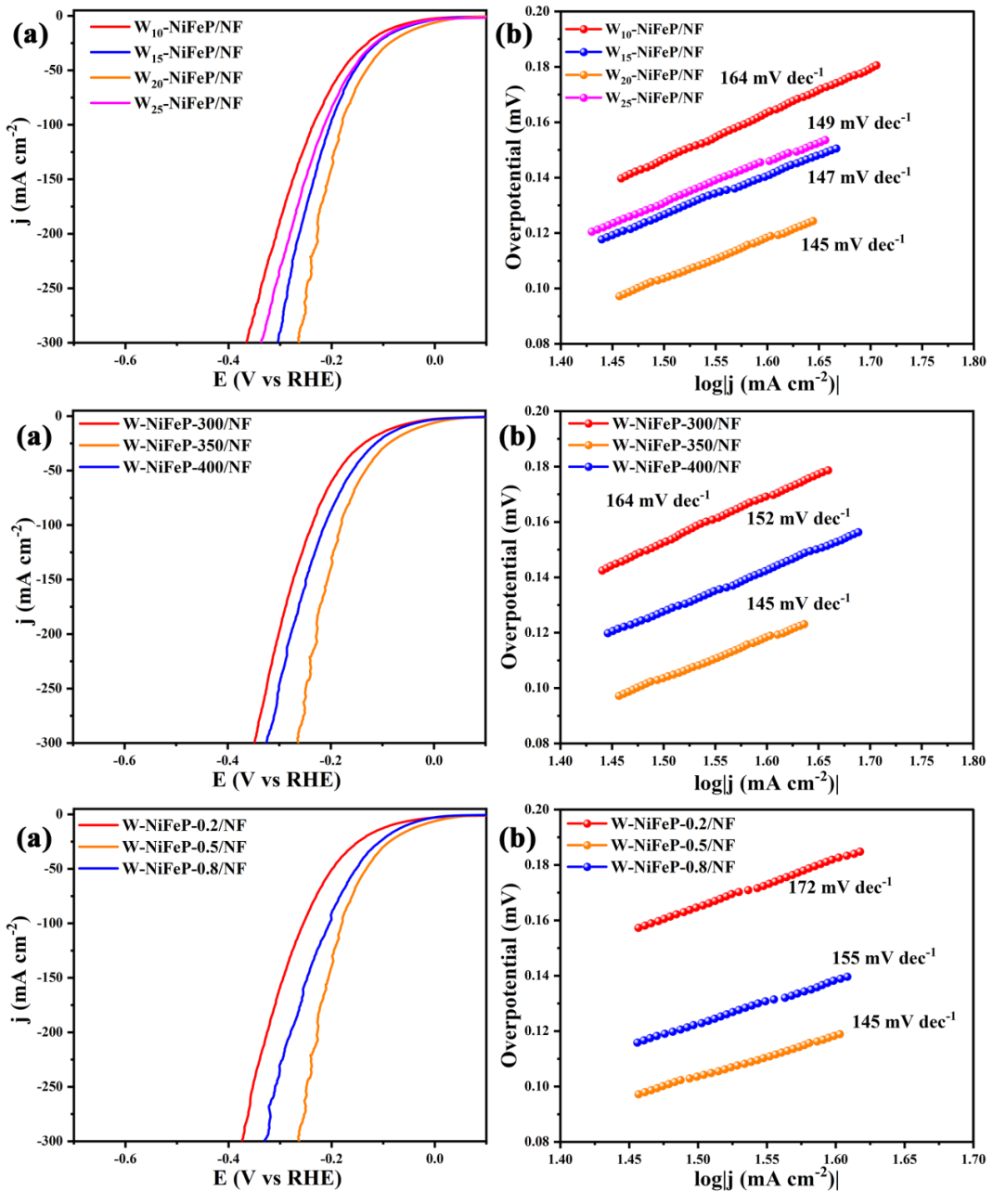


# Figure S24. (a) HER polarization curves and (b) Tafel plots of W-NiFeP/NF doped with different amounts of W.


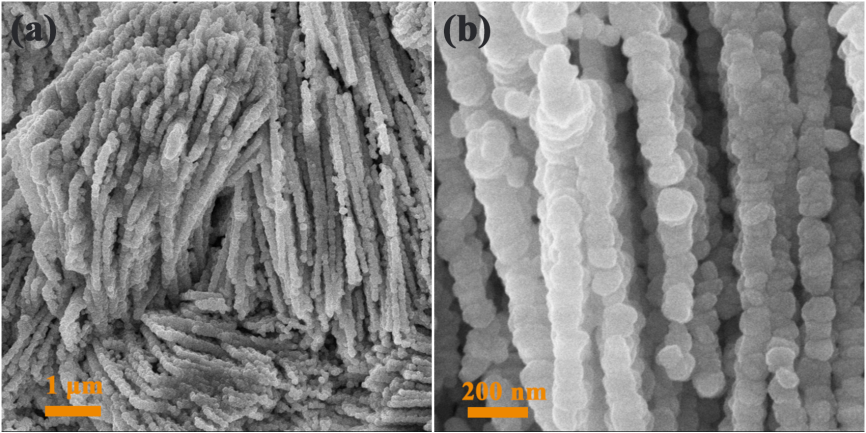


# Figure S25. SEM images of W-NiFeP-300/NF at different magnifications. Scale bars are (a) 1 μm and (b) 200 nm.


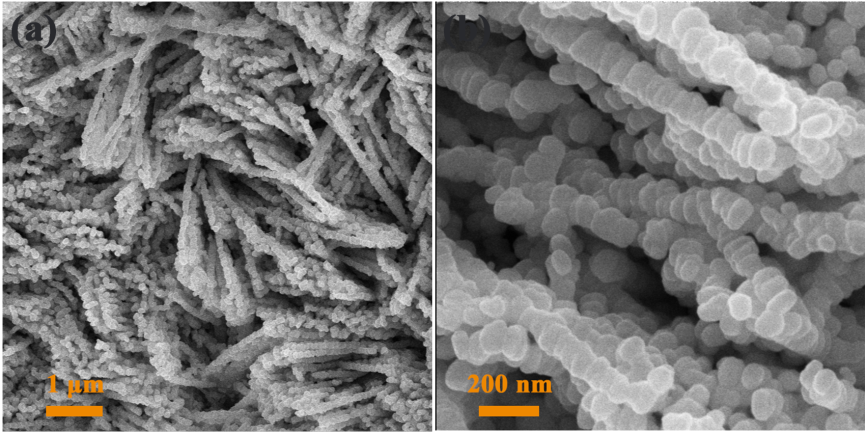


# Figure S26. SEM images of W-NiFeP-400/NF at different magnifications. Scale bars are (a) 1 μm and (b) 200 nm.


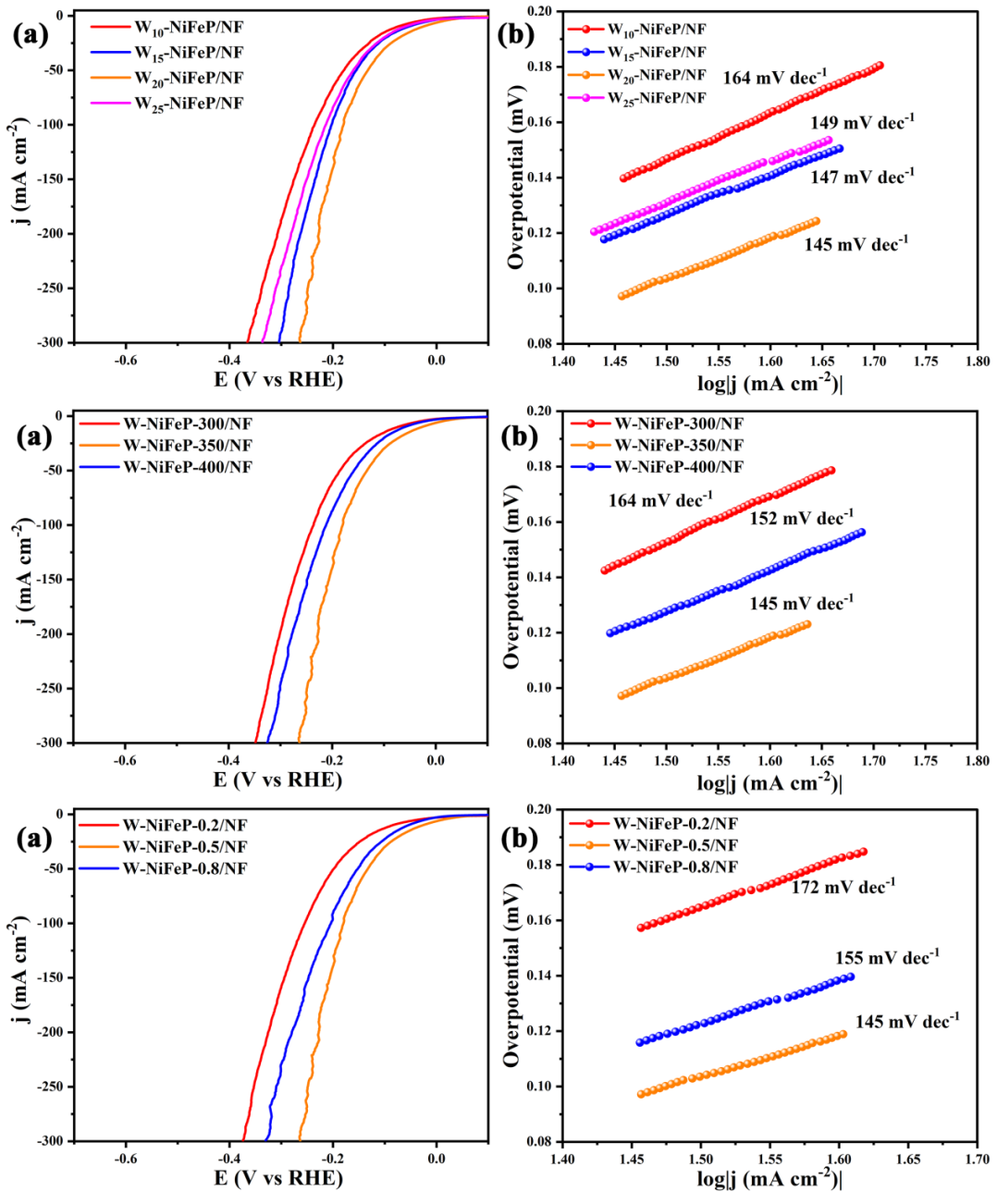


# Figure S27. (a) HER polarization curves and (b) Tafel plots of W-NiFeP/NF prepared at different phosphorylation temperatures.


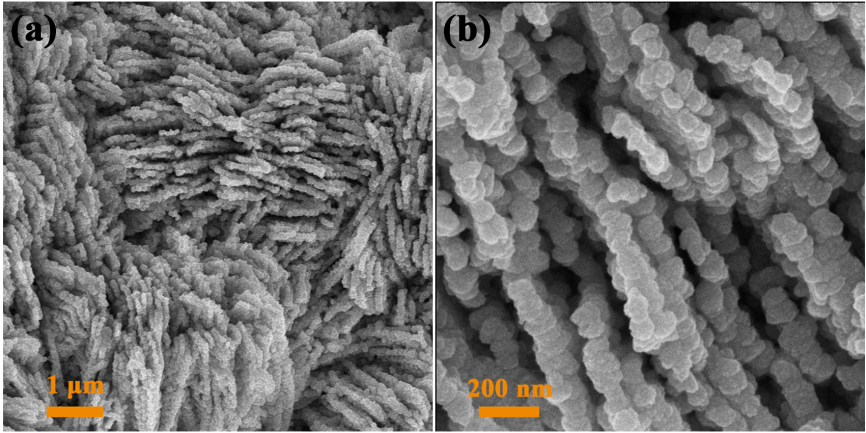


# Figure S28. SEM images of W-NiFeP-0.2/NF at different magnifications. Scale bars are (a) 1 μm and (b) 200 nm.


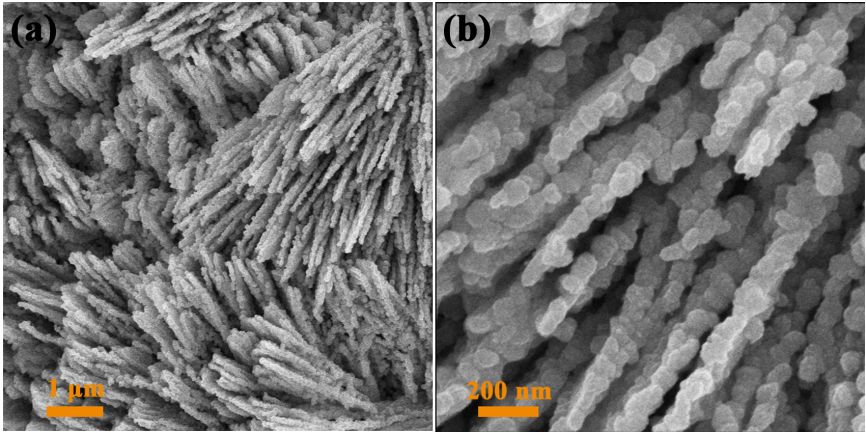


# Figure S29. SEM images of W-NiFeP-0.8/NF at different magnifications. Scale bars are (a) 1 μm and (b) 200 nm.


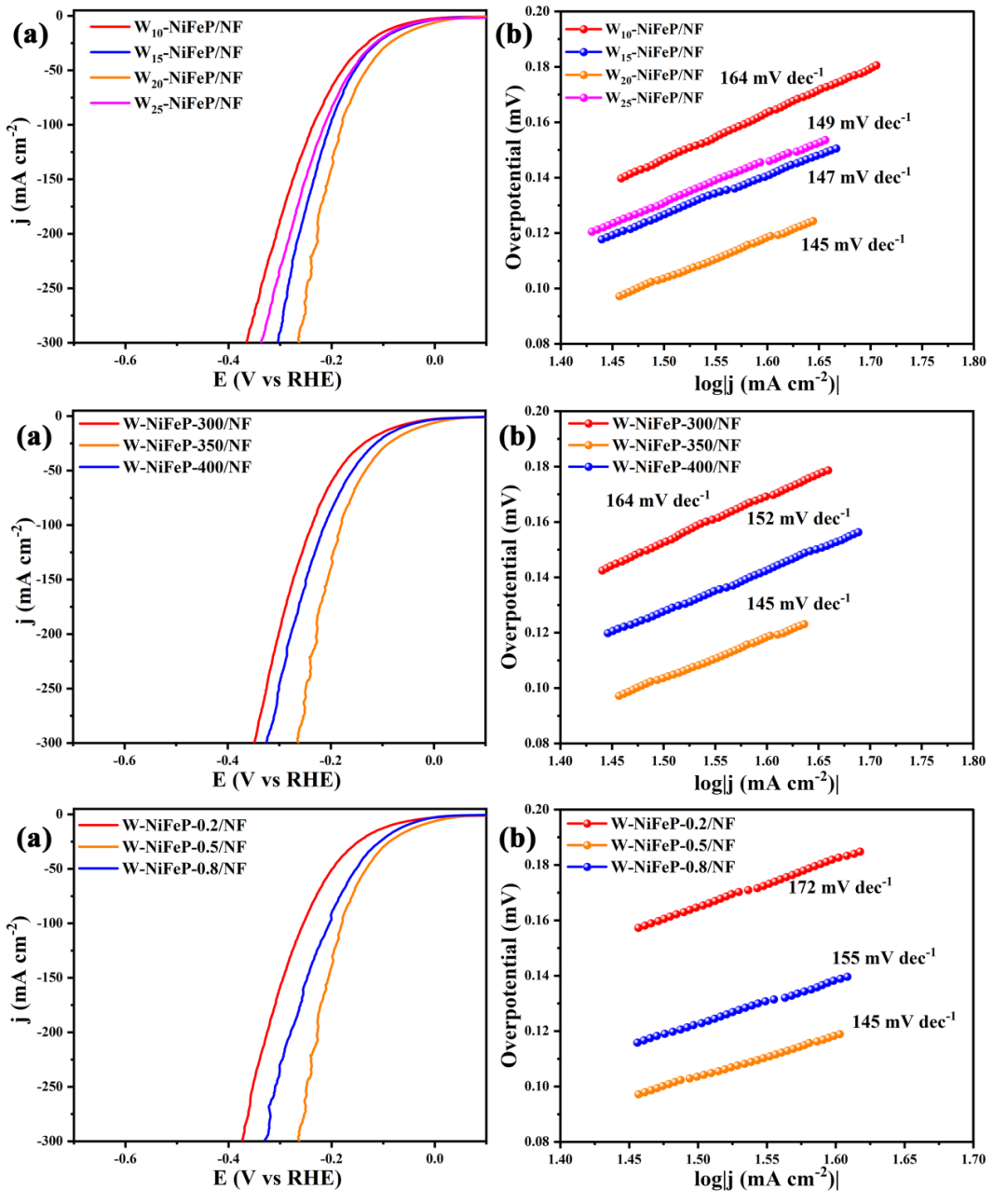


# Figure S30. (a) HER polarization curves and (b) Tafel plots of W-NiFeP/NF prepared with different amounts of sodium hydrogen hypophosphite.


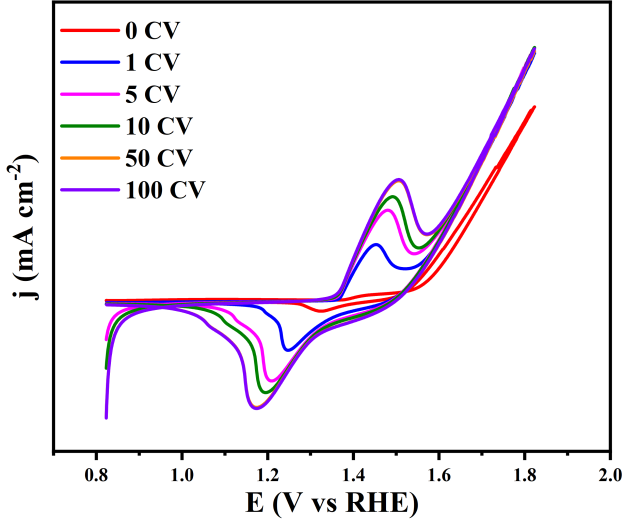


# Figure S31. Electrochemical activation of W-NiFeP/NF after varied CV cycles.


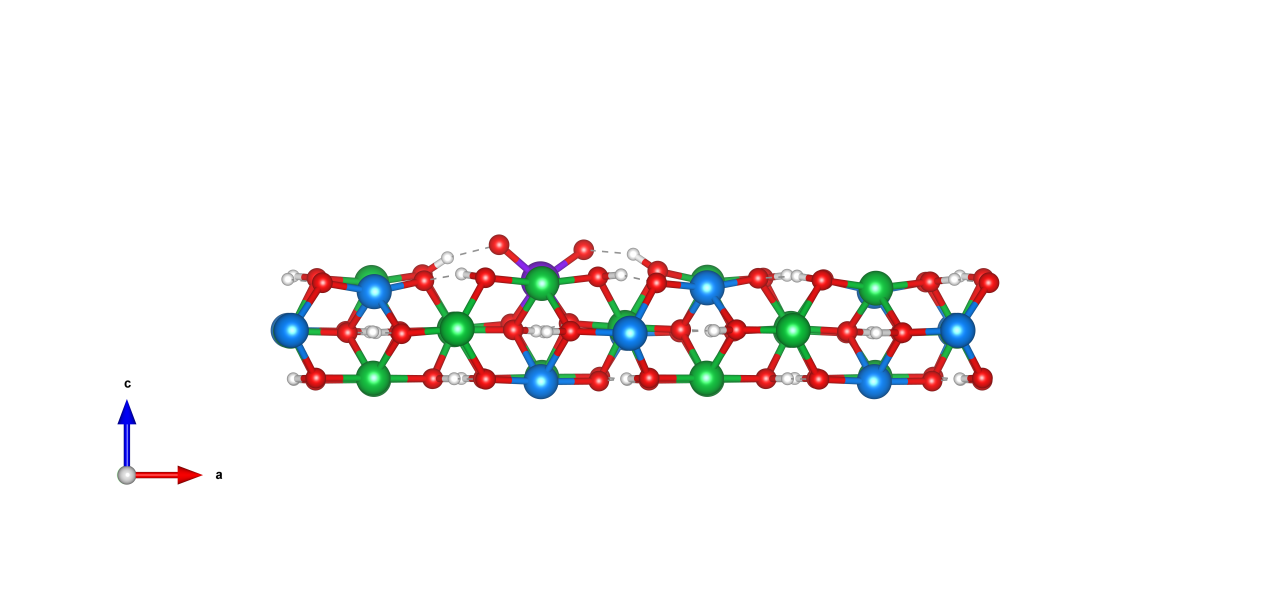


# **Figure S32.** Structural model of W-NiFeOOH.


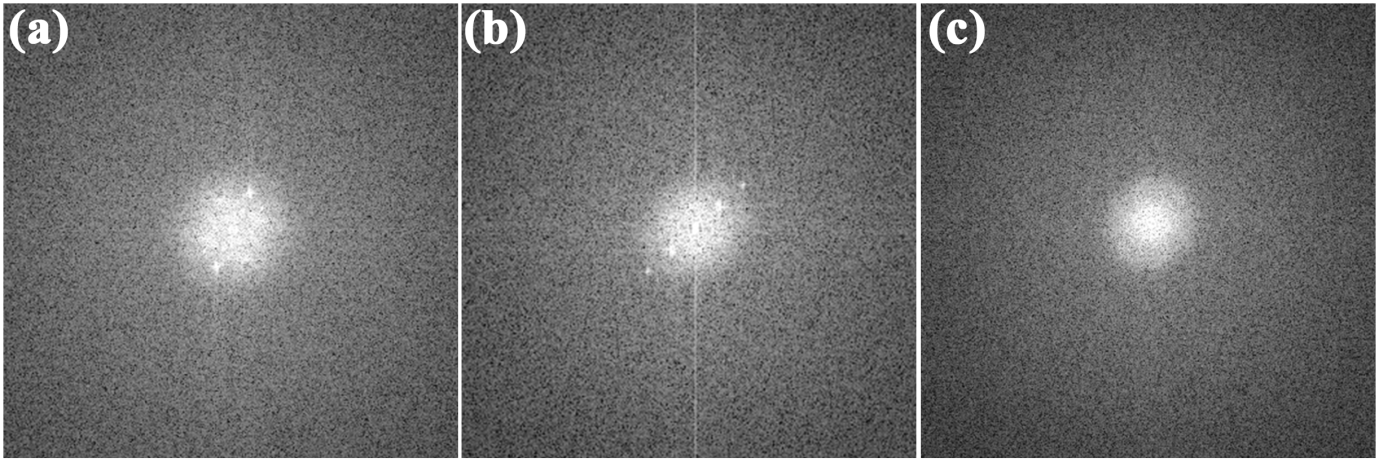


# **Figure S33.** FFT images of (a) area g1, (b) area g2, and (c) activated layer in Figure 4f. No clear diffraction patterns can be observed in Figure S33c, confirming the presence of non-crystalline structure.


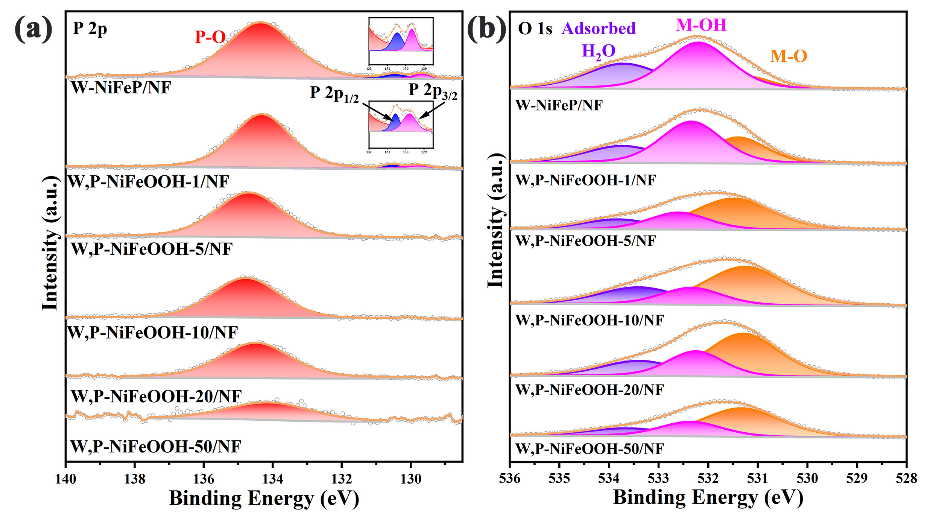


# Figure S34. High-resolution XPS scans of the (a) P 2p and (b) O 1s electron of W-NiFeP/NF after various CV cycles of electrochemical activation in 1 M KOH. W,P-NiFeOOH-1/NF represents the electrochemical activation of W-NiFeP/NF after 1 CV cycle, and so on.

**
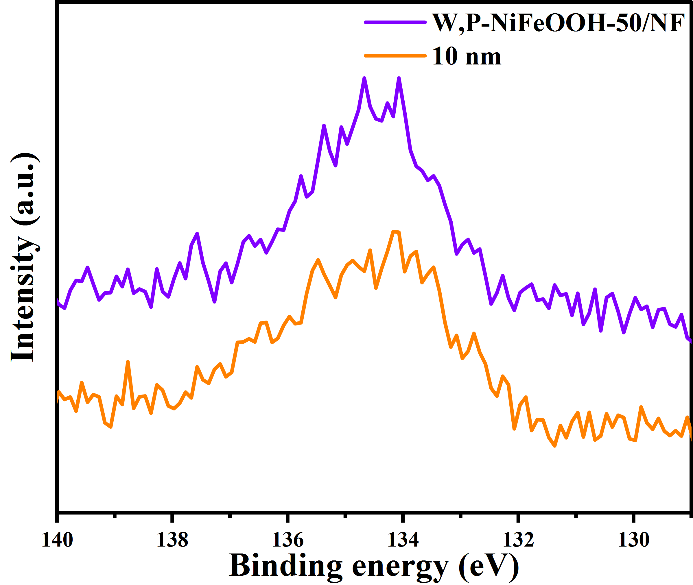
**

# Figure S35. High-resolution depth-profiling XPS spectra of P 2p for W,P-NiFeOOH-50/NF.


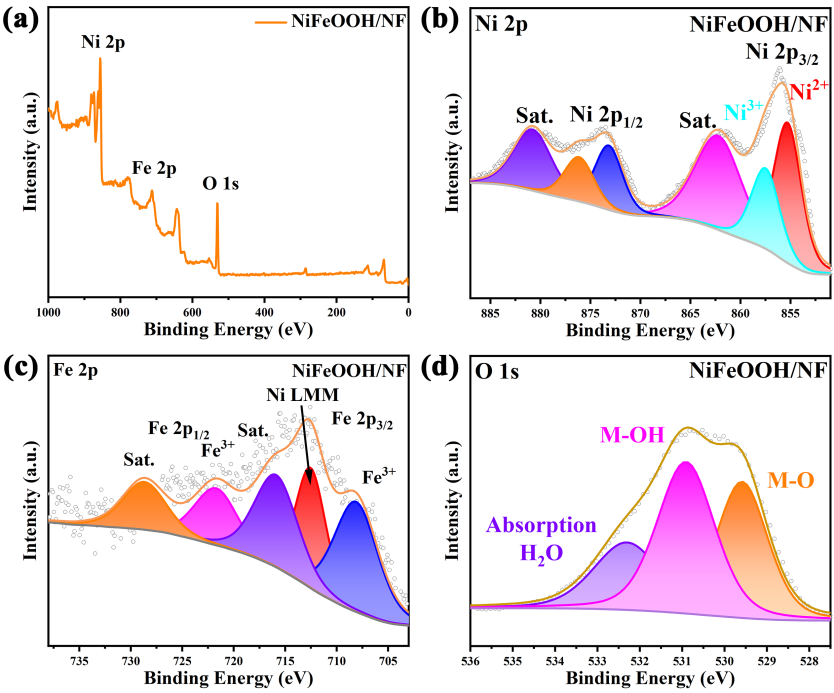


# Figure S36. XPS spectra of NiFeOOH/NF: (a) survey spectra and high-resolution scans of the (b) Ni 2p, (c) Fe 2p, and (d) O 1s electrons.

**
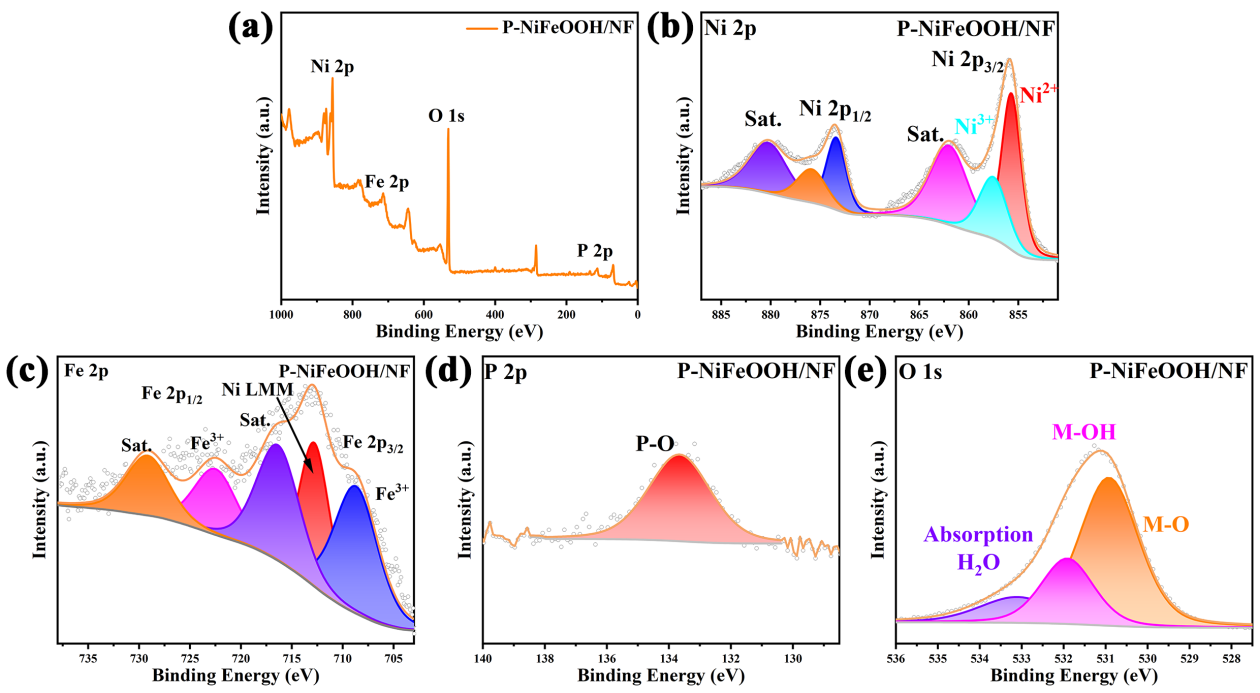
**

# Figure S37. XPS spectra of W-NiFeOOH/NF: (a) survey spectra and high-resolution scans of the (b) Ni 2p, (c) Fe 2p, (d) W 4f, and (e) O 1s electrons.


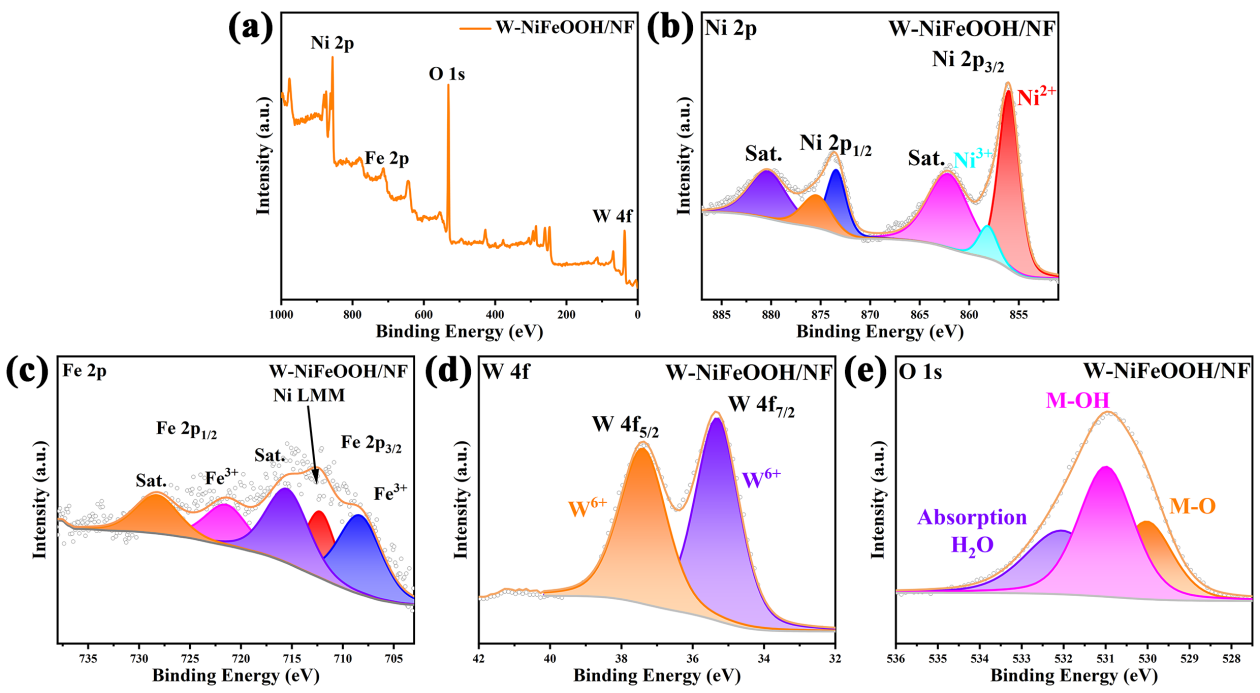


# Figure S38. XPS spectra of P-NiFeOOH/NF: (a) Survey spectra and high-resolution spectra for (b) Ni 2p, (c) Fe 2p, (d) P 2p, and (e) O 1s


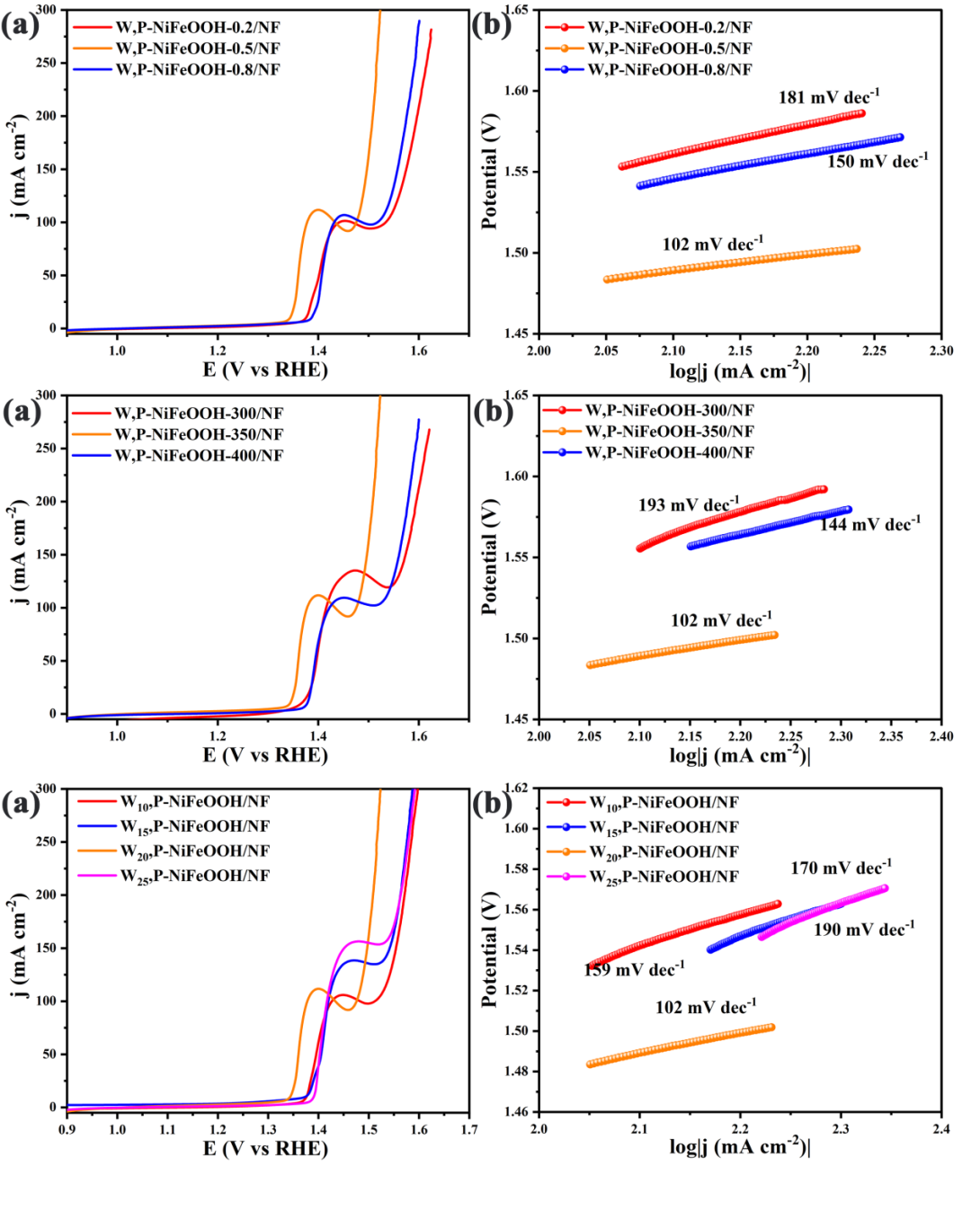


# Figure S39. (a) OER polarization curves and (b) Tafel plots of W,P-NiFeOOH/NF doped with different amounts of W.


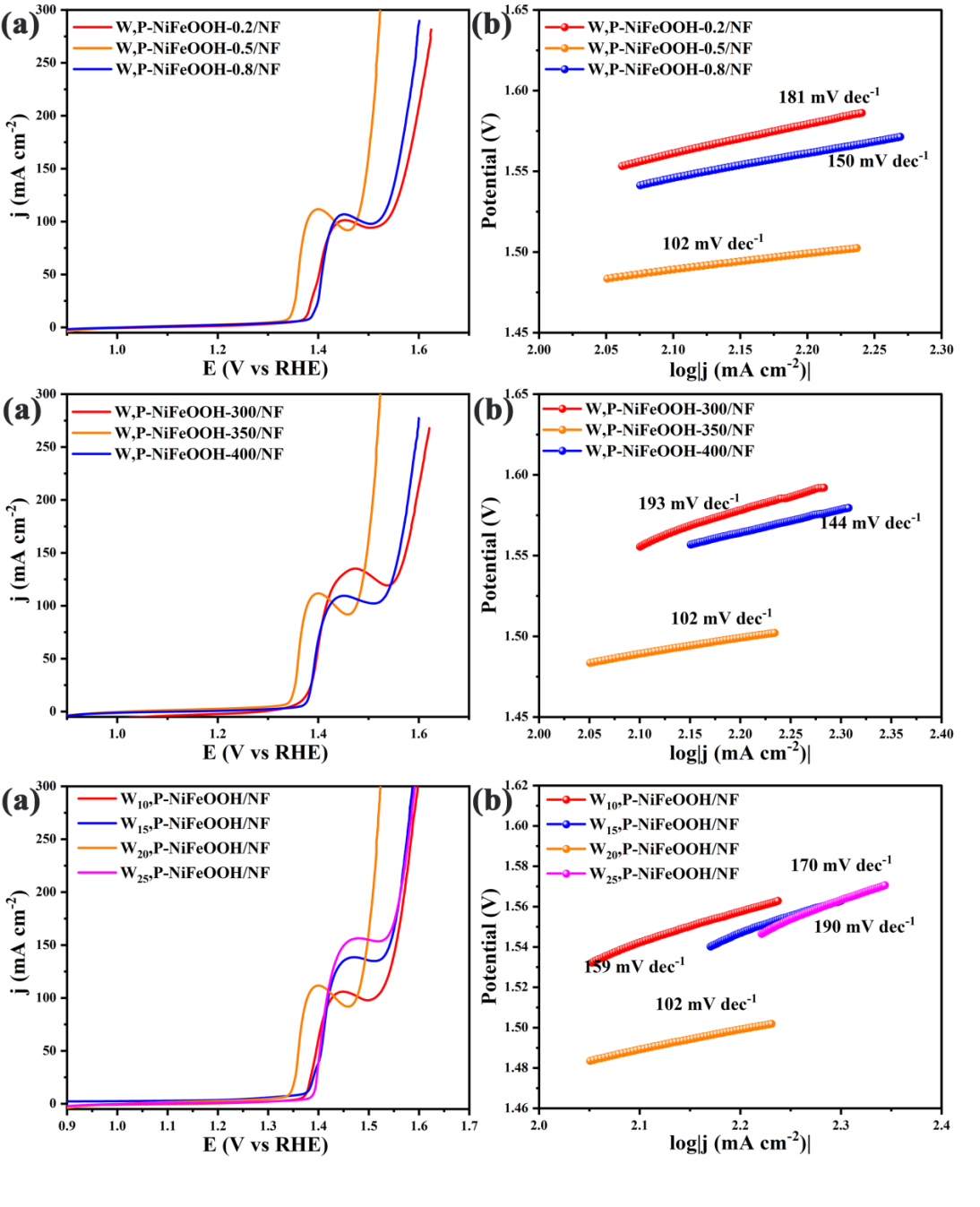


# Figure S40. (a) OER polarization curves and (b) Tafel plots of W,P-NiFeOOH/NF prepared at different phosphorylation temperatures.


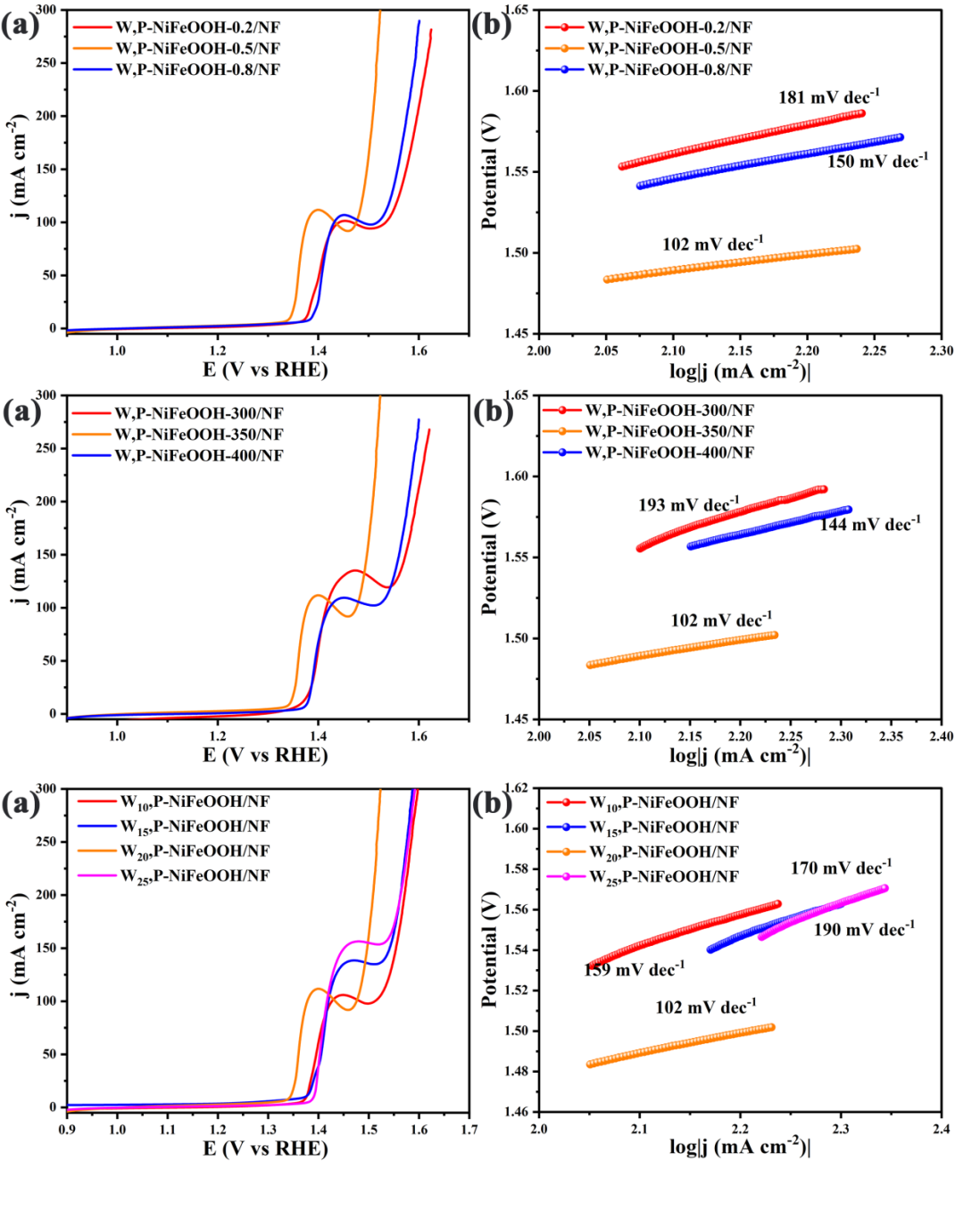


# Figure S41. (a) OER polarization curves and (b) Tafel plots of W,P-NiFeOOH/NF prepared with different amounts of sodium hydrogen hypophosphite. W,P-NiFeOOH/NF exhibits the best OER performance when the W doping amount, sodium hydrogen hypophosphite dosage and phosphorization temperature are 20 mg, 0.5 g and 350 °C, respectively (Figure S39-S41).


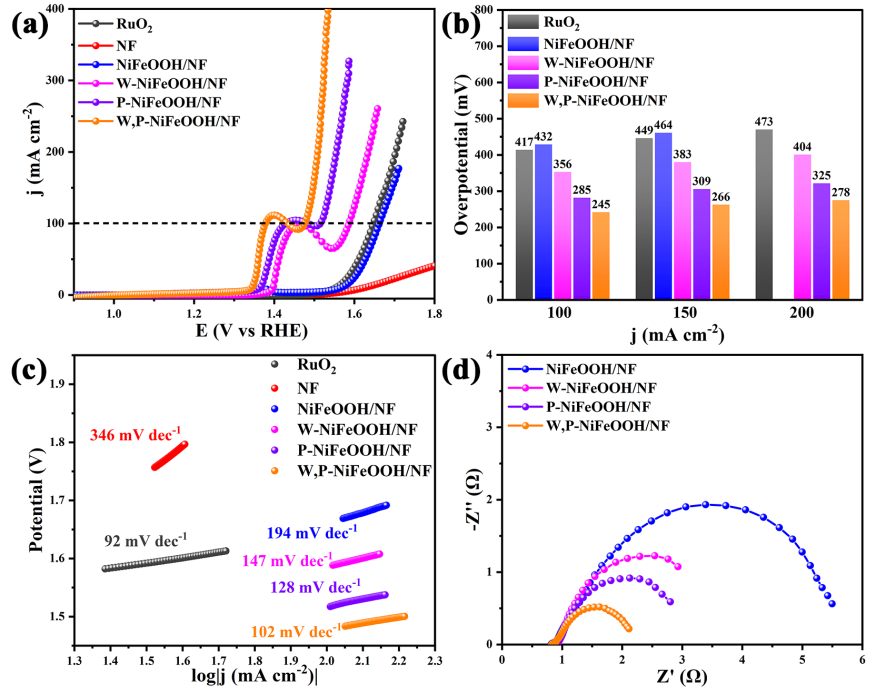


# Figure S42. (a) OER polarization curves, (b) overpotentials at different current densities in 1 M KOH, (c) Tafel plots and (d) Nyquist plots of NiFeOOH/NF, W-NiFeOOH/NF, P-NiFeOOH/NF, W,P-NiFeOOH/NF, and RuO_2_.

Comparison samples W-NiFeOOH/NF and P-NiFeOOH/NF were synthesized under the above conditions, and compared with the commercial RuO_2_ and NF, as shown in **Figure S42a-b**. Specifically, the overpotentials required for W,P-NiFeOOH/NF to reach the current densities of 100, 150, and 200 mA cm^-2^ were only 245, 266, and 278 mV, which were even lower than those for RuO_2_ (417, 449, and 473 mV). Furthermore, for NiFeOOH/NF, W-NiFeOOH and P-NiFeOOH/NF, to achieve a current density of 100 mA cm^-2^ an overpotential of 432, 356 and 285 mV was needed, respectively, which were significantly higher than that (245 mV) of W, P-NiFeOOH/NF.

Moreover, compared with NF (346 mV dec^-1^), NiFeOOH/NF (194 mV dec^-1^), W-NiFeOOH/NF (147 mV dec^-1^), and P-NiFeOOH/NF (128 mV dec^-1^), W, P-NiFeOOH/NF showed the smallest Tafel slope of 102 mV dec^-1^, suggesting most facile OER reaction kinetics among the series (**Figure S42c**). W,P-NiFeOOH/NF can be seen to possess an excellent electrocatalytic activity at high current density, and W and P doping played a significant role. This was mainly due to the fact that after W,P doping and electrochemical activation, the surface of W,P-NifeOOH/NF was corroded and roughened into fine nanosheets, which increased the accessibility of the electrocatalytic active sites and improves the mass transfer. In addition, the doping of W and P changed the electronic environment of the Ni and Fe sites for enhanced OER performance. From the Nyquist plot in **Figure S42d**, W,P-NiFeOOH/NF can be seen to possess the smallest R_ct_, along with the greatest ECSA and C_dl_ (**Figure S43**). In addition, W,P-NiFeOOH/NF exhibited higher TOF and ECSA-normalized current densities at the same potentials, implying that in situ W and P doping enhanced the intrinsic activity (**Figure S44**).


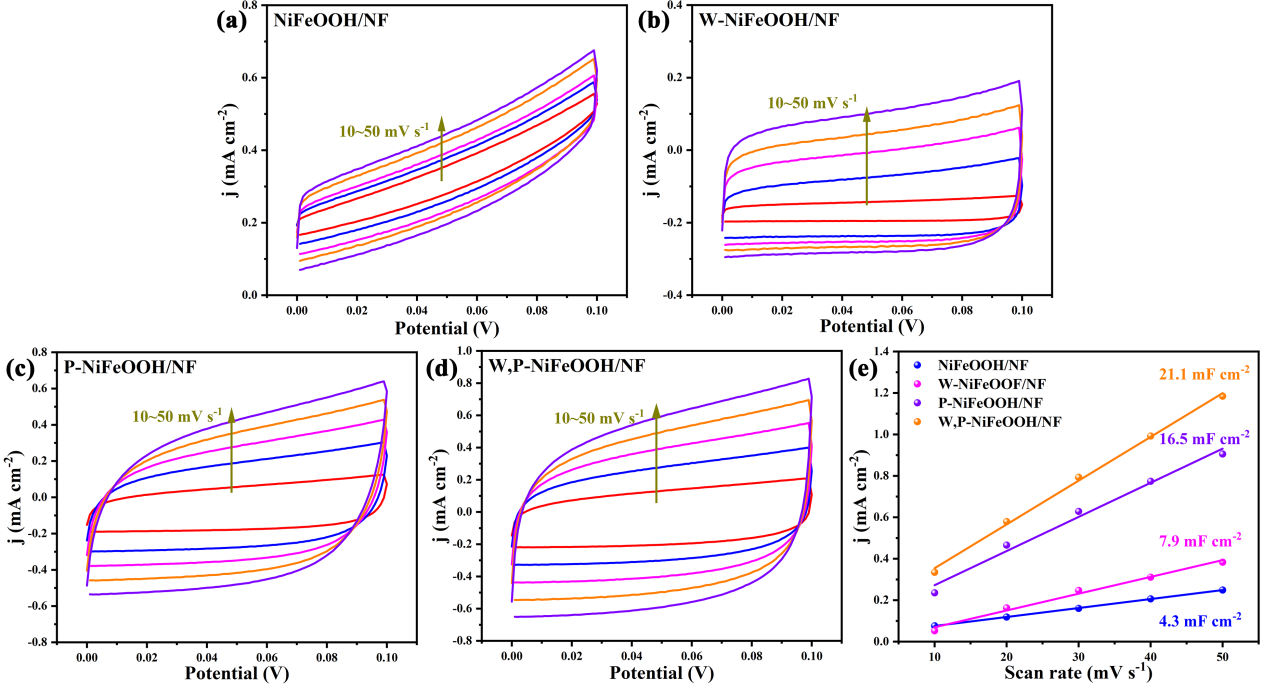


# Figure S43. CV curves of (a) NiFeOOH/NF, (b) W-NiFeOOH/NF, (c) P-NiFeOOH/NF, and (d) W,P-NiFeOOH/NF in the non-faradic potential region at various scan rates (10~50 mV s^-1^). (e) The corresponding variation of the double-layer charging current with potential scan rate, from which C_dl_ is evaluated.


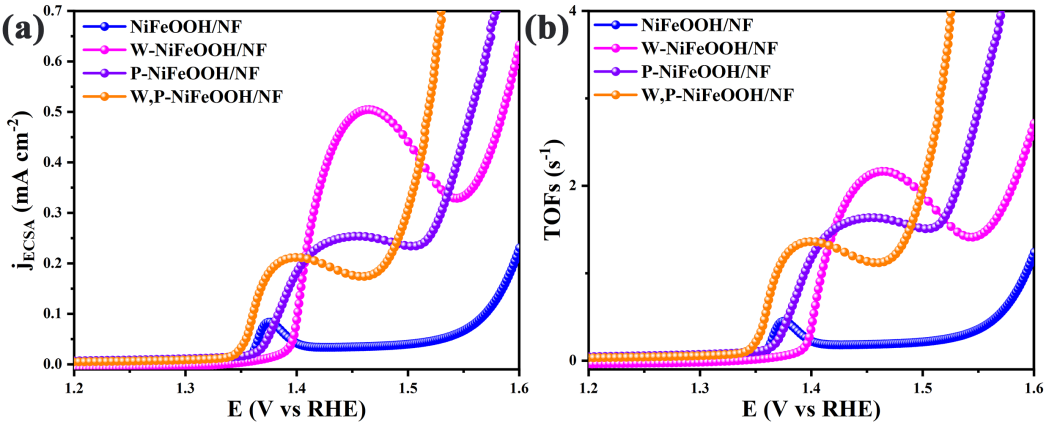


# Figure S44. (a) ECSA-normalized OER polarization curves and (b) TOFs of NiFeOOH/NF, W-NiFeOOH/NF, P-NiFeOOH/NF, and W,P-NiFeOOH/NF.


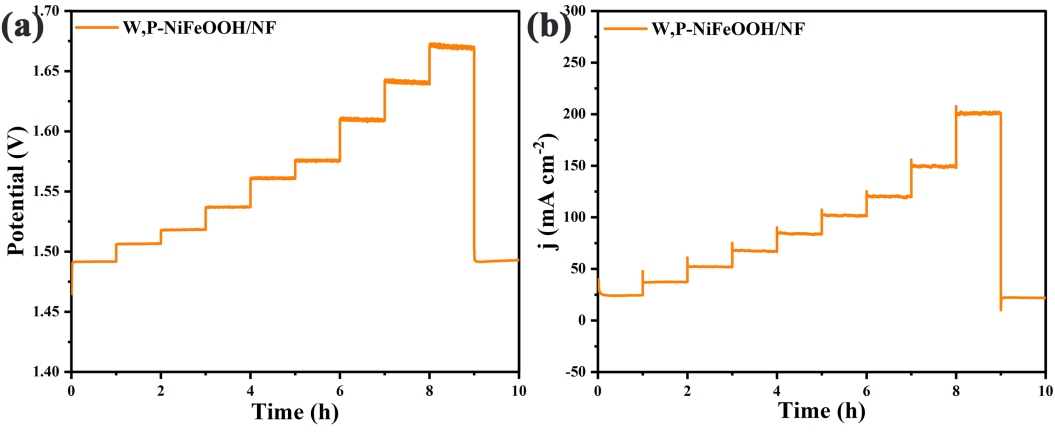


# Figure S45. (a) Multiple current density and (b) multiple potential tests of W,P-NiFeOOH/NF.

The stability of W,P-NiFeOOH/NF was also evaluated by multiple current and voltage tests. As shown in **Figure S45**, W,P-NiFeOOH/NF exhibited essentially no change of the profiles under different voltage and current tests for 10 h.


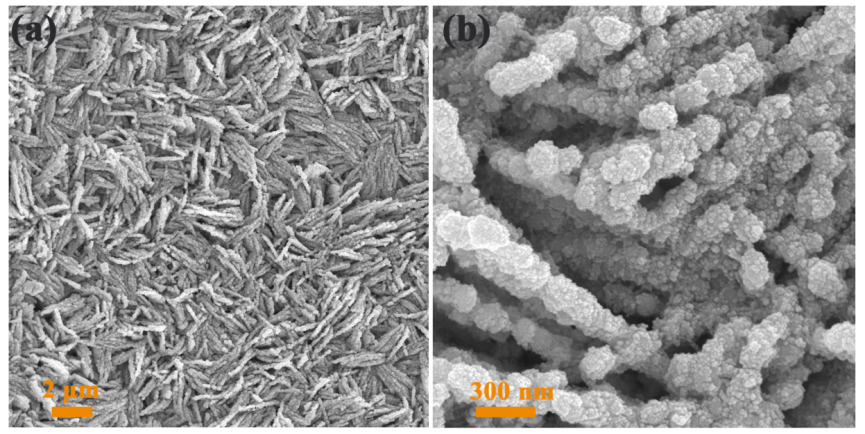


# Figure S46. SEM images of W,P-NiFeOOH/NF after OER tests at different magnifications. Scale bar are (a) 2 μm and (b) 300 nm.


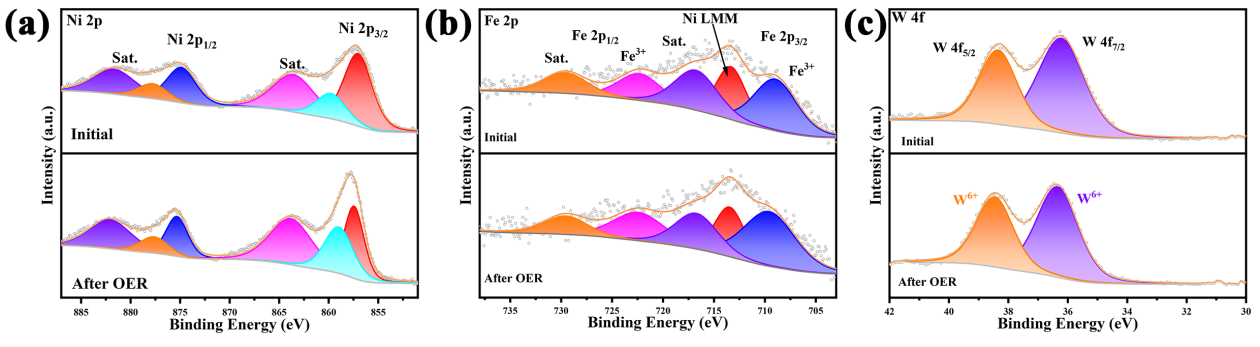


# Figure S47. High-resolution scans of the (a) Ni 2p, (b) Fe 2p and (c) W 4f electrons of W,P-NiFeOOH/NF after OER tests.


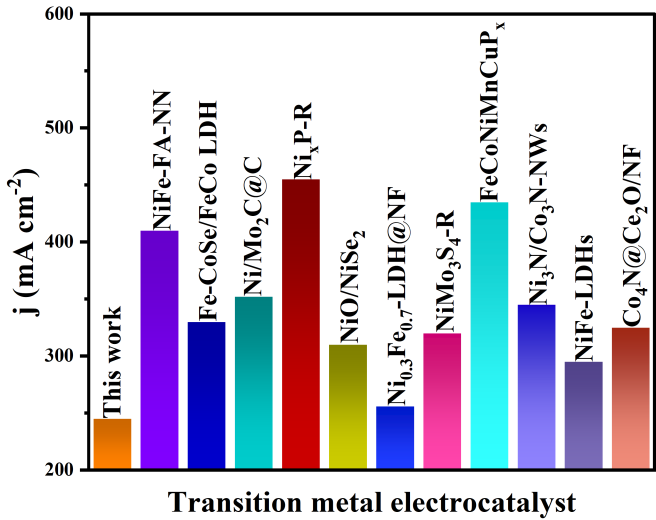


# Figure S48. The comparison of overpotentials at 100 mA cm^-2^ for W,P-NiFeOOH/NF with the other previously reported transition metal electrocatalysts.

**Figure S46-S47** show the morphology and chemical structure of W,P-NiFeOOH/NF after OER stability tests, which further confirmed the structural stability of the sample. Mechanistically, electrochemical activation and W and P doping modulated the electronic environments of the Ni and Fe sites, which enabled the W,P-NiFeOOH/NF to exhibit enhanced OER electrocatalytic activity as compared to those of relevant non-precious metal electrocatalysts reported recently in the literature (**Figure S48**).


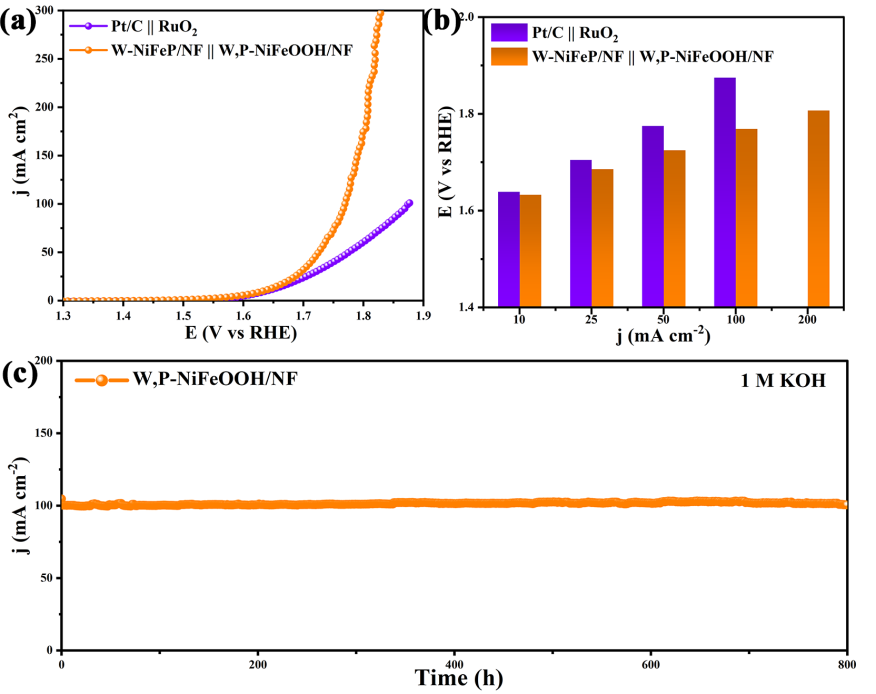


# Figure S49. (a) Polarization curves of overall water splitting for W-NiFeP/NF|| W,P-NiFeOOH/NF and Pt||RuO_2_ in 1 M KOH with a two-electrode system. (b) Comparison of cell voltages for W-NiFeP/NF|| W,P-NiFeOOH/NF and Pt||RuO_2_ at various current densities. (c) Chronoamperometric curves of W-NiFeP/NF|| W,P-NiFeOOH/NF at 1.79 V to deliver a current density of 100 mA cm^-2^ for 800 h.


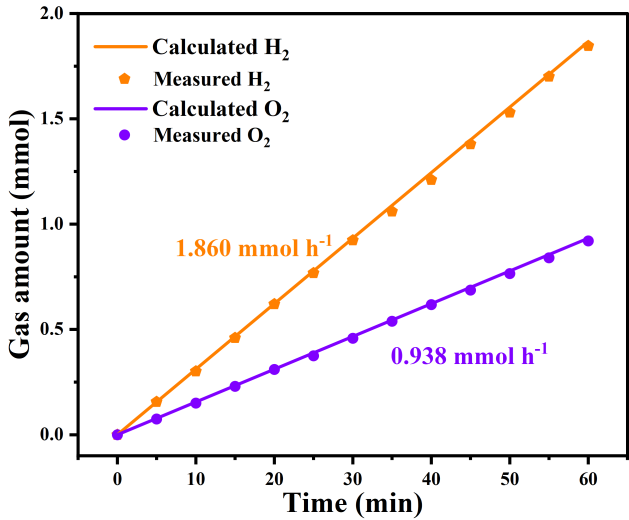


# Figure S50. The amounts of hydrogen and oxygen released during the water splitting experiment.

Considering the excellent HER performance of W-NiFeP/NF and the excellent OER performance of W,P-NiFeOOH/NF, a two-electrode electrolyzer was constructed with W-NiFeP/NF as the cathodic catalyst and W,P-NiFeOOH/NF as the anodic catalyst for overall water splitting. Impressively, W-NiFeP/NF||W,P-NiFeOOH/NF needed a cell voltage of 1.636, 1.686, 1.725, 1.789, and 1.807 V to produce a current density of 10, 25, 50, 100, and 200 mA cm^-2^, respectively, a performance better than that with commercial benchmarks of Pt/C||RuO_2_, and those of leading catalysts recently reported in the literature (**Figure S49a-b and Table S3**).

Consideration of stability was an important indicator for the long-term operation of an electrolyzer. At the potential of 1.79 V, the two-electrode electrolyzer system can be operated stably to deliver a current density of 100 mA cm^-2^ for 800 h without obvious fluctuations (**Figure S49c**), implying remarkable durability. In addition, the hydrogen and oxygen produced during the experiment were collected by the drainage method and compared with the theoretical values to investigate the Faraday efficiency. As shown in **Figure S50**, the ratio of the actual amount of hydrogen and oxygen produced is close to 2:1, consistent with the theoretical expectation. The actual amount of gas produced is also close to the theory as well, which means that the Faraday efficiency is close to 100%, confirming that no other reactions take place in the overall water splitting system except for OER and HER. The above results indicated that the NiFe-based MOF can be used as a substrate to obtain an optimized electronic structure, high intrinsic activity, abundant active sites, and good mass transfer characteristics through W cation exchange, phosphatization, and electrochemical in-situ activation, so that W-NiFeP/NF can be used as an electrocatalyst for the cathode and a precatalyst for the anode in the overall water splitting.


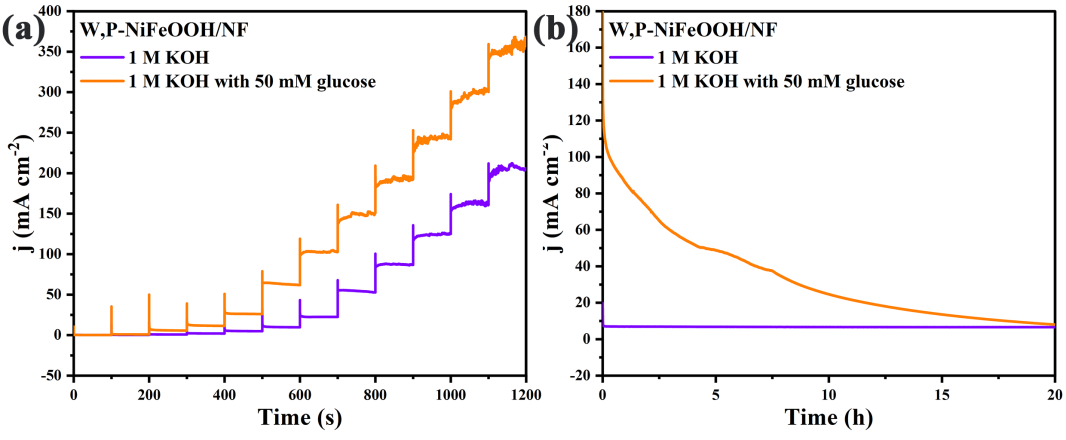


# Figure S51. (a) Multiple potential tests and (b) chronoamperometric curves (1.35 V) of W,P-NiFeOOH/NF in 1 M KOH with and without 50 mM glucose.


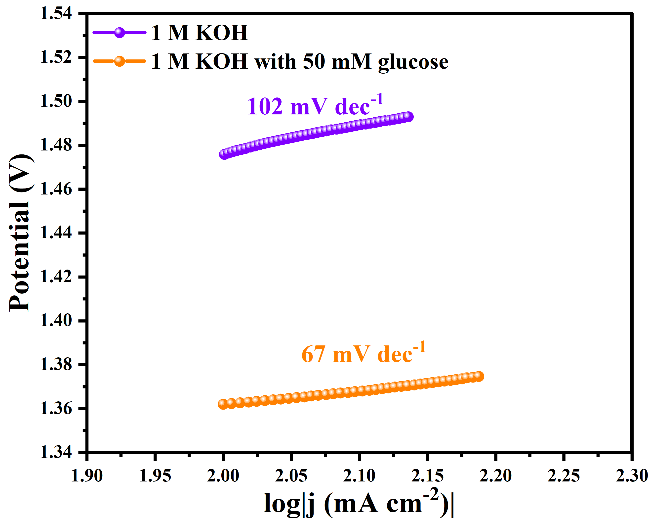


# Figure S52. Tafel plots of W,P-NiFeOOH/NF in 1 M KOH with and without 50 mM glucose.


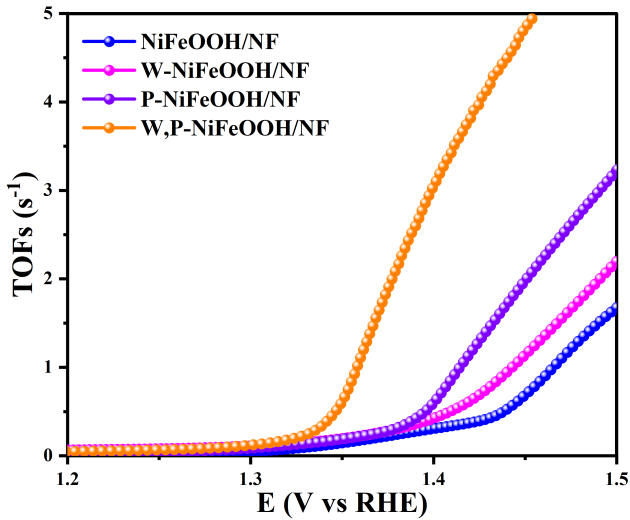


# Figure S53. GCR TOFs of NiFeOOH/NF, W-NiFeOOH/NF, P-NiFeOOH/NF, and W,P-NiFeOOH/NF.


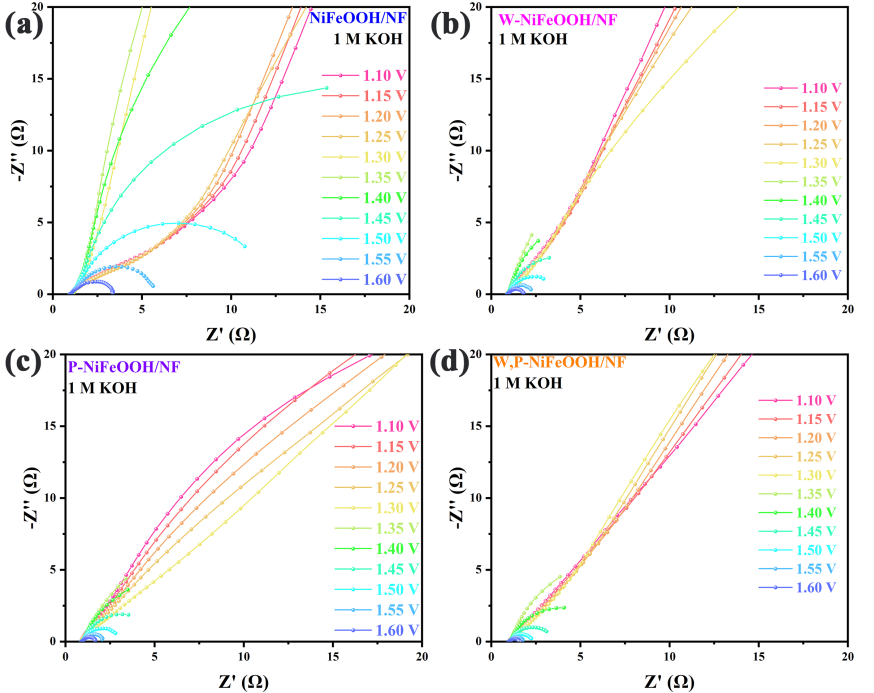


# Figure S54. Nyquist plots of (a) NiFeOOH/NF, (b) W-NiFeOOH/NF, (c) P-NiFeOOH/NF, and (d) W,P-NiFeOOH/NF in 1 M KOH.


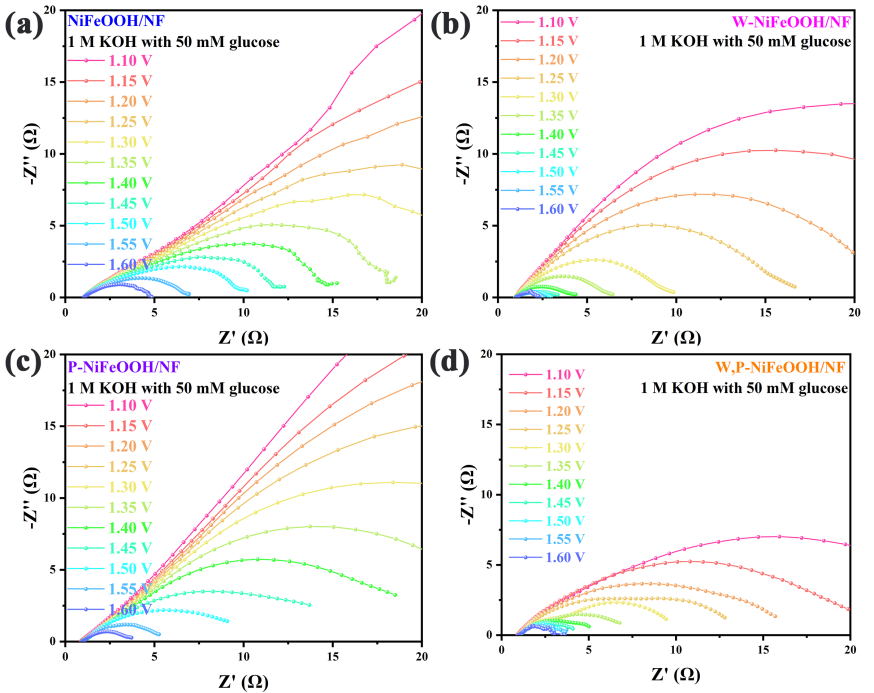


# Figure S55. Nyquist plots of (a) NiFeOOH/NF, (b) W-NiFeOOH/NF, (c) P-NiFeOOH/NF, and (d) W,P-NiFeOOH/NF in 1 M KOH with 50 mM glucose.


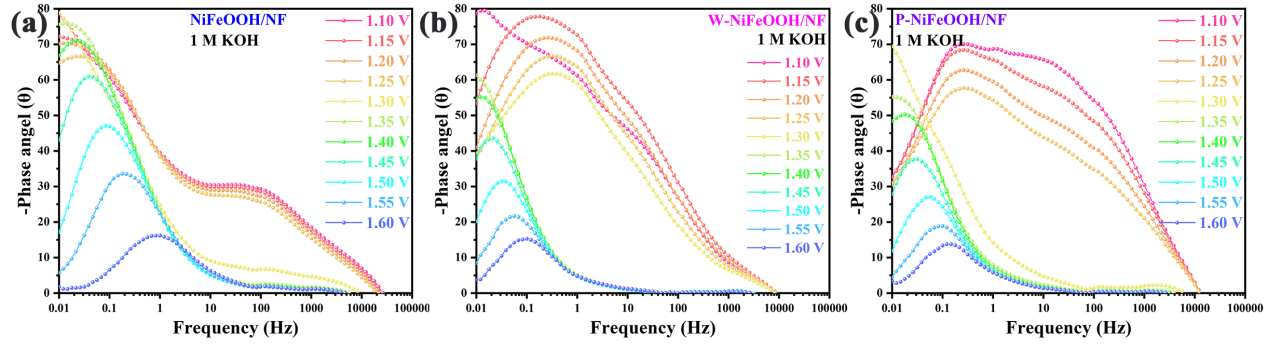


# Figure S56. Bode plots of (a) NiFeOOH/NF, (b) W-NiFeOOH/NF, and (c) P-NiFeOOH/NF in 1 M KOH.


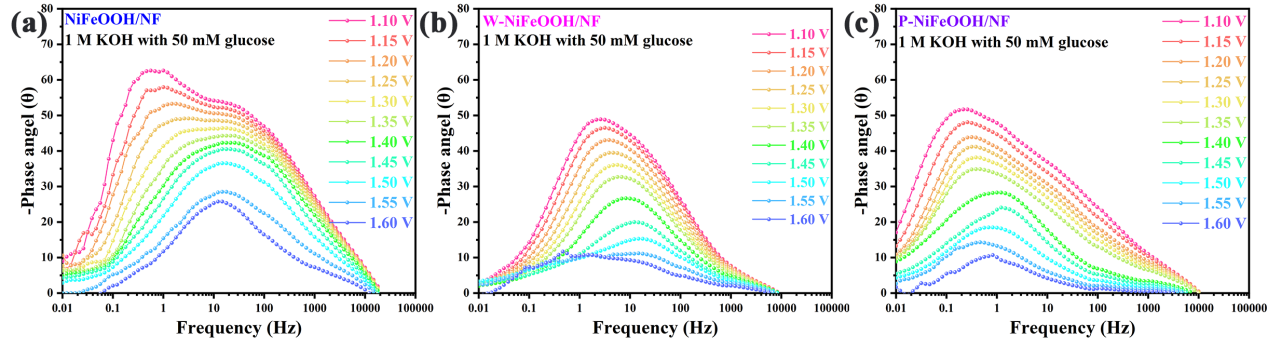


# Figure S57. Bode plots of (a) NiFeOOH/NF, (b) W-NiFeOOH/NF, and (c) P-NiFeOOH/NF in 1 M KOH with 50 mM glucose.


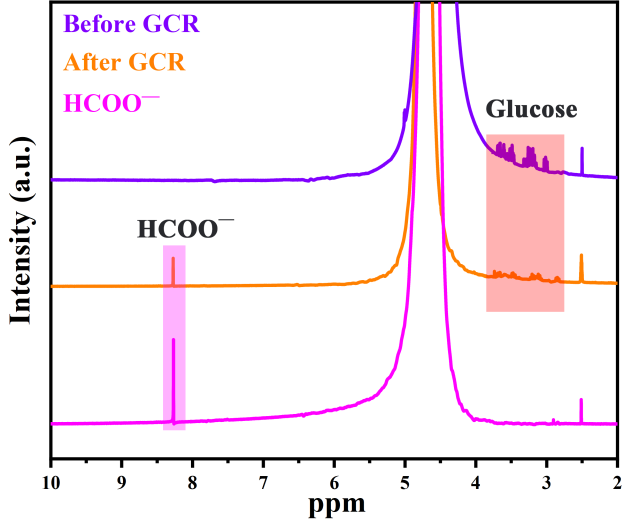


# Figure S58. ^1^H NMR spectra of the electrocatalytic conversion of glucose to formate.


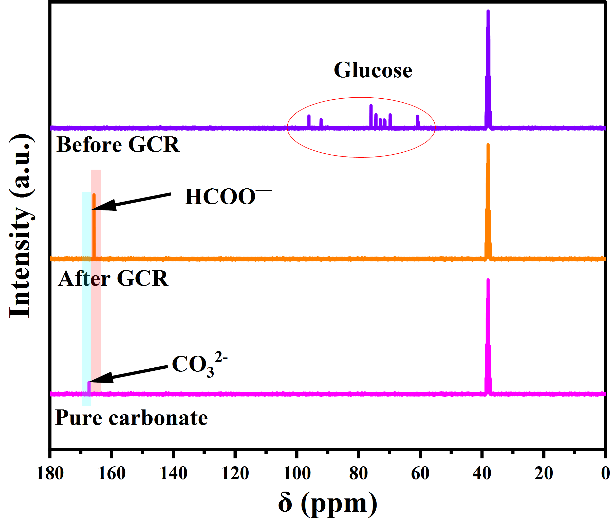


# Figure S59. ^13^C-NMR spectra of the of the final solution after electrolysis of glucose.


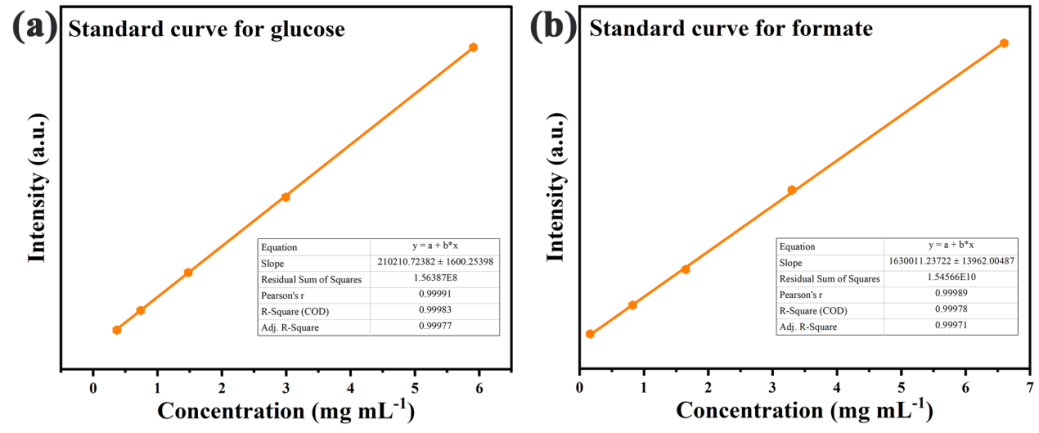


# Figure S60. Standard curves of (a) glucose and (b) formate.


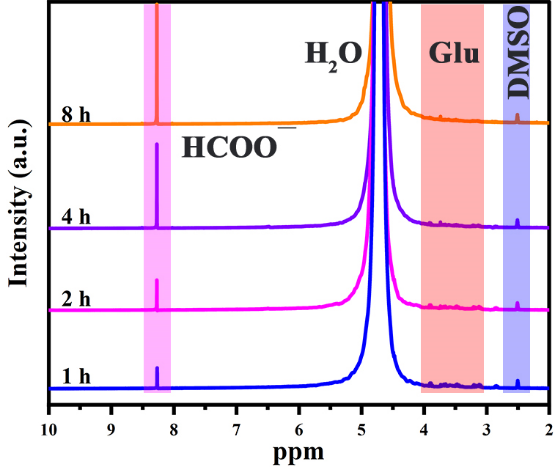


# Figure S61. ^1^H NMR measurements of glucose converted to formate for varied time.


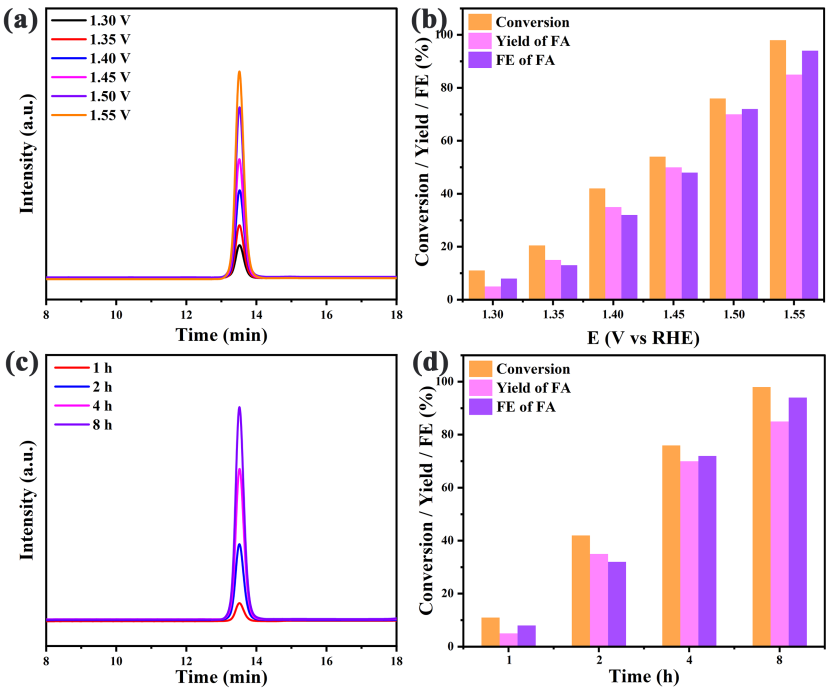


# Figure S62. (a) HPLC graphs and (b) glucose conversion of W,P-NiFeOOH/NF as an anodic electroatalyst in a two electrode alkaline electrolyzer at varied potentials; (c) HPLC graphs and (d) glucose conversion of W,P-NiFeOOH/NF as an anodic electroatalyst in a two electrode alkaline electrolyzer at varied time.


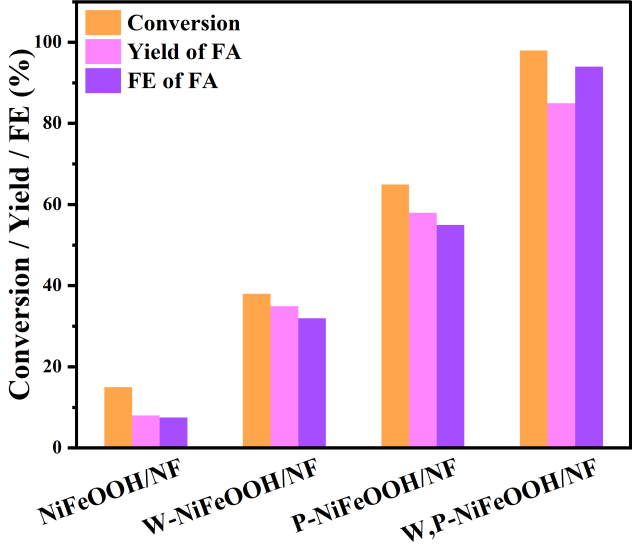


# Figure S63. Glucose conversion tests of NiFeOOH/NF, W-NiFeOOH/NF, P-NiFeOOH/NF and W,P-NiFeOOH/NF as anodic electroatalysts in a two-electrode alkaline electrolyzer at 1.55 V for 8 h.


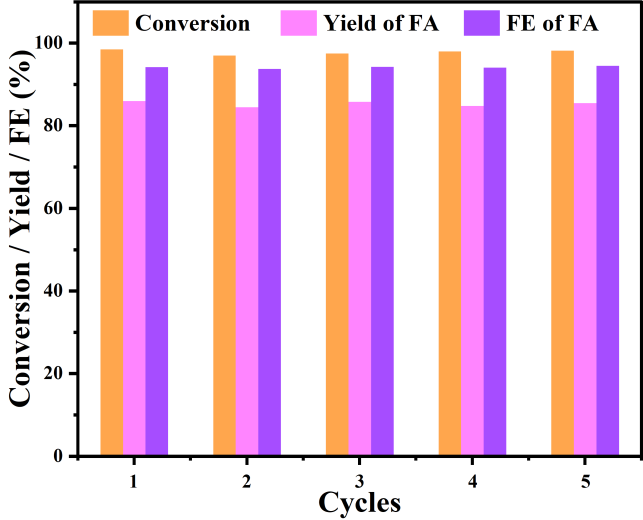


# Figure S64. Glucose conversion tests of W,P-NiFeOOH/NF as an anodic electroatalyst for 5 cycles in a two-electrode alkaline electrolyzer at 1.55 V for 8 h.

**
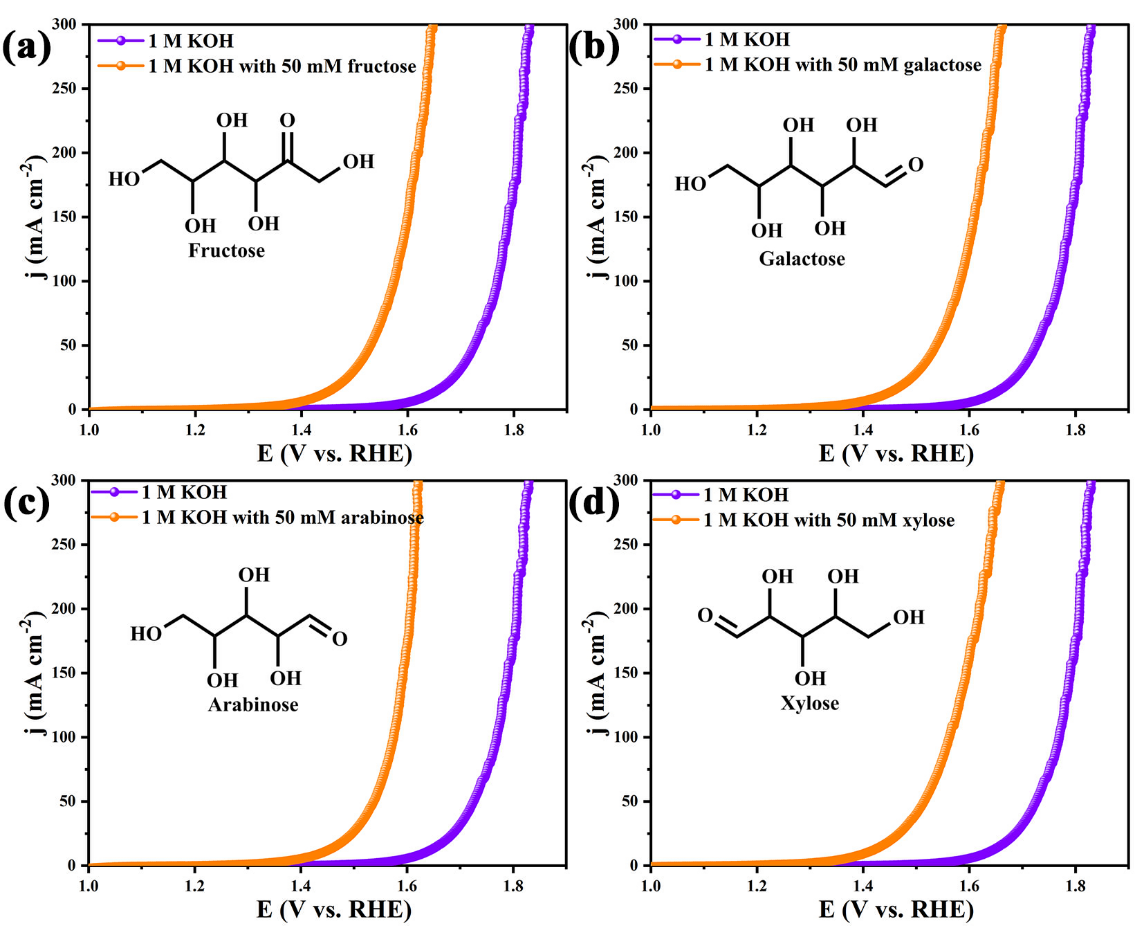
**

# Figure S65. Polarization curves of the W-NiFeP/NF||W,P-NiFeOOH/NF in 1 M KOH with and without the addition of 50 mM monosaccharide


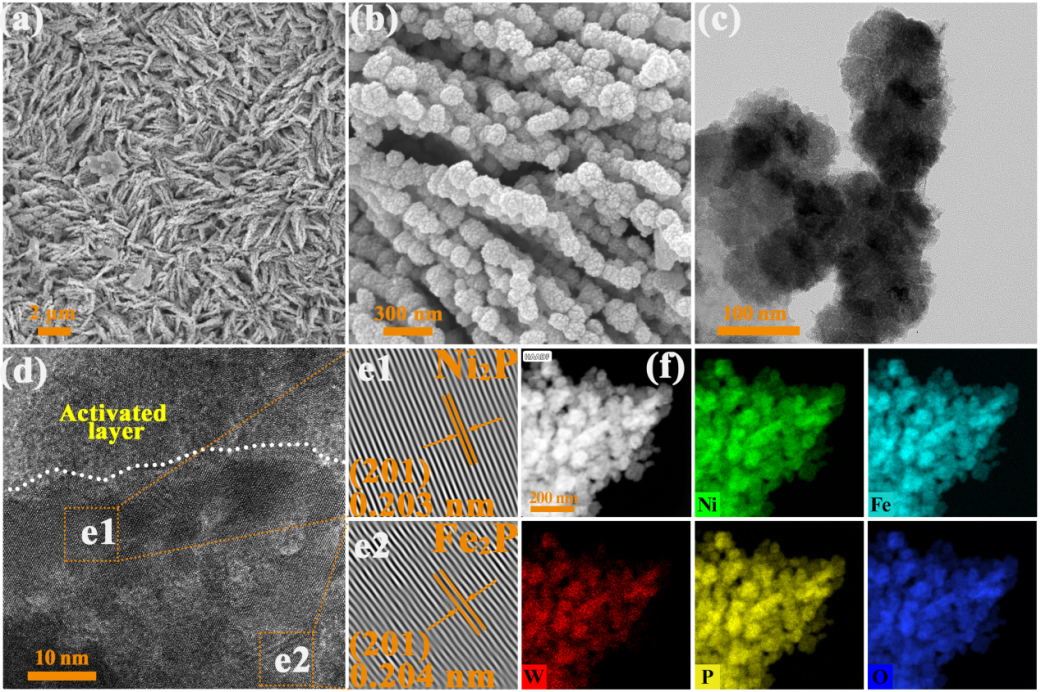


# Figure S66. (a,b) SEM and (c,d) TEM images of W,P-NiFeOOH/NF. (e1-e2) The corresponding IFFT patterns of the orange box in (d). (f) Elemental maps of Ni, Fe, W, P and O for W,P-NiFeOOH/NF after GCR-assisted overall water splitting.


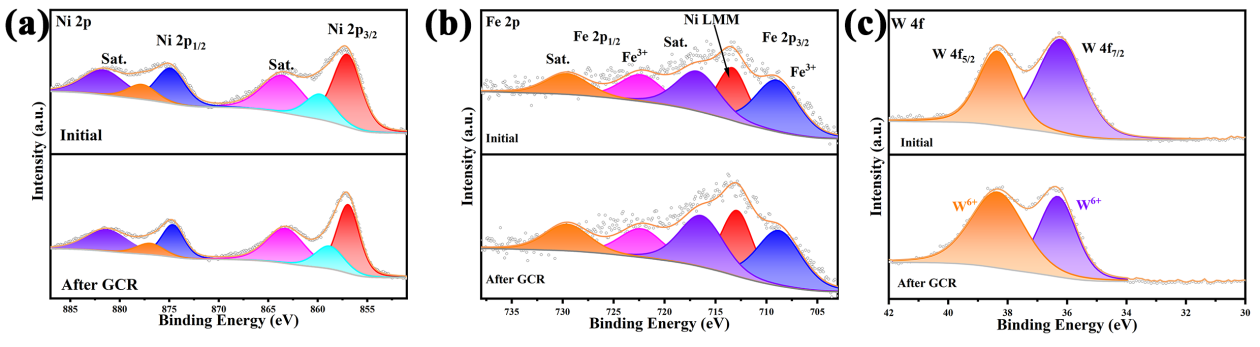


# Figure S67. High-resolution XPS scans of the (a) Ni 2p, (b) Fe 2p and (c) W 4f electrons of W,P-NiFeOOH/NF after GCR-assisted overall water splitting.


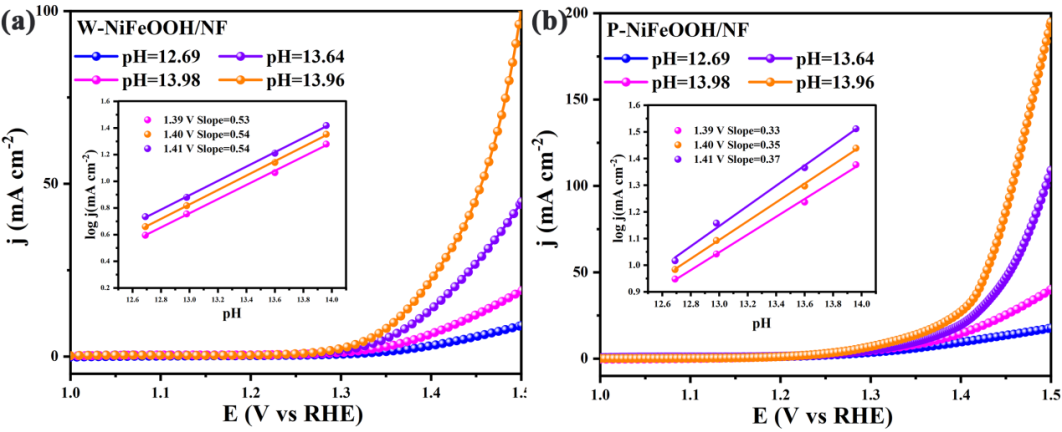


# Figure S68. GCR polarization curves of (a) W-NiFeOOH/NF and (b) P-NiFeOOH/NF in KOH and 50 mM glucose as a function of pH. Insets are the correlation between GCR activity and pH.


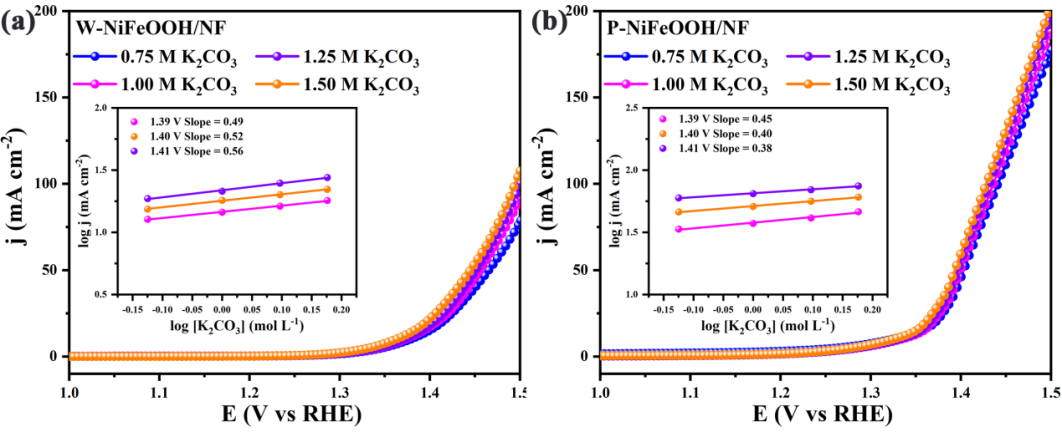


# Figure S69. GCR polarization curves of (a) NiFeOOH/NF and (b) W,P-NiFeOOH/NF at varied concentrations of K_2_CO_3_ (pH = 13.96). Insets are the correlation between GCR activity and concentrations of K_2_CO_3_.


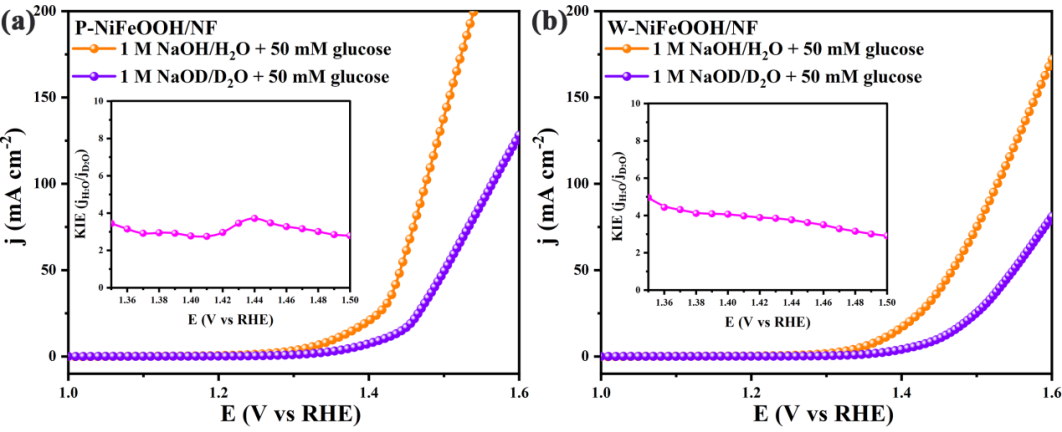


# Figure S70. GCR polarization curves of (a) NiFeOOH/NF and (b) W,P-NiFeOOH/NF in 1.0 M NaOD/D_2_O with 50 mM glucose and 1.0 M NaOH with 50 mM glucose aqueous solutions. Insets are the KIEs values at different potentials.


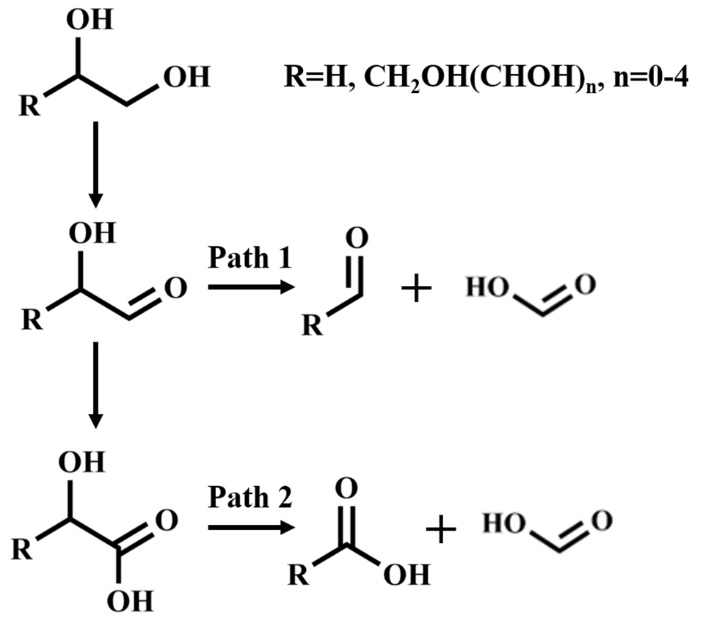


# Figure S71. Reported reaction pathways for C−C bond cleavage of polyhydroxy compounds


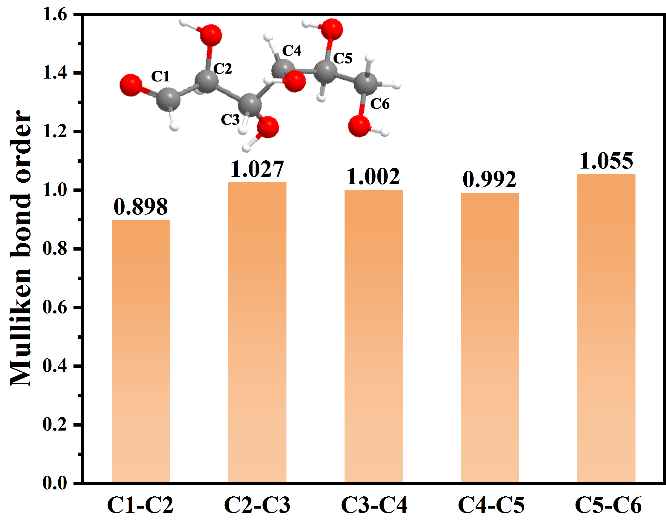


# Figure S72. Mulliken bond order of the glucose.

**
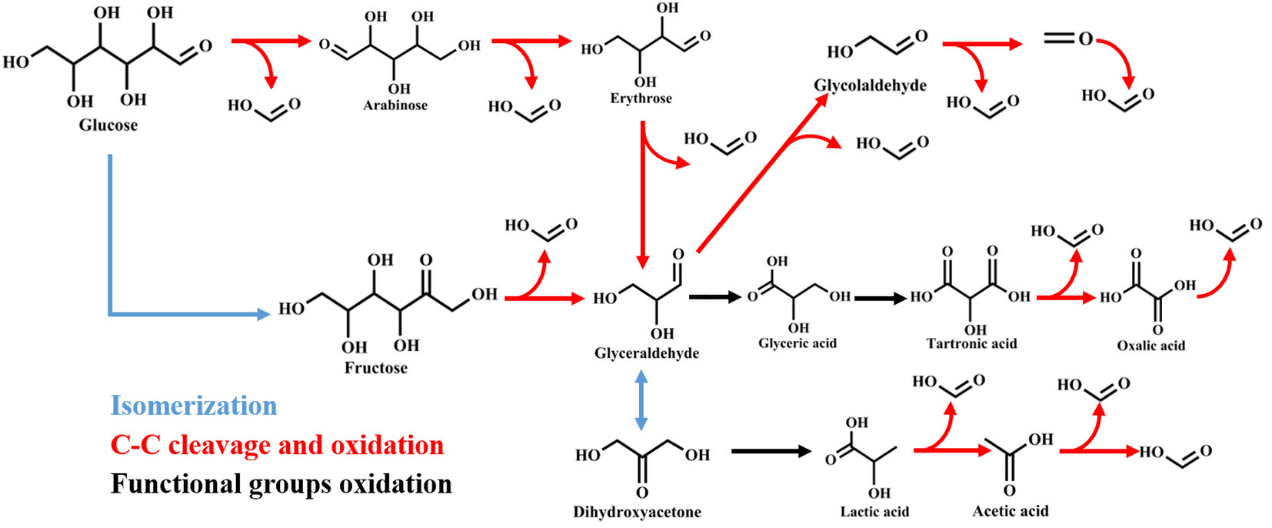
**

# Figure S73. Scheme of the possible GCR pathways.

# Table S1. 3d band center (*ε_d_*), 2p band center (*ε_p_*), and energy difference (△E) between *ε_d_* and *ε_p_* of NiFeOOH, W-NiFeOOH, P-NiFeOOH, and W,P-NiFeOOH.

| Sample | *ε_d_* (eV) | *ε_p_* (eV) | △E (eV) |
| --- | --- | --- | --- |
| NiFeOOH | -1.811 | -5.785 | 3.794 |
| W-NiFeOOH | -1.774 | -6.150 | 4.376 |
| P-NiFeOOH | -1.853 | -5.625 | 3.772 |
| W,P-NiFeOOH | -1.818 | -5.390 | 3.572 |

**Table S2.** Content of P-O species in P electron.

| **Sample** | **Content of P-O (%)** |
| --- | --- |
| W-NiFeP/NF | 89.19 |
| W,P-NiFeOOH-1/NF | 93.51 |
| W,P-NiFeOOH-5/NF | 95.63 |
| W,P-NiFeOOH-10/NF | >99 |
| W,P-NiFeOOH-20/NF | >99 |
| W,P-NiFeOOH-50/NF | >99 |

**Table S3.** Comparison of the overall water splitting performance of the catalysts in the present study with relevant non-precious metal electrocatalysts reported in the literature in 1 M KOH.

| Electrocatalysts | E_100_ (V) | Ref |
| --- | --- | --- |
| W-NiFeP/NF, W,P-NiFeOOH | 1.789 | This work |
| NiCo_2_O_4_,  NiCo(OH)_2_ | 1.95 | ACS Appl. Mater. Interfaces, 2021, 13, 45566-45577. |
| Ni_2_P-Fe_2_P | 1.811 | Adv. Funct. Mater., 2021, 31(1): 2006484 |
| Fe_0.9_Ni_2.1_S_2_@NF | 1.83 | Adv. Energy Mater., 2020, 10(41): 2001963 |
| W-Co_2_P/NF,  Mo-Co_2_P/NF | 2.0 | J. Colloid Interface Sci., 2024, 665: 152-162. |
| FNO/CPE | 1.94 | Small, 2024: 2311627. |
| MoNi_4_/MoO_2_@CW | 1.9 | Adv. Funct. Mater. 2024, 34(8): 2308337 |
| NiFe-LDH/Ni(OH)_2_ | 1.80 | Chem. Eng. J., 2021, 419: 129608 |
| Ni-Fe-K_0.23_MnO_2_ CNFs-300 | 1.81 | Small, 2020,16: 1905223 |
| Ni-Mo_2_C/NC@NF | >1.85 | Appl. Catal. B 2021, 292: 120168 |
| Co_3_O_4_@NiP_x_ | 1.915 | Chem. Eng. J., 2024: 149903. |
| Co_3_S_4_@NiFe-200/NF | 1.94 | ACS Appl. Mater. Interfaces, 2024. |
| Mo-Co_9_Se_8_/FeNiSe/NF | 1.87 | J. Colloid Interface Sci., 2024, 655: 296-306. |

# Table S4. Comparison of organic electrocatalytic conversion coupled hydrogen production of the electrocatalyst prepared in the present study with relevant non-noble metal catalysts reported in the literature.

| Electrocatalysts | Electrolyte | E_100_ (V) | Ref |
| --- | --- | --- | --- |
| W-NiFeP/NF, W,P-NiFeOOH | 1 M KOH+50 mM glucose | 1.56 | This work |
| NiMo_3_S_4_-R, NiMo_3_S_4_ | 1 M KOH+10 mM HMF | 1.58 | Appl. Catal. B Environ. 2023, 323: 122126. |
| MoO_2_-FeP@C | 1 M KOH+10 mM HMF | 1.69 | Adv. Mater. 2020, 32: 2000455 |
| Ni_3_N@C/NF | 1 M KOH+10 mM HMF | 1.63 | Angew. Chem. Int. Ed. 2019, 131: 16042. |
| NiSe@NiO_x_ | 1 M KOH+10 mM HMF | 1.88 | Appl. Catal. B: Environ.,  2020, 261: 118235 |
| NC/Ni–Mo–N/NF | 1 M KOH+0.1 M glycerol | 1.6 | Appl. Catal. B: Environ, 2021, 298: 120493. |
| Ni(OH)_2_/NF | 1 M KOH + 0.5 M methanol | 1.67 | Appl. Catal. B: Environ, 2021, 281: 119510 |
| NiCo_2_@MoO_2_/NF | 1 M KOH+10 mM HMF | >1.6 | ACS Catal. 2023, 13: 13257-13266 |
| Ni–Mo–N/CFC | 1 M KOH+0.1 M glycerol | >1.8 | Nat Commun, 2019, 10: 5335. |
| Co(OH)_2_@HOS/CP | 1 M KOH+3 M methanol | >1.65 | Adv. Funct. Mater. 2020, 30: 1909610. |
| MoO_2_-FeP@C | 1 M KOH+10 mM HMF | 1.7 | Adv. Mater. 2020, 32: e2000455. |
| O_vac_-V-Ni(OH)_2_ | 1 M KOH +0.33 M urea | >1.7 | Adv. Funct. Mater. 2023,  33: 2209698 |
| CNS-160, CNS-140 | 1 M KOH +50 mM xylose | >1.8 | Chem. Eng. J. 2022, 446: 136950. |
| Fe_0.1_-CoSe_2_/CC | 1 M KOH + 0.5 M glucose | 1.7 | Appl. Catal. B Environ, 2020, 277: 119178. |

**References**

[1] Z. Yang, Y. Lin, F. Jiao, J. Li, J. Wang, Y. Gong, *J. Energy Chem.* **2020**, 49, 189.

[2] Y. Zhang, H. Guo, X. Li, J. Du, W. Ren, R. Song, *Chem. Eng. J.* **2021**, 404, 126483.
